# Spatiotemporal reconstruction of Corded Ware and Bell Beaker burial rituals reveals complex dynamics divergent from steppe ancestry

**DOI:** 10.1126/sciadv.adx2262

**Published:** 2025-08-20

**Authors:** Quentin P. J. Bourgeois, Florian Helmecke, Louise Olerud, Igor Djakovic, M. Guadalupe Castro Gonzales, Erik Kroon

**Affiliations:** ^1^ Faculty of Archaeology, Leiden University, Einsteinweg 2, 2333CC Leiden, Netherlands.; ^2^ CISPAC - Santiago de Compostela University, Edificio Fontán, Cidade da Cultura S/N, Santiago de Compostela, Spain.

## Abstract

Understanding how the dispersal of cultural innovations intersects with the spread of genes remains a central challenge in prehistoric archaeology. Here, we examine how the third millennium BCE Corded Ware (CW) and Bell Beaker (BB) burial traditions disseminated across Europe and their relation to the influx of steppe ancestry. To investigate these spatiotemporal dynamics during one of Europe’s most transformative periods, we compiled a dataset of radiocarbon dates from 967 burials, applying kernel density estimation alongside optimal linear estimation. We show that the adoption of CW and BB funerary rites is not synchronized with, and often contradicts, the spread of steppe ancestry. Furthermore, we show that these burial traditions spread rapidly and polyfocally among dispersed communities before a brief yet continent-wide consolidation phase around 2600 BCE for CW and 2400 BCE for BB, suggesting broad, simultaneous societal changes among preliterate societies.

## Teaser

Chronological modeling shows fast, polyfocal dissemination of Corded Ware and Beaker rites before rapid Pan-European convergence.

## INTRODUCTION

Recent methodological breakthroughs in ancient DNA (aDNA) analysis have fundamentally reshaped our understanding of past demographic shifts and population movements (*1*–*5*). While these advances have substantially reshaped archaeological discourse, they have also sparked concerns regarding the uncritical use of genetic data to infer cultural transformations (*6*–*9*). These advances require an equally thorough appreciation of how specific populations intersect with cultural phenomena to avoid oversimplifying past societies into mere bio-cultural entities (*8*, *9*) or, conversely, entirely separating cultural and biological processes (*10*).

A particularly illustrative example is provided by the emergence of Corded Ware (CW) and Bell Beaker (BB) burial rites across Europe during the third millennium BCE. Recent aDNA studies have linked these CW and BB groups to large-scale population movements from the Eurasian steppe, which significantly affected older Neolithic populations on continental (*2*–*5*) and regional scales (*11*–*13*). During the same time period, we see continent-wide changes in burial practices, characterized by a similar set of principles, with a new emphasis on burying and equipping individuals in a stereotypical fashion, emerging across CW and BB groups (*14*–*17*). These genetic and cultural transformations have further been linked to broader societal changes, such as the introduction of Indo-European languages (*2*), the appearance of a mostly binary gender system persisting until today (*18*), and a noticeable uptick in individual mobility (*5*, *19*–*21*).

The timing and pace of these developments, however, remain hotly debated with scholars arguing either for a rapid (*22*) or a gradual (*14*, *23*) process. These alternate temporal dynamics imply radically different mechanisms behind the observed changes. In addition, the debate about the timing of these developments also gives rise to different arguments about potential causes, such as climatic changes and societal changes on the Eurasian steppe (*14*, *24*, *25*). A complicating factor in this debate is the current lack of a precise chronology for the initial spread and eventual proliferation of these new burial traditions across Europe. Current studies either accept the start and end dates for the CW and BB complex from old regional chronologies (*26*–*28*) or work with sparse subsets of “oldest points” for CW and BB burials (*22*). Neither approach makes use of new analytical methods for the analysis of radiocarbon dates (*29*, *30*) or of the considerable number of new radiocarbon dates on individuals from the third millennium BCE published over the past decade (*2*, *4*, *12*, *13*, *31*).

In addition, archaeologists still continue to debate the nature of the CW and BB complex, with authors emphasizing both its heterogeneity and its homogeneity (*32*, *33*). Nevertheless, a common denominator of distinct shared burial practices can be discerned at the heart of both of these phenomena. Across vast distances, mourners buried deceased individuals with a similar set of grave goods and conforming to distinct practices such as a semiflexed posture and gender rules (*14*, *16*, *17*). We argue that these funerary events reflect deliberate choices made by the mourners, consciously shaping how the deceased were represented in death. The expansion of these shared practices is, in turn, linked to the migration patterns inferred from genetic evidence (*24*). Yet until now, a solid chronology of their appearance, and how that correlates with population movements, has remained elusive.

To address this knowledge gap, we compiled a continent-wide dataset of radiocarbon dates associated with CW and BB burial rites. We applied rigorous sample selection criteria and collected reliable radiocarbon dates associated with 967 burial events across Europe from the third millennium BCE: 453 CW burials and 514 BB burials (see data S1 and S2; data S3 and S4 for excluded radiocarbon dates; see Methods). We analyzed these dates with a combination of kernel density estimation (KDE) (*29*) and optimal linear estimation (OLE) modeling (*30*, *34*, *35*) (see Methods).

### Fast polyfocal emergence of CW and BB

**Fig. 1.**
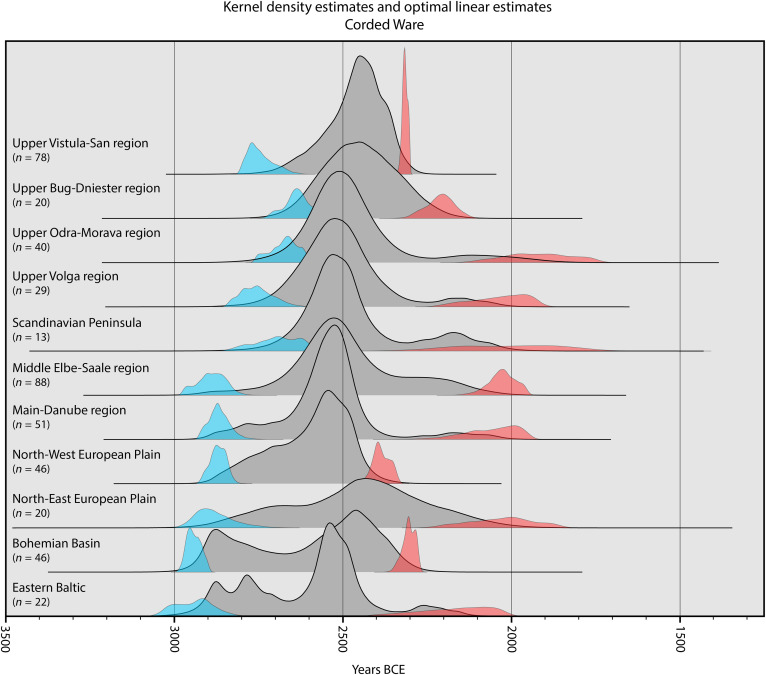
Ridgeline plot for CW burial events. KDE (gray) and OLE (blue, start; red, end) for radiocarbon-dated CW burial events (see data S1 and supplementary text) in different regions (see Methods and Fig. 6). All distributions have been integrated to facilitate comparisons.

**Fig. 2.**
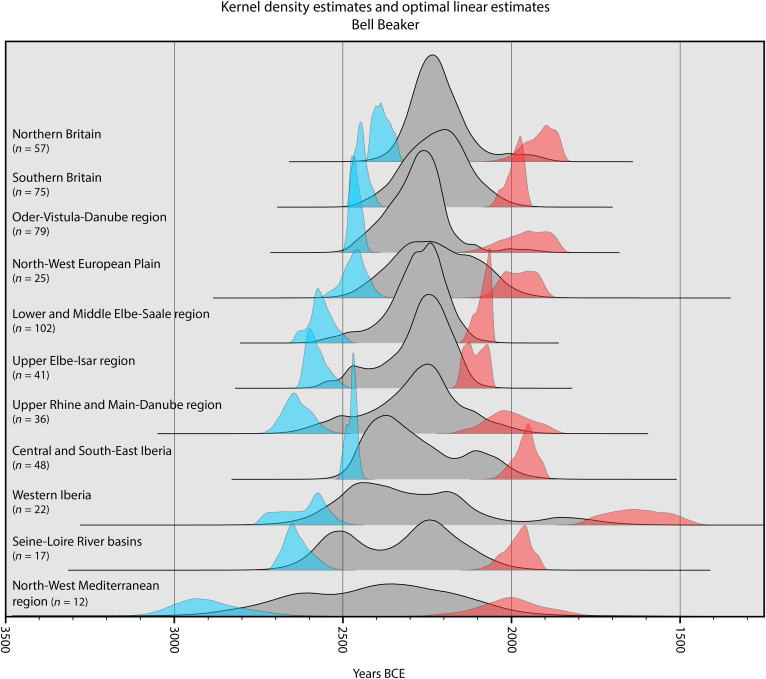
Ridgeline plot for BB burial events. KDE (gray) and OLE (blue, start; red, end) for radiocarbon-dated BB burial events (see data S2 and supplementary text) in different regions (see Methods and Fig. 6). All distributions have been integrated to facilitate comparisons.

To understand the broader spatiotemporal development of CW and BB burial rites, we conducted a detailed Bayesian analysis of the available radiocarbon dates and created KDEs and OLEs for 22 archaeologically distinct regional groups of BB/CW burial rites (Figs. 1 and 2; see Methods and supplementary text). The KDEs represent the overall probability density of radiocarbon dates within a given time period, while the OLEs estimate the earliest or latest possible occurrence of a phenomenon based on the distribution of the available chronological data (*30*). Although the calibration curve in the third millennium BCE can influence the age range of radiocarbon measurements, our assessment of its effect on the Bayesian modeling framework indicates only a minor impact (see Methods and supplementary text).

**Fig. 3.**
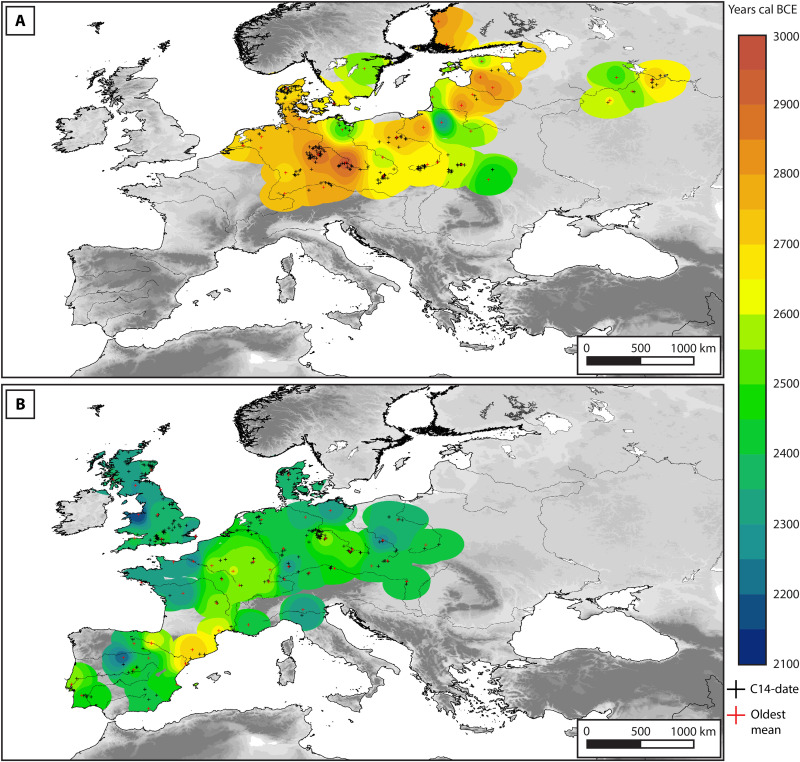
Interpolation map of the radiocarbon-dated CW and BB burial events with the oldest modeled mean dates. The interpolation surfaces for CW and BB are shown in (**A**) and (**B**), respectively. Black crosses are the find locations of all radiocarbon-dated burial events (see data S1 and S2). The interpolation map was produced by applying inverse distance weighing (IDW) to a subset of these data (red crosses). This subset consists of the radiocarbon dates burial events with the oldest, modeled mean within a 100-km radius and *A* ≥ 60% in the KDE.

In contrast to prevailing narratives of an initial east to west spread of CW (*36*) with a later BB emergence (*4*), we find evidence for a near synchronous and early emergence of both CW and BB burial rites across the continent. For CW, the OLEs suggest a notably early emergence of the burial rite in the Bohemian Basin around 2939 BCE [mean estimate, 95.4% confidence interval (CI) range: 3006 to 2886 BCE; Fig. 1] and the Eastern Baltic around 2944 BCE (mean estimate, 95.4% CI range: 3112 to 2715 BCE; Fig. 1). The minimal temporal difference between these far-flung regions indicates near simultaneous emergence of these burial rites in disparate geographic locales. Further underlining this fast, near synchronous, emergence is the fact that the mean estimates for the beginning of CW burial rites in the Baltic and Bohemia are within 50 years of the estimated emergence of CW across the European Plain. This points to a broad synchronicity in the emergence of CW between different locales ~2940 to 2875 BCE and is supported by the distribution of the oldest modeled mean ages (see Fig. 3A). The spatial interpolation and the KDEs show that the oldest CW burials appear in isolated, disparate pockets across the Baltic and Central Europe and that CW spreads rapidly from these initial sources. However, our analysis also reveals that, in Scandinavia, but, especially, in Eastern European regions closest to the steppe, the estimated emergence for CW burial events are substantially younger than those in Central Europe. The mean OLE estimates for these regions suggest an emergence of CW burial events around 2750 to 2650 BCE (Fig. 1). These observations indicate that the spread of CW does not follow the traditional “wave of advance” model, which postulates a single, oldest core from which a phenomenon radiates out (*22*, *36*). We find that the CW burial rite is a polyfocal phenomenon emerging nearly simultaneously at multiple locations.

An unexpected outcome of the OLE is that the earliest estimated emergence for BB burial rites is contemporaneous with the earliest estimates for CW burial rites. The mean OLE estimates for BB burials along the North-West Mediterranean Coast fall at 2901 BCE, albeit with a much broader error margin (mean estimate, 95.4% CI range: 3232 to 2604 BCE; Fig. 2). Admittedly, coverage in this region is poor, and this is reflected in the higher degree of uncertainty in both the OLE and the KDE. Nevertheless, this early mean estimate is caused by a cluster of early BB burials around the Pyrenees with secure radiocarbon dates falling well into the first half of the third millennium BCE. This finding challenges current ideas on the emergence of BB, which generally involve a younger and more western origin point in Portugal based on radiocarbon dates in settlement contexts (*37*). Following this initial early emergence, BB burial rites then spread from this old core around the Pyrenees in western and northern directions (see Fig. 3B). We do not see evidence for a previously hypothesized maritime route for the spread of BB burial rites (*38*), but rather a dissemination along the rivers Rhône and Rhine and their tributaries. The spread of BB material culture in settlement contexts may well follow a maritime route (*39*), but this is not evident from the characteristic BB burial rites.

Our analysis shows that initial BB and CW burial rites may have emerged contemporaneously at different sides of the European continent. It should be noted that these early burials do not feature all practices that are considered typical for fully fledged BB. They do feature classical BB artifacts, such as the eponymous BBs, tanged copper daggers, and the body placed in a semiflexed position, but are often located in caves or preexisting megaliths and lack the distinctive gendered body positions that characterize later BB relative to CW. This suggests that certain highly typical BB practices may appear only later on, potentially around 2600 BCE in Eastern France and Southern Germany, in interaction with CW communities, as an effort of mourners to distinguish these burials from the then still spreading CW burial rites by effectively inverting the typical CW gendered body positions.

Our estimated emergence of these highly distinct burial rituals now informs us on the nature of the spread of these rituals. First, CW burial rituals emerge latest in the regions closest to the steppe. This stands in stark contrast to the proposed dispersal of steppe ancestry from east to west (*5*, *24*, *36*), which is thought to have been mediated by users of CW burial practices in these regions. Second, we hypothesize that the earliest instances of BB burial ritual evolved among communities without steppe ancestry (*4*) as steppe ancestry can only be detected in Iberia toward the end of the third millennium BCE. Groups with steppe ancestry more toward the north then co-opted these new BB burial rites and adapted them within their own ritual frameworks.

### Spread of steppe ancestry asynchronous to CW or BB burials

These findings of the earliest emergence of CW and BB burial rites and their spread across Europe appear to contradict the findings of aDNA studies that show that individuals buried according to these burial rites tend to have high steppe ancestry (*2*, *3*, *5*, *13*). To further explore this apparent disconnect, we tested the spatiotemporal correlation between the predicted arrival date of CW and BB burial rites in this study (Fig. 3) and the predicted arrival dates of steppe ancestry in (*24*) and (*5*). Our assumption is that, if the spread of steppe ancestry predicts the spread of CW and BB burial rites, then a monotonic relation should exist between the age of the earliest CW and BB burials and the arrival date of steppe ancestry. To test this assumption, we performed a Kendall’s Tau-b test (see Methods). For the earliest occurrence of CW burial rites, our test returns a correspondence of 0.12 (*P* = 0.01) to the predicted arrival dates of steppe ancestry by Racimo *et al.* (*24*) (see Fig. 1). The correspondence between the arrival dates of BB burial ritual and the arrival date of steppe ancestry according to Racimo *et al.* (*24*) is −0.08 (*P* = 0.07). Relative to the predicted arrival dates of steppe ancestry in (*5*), we find values of 0.12 (*P* = 0.05) for CW and 0.2 (*P* = 0.03) for BB burials. We, therefore, find that the predicted arrival of steppe ancestry does not correlate with the predicted arrival of CW and BB burial rites. These low correspondence values from Kendall’s Tau-b indicate that the order in which steppe ancestry appears in regions across Europe is not related to the order in which CW or BB burial rites appear in these regions. This is also evident from a comparison of the interpolation surfaces in Fig. 3 to the temporal distribution maps of steppe ancestry in figures 5 and 8B of (*24*) and figure 3 of (*5*). These studies show that the arrival dates for steppe ancestry are the oldest in Eastern Europe and gradually become younger the further west one is from the Pontic Caspian steppe (*36*). This contrasts with the appearance of CW, which is generally oldest in Central Europe and the Eastern Baltic and youngest toward the steppe. Similarly, BB burial rites first appear on the North-West Mediterranean Coast and last in Northern Britain (Fig. 3), whereas the arrival dates of steppe ancestry are predicted to be much older in Northern Britain than on the North-West Mediterranean Coast (*5*, *24*).

**Fig. 4.**
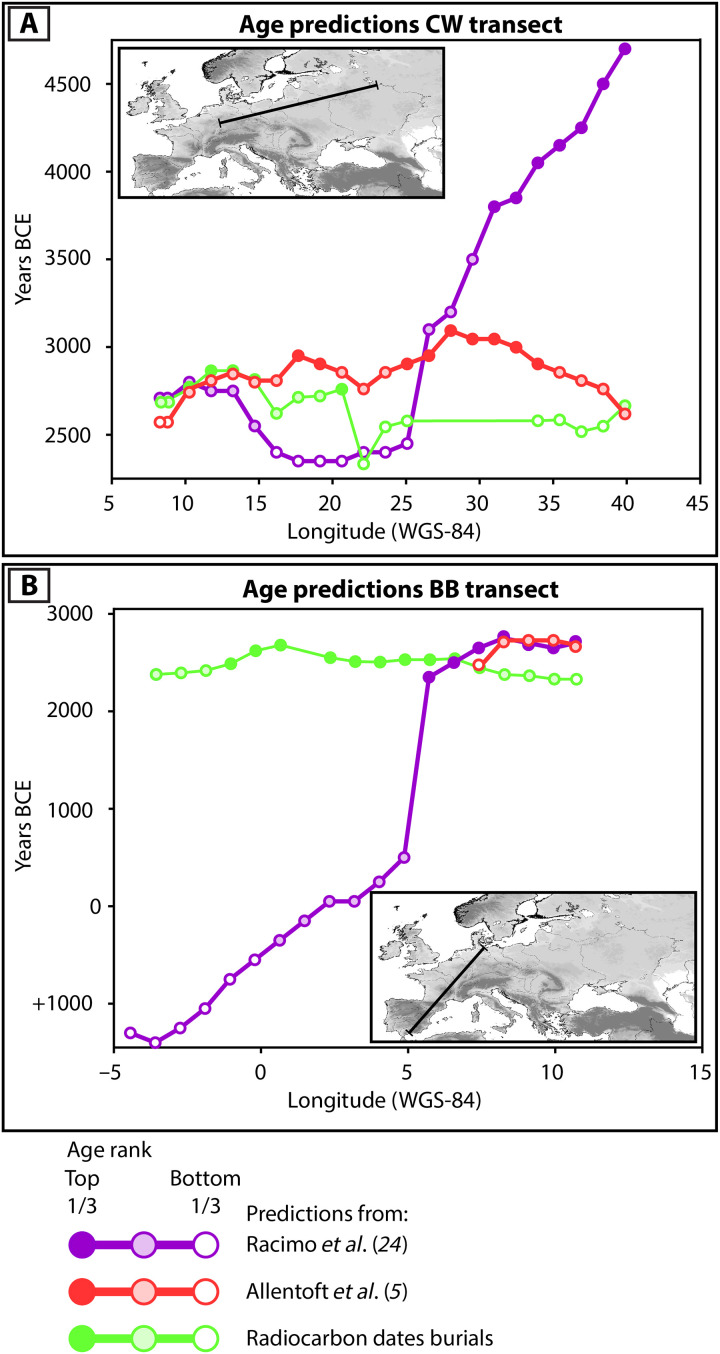
Predicted arrival date of steppe ancestry and burial rites across Europe. Arrival dates for burials based on the interpolation in Fig. 3. Arrival dates for steppe ancestry based on interpolations from Racimo *et al.* (*24*) and Allentoft *et al.* (*5*). Predicted arrival dates for steppe ancestry show an east-west cline with the arrival dates for steppe ancestry generally becoming younger westward. This causes a mismatch both in absolute age and age ranks with the arrival dates for CW (**A**) and BB (**B**) burials.

To further clarify this outcome, we visualized the differences in age rank and absolute ages of the points in the comparison along two transects for CW and BB, respectively (Fig. 4; see Methods). These transects show that the arrival of steppe ancestry in Europe is a drawn-out process spanning half a millennium or more, whereas the spread of CW and BB burial rites happens on a much shorter time scale, within two or three centuries at most. These two processes may coincide in certain areas in terms of absolute time, notably in the Central German Uplands and the Bohemian Basin for CW (see Fig. 4).

Our results indicate that the earliest manifestations of CW and BB burial practices arise in regions farthest from the presumed genetic source population. For BB in particular, these oldest burials emerge independently of steppe ancestry, something already observed in the earliest papers on the genetics of BBs (*4*, *40*), ultimately spreading in a direction counter to the spread of steppe ancestry. This outcome underscores a perspective on migration that is more complex than a straightforward driver of cultural innovation; major population shifts may create conditions that facilitate the expansion of these practices, yet these practices do not propagate purely because steppe ancestry does. Similarly, for CW, the initial occurrences appear in the western and northern reaches of the phenomenon, only later extending back toward the presumed genetic origin (*1*). This pattern further demonstrates how the interplay between migration and cultural expression transcends a direct correlation.

### Slow initial dissemination, rapid consolidation, and long coexistence

We further show that the arrival of CW and BB burial rites does not result in their immediate, widespread adoption. The KDEs indicate that the highest probability densities for both CW and BB burial rituals appear centuries after the starting dates for these burial rites as indicated by the OLE. These peaks appear around 2600 to 2400 BCE for CW and 2400 to 2200 BCE for BB and do so irrespective of the starting dates for these burial rites in a region (see Figs. 1 and 2). We should be careful not to interpret these probability functions as population densities (*29*). However, they do suggest that the expected number of burial events in the initial phase is low, followed by a widespread increase in the number of radiocarbon-dated burial events in all regions during these two peak phases. Thus, our observed pattern implies that the initial spread of CW and BB burial rites was gradual and localized, taking centuries to gain widespread traction. The notable exceptions to this pattern are the Eastern Baltic and in particular the Bohemian Basin, which show a tight spatiotemporal cluster of CW burials around 2900 to 2800 BCE (Fig. 1).

The initial low probability density in the KDEs may also reflect the coexistence of CW and BB burial rituals with each other and with other established burial practices. Recent studies have shown that CW and BB burial rites are coeval with those of earlier groups in multiple regions (*23*, *41*–*44*). This proposed coexistence could offer part of the explanation behind the resurgence of early farmer ancestry in later time periods observed in the genetic data (*10*, *13*). Our findings underline a culturally dynamic and heterogeneous third millennium BCE.

We also find that the temporal distance between the moment at which CW and BB burials rites find broad acceptance is relatively short (~200 years at most) in all regions. Within two centuries, rapid, broad shifts took place in notions about death, identity, and burial. This underscores a fundamental, deeper shift in underlying societal values, beliefs, and social structures across Europe. We argue that these cultural transformations were not isolated events, but part of a broader, interconnected process of social change sweeping across Europe on a scale hitherto unseen. The strong resemblance observed in the burial rites throughout Europe accentuates the remarkable speed with which such fundamental notions could spread among these preliterate societies. This spread may have been facilitated by social conditions, demographic pressures, or exponential population growth. Nevertheless, the swift adoption of new burial practices across vast territories must not be seen as an isolated phenomenon but more as a reflection of dynamic networks whose structure was amenable to the rapid embrace of novel ideas, enabling the spontaneous emergence and quick dissemination of this new burial convention (*45*, *46*).

**Fig. 5.**
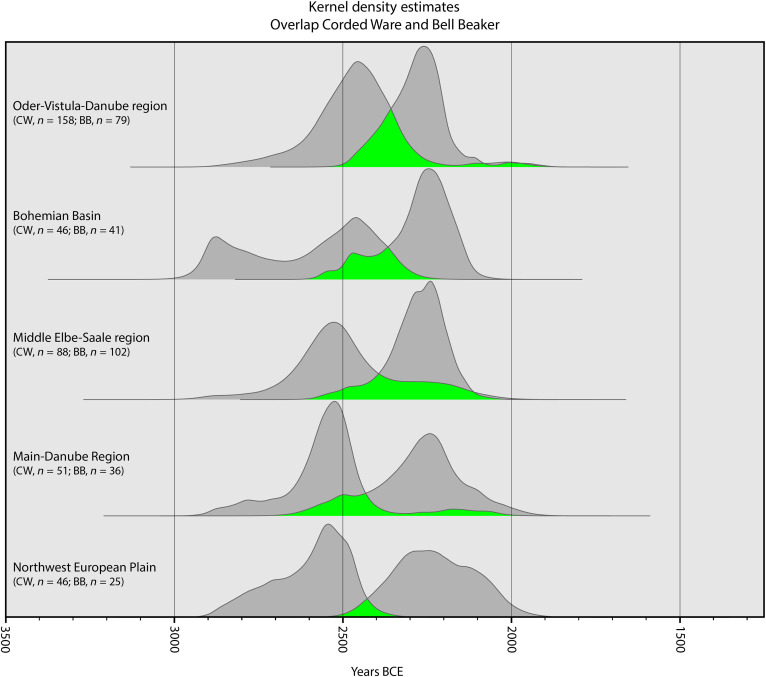
Overlap of the KDE (in gray) for radiocarbon-dated CW and BB burial events in various regions (Figs. 1 and 2). Overlap in green. The KDE for CW in Eastern Europe is a combination of the groups Upper-Vistula-San region, upper Bug-Dniester region, Upper Odra-Morava region, and Northeast European Plain in Fig. 1, which matches the spatial extent of the BB group Eastern Europe in Fig. 2 (see Fig. 6).

In addition, the KDEs demonstrate a marked chronological overlap between CW and BB funerary practices in several regions (Fig. 5). This is corroborated by the latest estimated occurrence for CW and the earliest estimated emergence for BB, indicating an overlap that potentially lasts multiple centuries (see Figs. 1 and 2). Furthermore, our KDEs show that CW burial rites did not fully vanish with the appearance of BB burial rites but persisted, particularly within Central Europe where archaeological evidence also supports the coexistence of CW and BB burial customs (*47*–*49*). This finding challenges the simple chronological sequences reconstructed between these burial rites and points to a complex cultural mosaic where diverse funerary traditions and social structures coexisted and interacted.

### Abrupt end with advent of tin bronze?

The decline of BB burial rites has long been debated in light of the emergence of stratified societies during the Bronze Age (*50*). On the basis of the KDEs of BB burial rituals, we find an abrupt decline in probability densities of BB burials around 2200 to 2000 BCE across the continent. This sudden decline coincides with a marked reshaping of European-wide trade networks and the advent of tin-bronze networks (*51*). The introduction of tin-bronze technology and the consequent reconfiguration of preexisting trade routes likely played a pivotal role in disrupting established cultural practices.

Long after the initial decline seen in the KDEs, we see a continued presence of some dated CW and BB burials at the very start of the second millennium BCE (see Figs. 1 and 2). This is in line with various studies that suggest that BB burial rites are followed by regional burial traditions with a degree of overlap, such as proto-Únětice (*49*), Barbed Wire (*52*), and Food Vessel (*53*), creating a complex mosaic of more regional cultural traditions. Thus, in the second millennium BCE, the previously widespread stereotypical burial practice of the third millennium BCE (*54*) now diverges into distinct regional variants, while wealthier burials continue to reference the stereotypical older traditions as, for example, proposed for Early Bronze Age Únětice elite burials (*55*). These enduring references highlight the ongoing significance of the third millennium burial ritual, even as cultural norms underwent substantial transformation.

### Population genomics and cultural dynamics

A key finding of our study is that the earliest instances and initial spread of CW and BB burial rituals did not align with the presumed trajectories of the spread of steppe ancestry; rather, they disseminated along divergent or even opposing routes. The rapid, polyfocal emergence of the typical burial ritual among CW groups suggests that new ideas were invented and adopted by interconnected but disparate communities. Within these dispersed populations, the reification of these distinct burial rituals probably functioned as a cornerstone of group identity, strengthening social cohesion through shared mortuary customs (*16*, *17*). In the case of CW, the perception of highly dynamic and dispersed groups is corroborated by substantial genetic heterogeneity, even surpassing genetic distance among modern Europeans, observed in the earliest CW users in Bohemia (*13*). This high heterogeneity is a reflection of people with diverse ancestries, including those without steppe ancestry, coexisting within a single region and even sharing the same cemetery, inventing and adopting a distinct burial style. Notably, Papac *et al.* (*13*) model one of the likely sources of genetic ancestry among early CW in Bohemia as originating in Latvia, the other area where we see a relatively high density of early radiocarbon dates in our models and which is one of our predicted earliest centers for the emergence of CW burial practices.

By contrast, early BB burial rituals, with their distinct focus on archery equipment, copper artifacts such as tanged daggers, and the eponymous bell-shaped beaker, appear to have originated in populations with no steppe ancestry as observed previously (*4*). Once this new burial rite had developed and spread, groups with steppe ancestry co-opted and adapted it within their own ritual frameworks. This adoption and transformation likely occurred along the Rhine (*22*) and may have been mediated by a substantial demographic event that also affected the spread of BB toward the British Isles (*4*).

In both CW and BB, the earliest expansion of these distinct burial practices likely occurred among small, widely dispersed communities, as indicated by the low probability densities in our KDE models. With the notable exception of Bohemia, CW burials did not appear in large concentrations during this early phase. Likewise, there is little evidence for substantial clusters of radiocarbon dates associated with early BB burials before 2500 BCE, implying that these groups also remained relatively sparse for several centuries.

The watershed moment in European prehistory is then identified with the widespread adoption of these burial rites around 2600 BCE for CW and 2400 BCE for BB. This points toward crucial societal transformations in which diverse societies across Europe adopt a singular, widely accepted and recognized burial tradition. On the basis of our KDE models, we propose that, once a certain threshold of connectivity was achieved (*46*), these burial customs spread swiftly through dynamic communication networks, sometimes moving counter to the expansion of steppe ancestry. These networks and their increased degree of connectivity likely played a crucial role in generating tipping points that lead to the rapid spread of social conventions (*45*, *46*). Recent advances in identity by descent (IBD) have demonstrated a substantial expansion of long-distance connections across Europe in the third millennium BCE (*1*, *5*), indicating a higher level of genetic interconnectivity and an increase in personal interaction networks than in preceding periods. Although these IBD linkages highlight individual mobility on a personal scale, they also emphasize how this increased connectivity facilitated the swift diffusion of cultural practices. In this way, heightened mobility not only shaped individual experiences but also underpinned the tipping-point dynamics that drove the widespread adoption of CW and BB burial rituals.

Another key insight of our study is the marked temporal overlap between CW and BB rites, particularly in Central Europe, suggesting the existence of parallel communities maintaining distinct traditions. This differentiation is evident in the burial rites, particularly through stark contrasts in grave goods and gender-specific orientations of the body, indicating a conscious separation of identities and beliefs. This interpretation of complex interactions between contemporaneous burial rites deviates substantially from previously assumed models of a uniform, linear succession of traditions through cultural assimilation.

Following nearly a millennium of cultural prevalence, both CW and BB practices experienced a rapid decline within just a few centuries, a period concurrent with other substantial shifts such as the emergence of tin-bronze technology, a reshaping of trade networks, and the spread of domesticated horses (*51*, *56*). Yet this transformative era, while marking a departure from established burial traditions, did not result in an outright rupture. Instead, we observe a transformation where elements of CW and BB burial rites continue to be referenced in high profile burials, showcasing the enduring influence of these traditions on subsequent European communities.

The emergence of CW and BB burial traditions among preliterate societies represents one of the most fundamental transformations in European prehistory. The widespread adoption of these burial traditions offers one of the earliest examples of how a decentralized social system can converge on a shared convention (*45*, *46*). While their genetic history has garnered considerable attention, a nuanced examination of the spatiotemporal dynamics of CW and BB is essential to avoid conflating past communities with narrowly defined bio-cultural entities or separating cultural and biological processes entirely. By integrating cultural and genetic data, as exemplified by the CW and BB burials that we examined in this study, we reveal complex spatiotemporal dynamics governed by increased mobility, communication networks, and evolving identities. Investigating how these funerary practices arose, spread, and ultimately changed amidst the largest documented migration event in European prehistory illuminates the intersection of genetic and cultural information, highlighting the need for a holistic perspective to fully grasp these transformative developments.

## METHODS

### Radiocarbon dating CW and BB burial events

We conducted a continent-wide survey of CW and BB burials with reliably associated radiocarbon dates. In total, we collected radiocarbon dates from 967 burial events across Europe: 453 CW burials and 514 BB burials (see data S1 and S2). We included only radiocarbon dates with a clear and direct association with a burial event attributed to CW or BB in our analysis. In doing so, we followed the polythetic framework proposed by Vander Linden (*15*) and Furholt (*33*, *57*) and defined a set of recurring attributes. These attributes concern the construction of a burial mound sometimes accompanied by a palisaded ditch, while the deceased was placed in a semiflexed position, often following sex-specific orientations, with males and females placed in opposing directions. In the case of CW burials, distinct grave goods included battle axes, flint and stone axes, flint blades and daggers, and ceramic vessels including beakers in CW style and amphorae. BB burials included finds of copper daggers, BB-style beakers, wristguards, flint arrowheads or flakes, V-perforated buttons, and, more rarely, cushion stones or golden basket earrings.

These stringent selection criteria mean we rejected a substantial amount of radiocarbon dates (681 radiocarbon dates, or more than 40% of the radiocarbon dates we found; see data S3 and S4). All dates with proven reservoir, weaning, or old wood effects were excluded. Furthermore, we combined multiple radiocarbon dates from one individual into a single date using the R-Combine function in OxCal (*58*). If the X2 test for the R-combine function failed because of a large discrepancy between the two dates, then we chose the more recently analyzed date over the older one. In some instances, multiple radiocarbon dates on charcoal were available from the same structural elements in the burial pit (e.g., burnt planks of a coffin or burial chamber). In these cases, we retained the radiocarbon date with the youngest mean to mitigate potential old-wood effects.

**Fig. 6.**
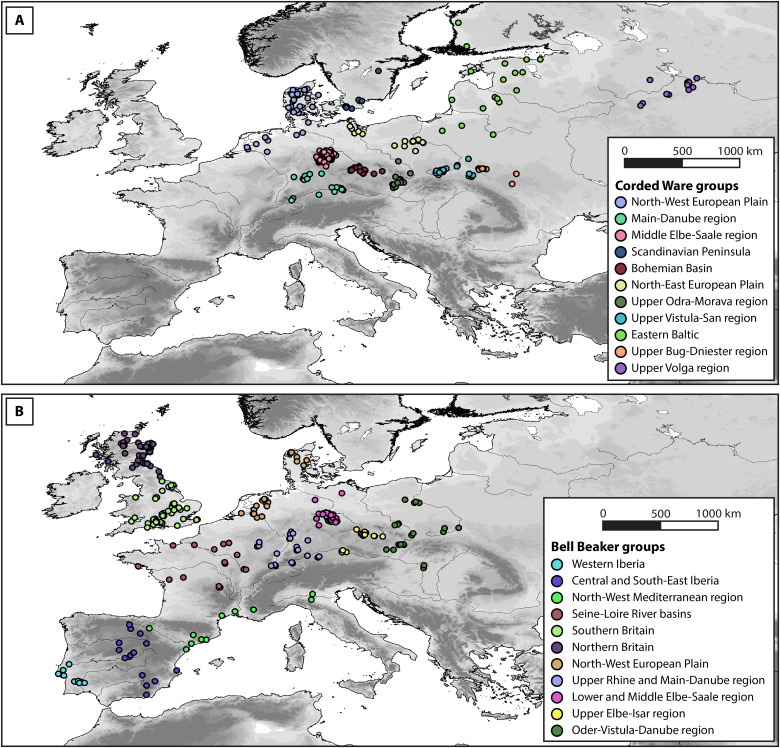
Regional clusters of selected CW and BB radiocarbon-dated burial events. The regional clusters are defined based on general archaeological consensus while ensuring sufficient sample sizes and are described in detail in the supplementary information. CW clusters are shown in panel (**A**), and BB clusters in panel (**B**).

Following this selection process, we grouped all radiocarbon-dated burial events into regional clusters on the basis of archaeological consensus (see supplementary text and Fig. 6) while ensuring sufficient sample sizes in each group to perform further analysis. The grouped dates were imported into OxCal v4.4.4 and calibrated at 95.4% CI with the IntCal20 calibration curve (*58*, *59*). We summarized each group of radiocarbon dates with the KDE_Model function in OxCal. Kernel density models (KDE) provide more accurate summaries of radiocarbon dates than the commonly used summed probability density and summed calibrated probability density models. KDE is more accurate around the edges of the distributions and offsets potential old and young outliers (*29*, *60*). In addition, KDE is less prone to artifacts caused by platforms in radiocarbon calibration curves, although considerations about noise and signal continue to apply (*29*). This procedure resulted in 22 regional KDEs of CW or BB burials. These probability density functions indicate the probability of burial events taking place at a given time interval, based on the temporal distribution of the original radiocarbon-dated burial events. The integrated probability density functions for each regional group are shown in Figs. 1 and 2.

To examine the potential influence of plateaus in the calibration curve on the results, we used the R_Simulate function in OxCal to simulate a radiocarbon dataset with a uniform incidence rate and a temporal span identical to the empirical data. We then calculated a KDE model for these simulated data. Given the uniform incidence rate, any peaks or throughs in probability densities result from the shape of the calibration curve rather than an underlying pattern. The simulation shows only minor effects on the KDE models in our analysis, aligning with other studies (*29*, *61*, *62*) (see supplementary text and fig. S1).

### OLE modeling of “start” and “end” dates

To statistically infer the most probable start and end dates for CW and BB burial rites in each regional group (*n* = 22), we used OLE modeling. OLE is a frequentist modeling approach, originally used in palaeontological and conservation sciences, which has been increasingly applied in archaeological contexts to statistically infer the start (“origination”) and end (“extinction”) dates of cultural and biological phenomena based on sparse and censored chronological data (*30*, *35*, *63*). The formulaic expression of the model is available in existing articles (*30*, *34*, *64*, *65*), and the script used for our analysis is made available here in full (see supplementary text). The script is an adaptation of that used by Djakovic *et al.* (*35*), adjusted to use larger datasets. All OLE modeling was performed in R (version 4.1.1).

We constructed two OLE models for each regional group of BB and CW burials to produce probabilistic estimates for their start and end dates, respectively. The dataset used to construct both the start and end estimates for a group consists of the complete set of (individual) modeled age ranges corresponding to that group, with the exception of statistical outliers showing low agreement within the KDE models (*A* ≤ 60%, *n* = 16; see data S1). Given the error ranges inherent to radiocarbon dating, we chose to use modeled age ranges for the OLE analysis (instead of unmodeled ranges) to produce more conservative, and likely reliable, estimates. To estimate the start date, OLE is run in the reverse temporal direction (i.e., youngest to oldest) while, for the end date, it is run in the forward temporal direction (i.e., oldest to youngest) in both cases using the same dataset (i.e., one dataset per regional group). A complete breakdown of the datasets (*n* = 22) used to produce the OLE models is available in data S1.

To ensure the start/end date estimates are as robust as possible, we used the resampling technique in our OLE modeling (*35*, *63*). A discrete date is randomly drawn from each modeled age range in the dataset and an individual OLE start/end estimate is produced using these randomly drawn dates (i.e., each modeled age range in a dataset is represented by a single random date drawn from its distribution). This process is then repeated for 10,000 iterations (per model), and the aggregated results of these iterations constitute the probability distribution of each start (*n* = 22) and end (*n* = 22) estimate reported here (total, *n* = 44). We consider this approach to be more statistically robust and interpretatively cautious than the alternative (central estimate technique), which uses the mean of each age range as the singular unit of analysis. The integrated probability distributions for the emergence and extinction dates of CW and BB in each region, based on OLE, are shown in Figs. 1 and 2. The mean (OLE) values reported in this article represent the mean value of the 10,000 iterations.

### Construction of interpolation maps

To produce the interpolation maps for the arrival of CW and BB burial rites, we exported all mean modeled dates from OxCal v4.4.4 and combined these data with the geographic locations of each radiocarbon-dated burial events (see data S1 and S2) to create the layer “all points.” The mean modeled date provides the most robust point summary of a radiocarbon date (*66*). We selected the “oldest mean dates” from all points for the interpolation by excluding dates with *A* ≤ 60% in the KDE (*67*), calculating the geodesic distance between any pair of the remaining dates with the geodesic function in the GeoPy (v2.4.1) package with the WGS-84 ellipsoid (*68*), and eliminating all dates which were within 100 km of a date with an older modeled mean (*69*).

We then imported the layers all points and “oldest mean points” into ESRI ArcMap v10.8.2. To approximate the spatial extent of CW and BB, we buffered the points in all points by 150 km and intersected these buffers with a vector map of the European coastline (*70*). Last, we applied an inverse distance weighing (IDW) algorithm with power 3 in ArcMap to interpolate the mean modeled ages of the oldest mean dates with the intersected buffer as input mask. Interpolations with other power values for IDW and with other interpolation algorithms (spline, kriging, and nearest neighbor) were also attempted but returned similar or suboptimal results due to the uneven spatial distribution of the points (see Fig. 6).

Three further geospatial datasets were used as background data for the maps in Fig. 3. These include a digital elevation model (*71*) and two datasets for rivers (*72*, *73*).

### Correspondence of arrival dates for steppe ancestry and CW and BB burials

Predictions for the arrival date of steppe ancestry were obtained by generating “arrival maps” with the spatiotemporal kriging procedure in R as published by Racimo *et al.* (*24*). We input the base data from Racimo *et al.* (*24*) and Allentoft *et al.* (*5*), separately, together with spatial grids of 1000 and 2000 points, respectively. Given the different spatial extent of the interpolation areas in these studies, this number of points amounts to grid cells with comparable sizes (the length of the sides is ~70 to 80 km). We used time slices at 50-year intervals to match the spatiotemporal resolution of the interpolation surface in Fig. 3. The ancestry cutoff was set to 0.5 for the ancestries “YAM” in the study by Racimo *et al.* (*24*) and the major steppe ancestry component “MiddleDon_7500BP” in the study by Allentoft *et al.* (*5*) as they observe that this results in the most accurate arrival dates for steppe ancestry.

The resulting points with the predicted arrival date of steppe ancestry from both models were imported into the ArcMap environment with the interpolation surfaces for the arrival of CW and BB burial rites (see Fig. 3). The predicted arrival date of CW and BB burial rites at the respective location of each point was then appended to these points. Last, all points outside our interpolation surfaces or with null values for the predicted arrival date of steppe ancestry were discarded before comparison.

The Kendall’s Tau-b measure was then calculated with the Python package SciPy (v1.14.0) (*74*–*77*). This metric was selected because the arrival dates are ordinal variables (years BCE for burial rites, and 50-year windows in years before the present for steppe ancestry) with several ties due to the 50-year chronological resolution. An additional advantage of looking at the rank order of predicted arrival dates rather than the absolute values for the arrival dates is that minor differences in predictions due to different treatment of radiocarbon dates do not affect the outcome.

To clarify the outcome of the Kendall’s Taub-b test, we drew a transect across the CW and BB interpolation surfaces in Fig. 3, mapping the value of the interpolation surface at intervals of 100 km, including the terminal points of the transects. We performed the same operation on the points from Allentoft *et al.* (*5*) and Racimo *et al.* (*24*), mapping the values of the geodesically closest points to the points on the transect. We then appended the age ranks of each point in the respective dataset to the transects. The resulting differences in age rank and absolute age for each point along the transect relative to longitude are shown in Fig. 4.

## Supplementary Materials

### The PDF file includes:

Supplementary Text Fig. S1 Legends for data S1 to S8 References
