# Supplementary material for: Spatiotemporal reconstruction of Corded Ware and Bell Beaker burial rituals reveals complex dynamics divergent from steppe ancestry
Source: Sci Adv. 2025 Aug 20;11(34):eadx2262. doi: 10.1126/sciadv.adx2262 (PMC13155569; doi:10.1126/sciadv.adx2262)
Supplement: Supplementary file 1 — Supplementary Text Fig. S1 Legends for data S1 to S8 References [file sciadv.adx2262_sm.pdf]

Science Advances
AAAS

Supplementary Materials for

Spatiotemporal reconstruction of Corded Ware and Bell Beaker burial rituals reveals complex dynamics divergent from steppe ancestry

Quentin P. J. Bourgeois *et al.*

Corresponding author: Quentin P. J. Bourgeois, q.p.j.bourgeois@arch.leidenuniv.nl

Sci. Adv. **11**, eadx2262 (2025)
DOI: 10.1126/sciadv.adx2262

The PDF file includes:

Supplementary Text
Fig. S1
Legends for data S1 to S8
References

Other Supplementary Material for this manuscript includes the following:

Data S1 to S8

Supplementary Text

Regional Cluster Overview

Authors:

Florian Helmecke & S. Louise Olerud*

*Faculty of Archaeology, Leiden University, Einsteinweg 2, 2333CC Leiden, the Netherlands

For this analysis, we compiled a dataset of 967 radiocarbon-dated burial events, which were grouped into regional clusters with robust sample sizes. Only dates deriving from graves unambiguously associated with Corded Ware (n = 453) or Bell Beaker (n = 514) burial practices were included. This approach yielded 11 distinct Corded Ware clusters and 11 Bell Beaker clusters (see Extended Figures 1 and 2), which are discussed in more detail below.

1. Corded Ware

Cluster 1 (Upper Volga region), situated in the wider Upper Volga region comprises 29 graves from 13 sites of the Russian "Fatyanovo" culture, which is considered the most eastern branch of the wider Corded Ware complex (*78*). The sites included in our analysis are situated in a corridor roughly delineated by the city of Tver in the north-west, Moscow in the south-west, the Rybinsk Reservoir in the north-east and Iwanowo in the south-east and show a distribution which mainly follows the catchment area of the Upper Volga River and its tributaries in the East European Plain. According to the (typo-chronology-based) Fatyanovo subgroup nomenclature by D.A. Kraynov, the cluster collates burials of the so-called Moscow-Klyazma- and Upper Volga Fatyanovo groups (*79, 80*), which represent core areas of the wider Fatyanovo distribution (*80*). Altogether, 29 burial events were considered for the analysis, with almost all samples taken on human bone material. Only in the case of grave 5 at Kuzminki (*81*), charcoal attached to a copper ring was dated (*82*). Aside from that, seven dates from six sites had to be excluded due to the developed criteria.

In principle, burials constitute the main source of information for the Fatyanovo group of Corded Ware and indicate the core area of its distribution. The majority of herein considered dates derive from burials discovered in the area between Yaroslawl and Iwanowo, often situated on moraine hills or high slopes of river or lake valleys (*83*). Typically, the dead were placed in flat graves, buried in crouched body positions, with men mostly placed on the right side and women on the left side. Individuals were mostly buried along the (north)east–(south)west axis, both facing southward. But also deviations from the typical orientation (e.g. at Grave 22a from Volosovo-Danilovskiy (*84*)) or gender-differentiated norms in body positioning (e.g. at Naumovskoye (*79*)) are known. Nevertheless, they rank low in number. The bulk of data consists of single inhumations, whereas also two multiple burials rank among the dated

graves. Notable is the grave of Nikolo-Perevoz (*85*), in which nine individuals featured in one burial pit.

Fatyanovo burials typically represent rectangular flat graves with rounded or protruding corners. The grave walls and floor are regularly covered with organic materials such as light constructions made from wooden logs, bast, bark, braids of osier - and perhaps even animal skins (see e.g. burials of the Volosovo-Danilovskiy burial ground (*84*); or the 'wrapping' in Naumovskoye, grave 2 (*80, 86*)). Further there is occasional evidence for lighting fires in the grave pit and/or subsequent firing above the grave (e.g., in Kuzminki grave 5) (*78, 79, 81*).

In many burials the dead are equipped with wedge-shaped flint axes and pottery vessels. Among the latter, especially beaker, amphorae, pots and bowls with a globular body and either S-shaped profile or straight-walled neck are known. Often, the upper part of the body is decorated with horizontal rows of stamps, geometric motifs or fish-bone patterns. Also, stone battle-axes are known, but more likely to be found in male than female graves, which equally applies to whetstones. Other typical grave goods are represented by a range of flint tools (blades, scrapers, points), among which also arrow- and spearheads feature (see e.g. Nikolo-Perevoz, or Khanevo, grave 4). Further, bone and antler tools (awls, chisels, points, spatulas) are found frequently. Ornaments made of animal teeth, bone, amber or small shells and mostly in the form of pendants are predominantly found in female graves. Moreover, bone pins and copper ornaments are known. Additionally, unworked bones of pigs or sheep/goats, at times placed in pottery vessels, often feature in burials and are conceived as food offers or meal leftovers (*78, 79*).

Chronologically, Kraynov's group division model with temporal implications was held up for a long time, proposing temporal differences among the Fatyanovo subgroups in a west-to-east cline. The arguments therefore derived from typochronologies, analogies, and stratigraphy, while radiocarbon dates played little to no role. For the cluster region, it was previously proposed that the Moscow-Klyazma group represented an early Fatyanovo stage, while the Upper-Volga group was considered a developed stage, before later Balanovo-Fatyanovo groups established further east (*78, 80*). Our analysis objects to these inferences, which were largely built on typological arguments. Nonetheless, the absolute chronology of the Fatyanovo culture is relatively meagre and was only recently enhanced by a study of Saag *et al.* (*87*), which provided 26 new 14-C measurements. By now, the radiocarbon dates are still limited to only 35 grave contexts which showcase a bimodal distribution along the calibration curve.

Our findings refine Krenke's (*82*) proposed absolute chronology for Fatyanovo between 2750–2500/2300 cal BC and suggest the earliest possible introduction of Corded Ware-fashioned (= Fatyanovo) burials from c. 2758 cal BC onwards. Moreover, the KDE's indicate the highest likelihood of most Fatyanovo burials to date into a ~155-year period between c. 2595–2440 cal BC. This finding suggests that the introduction of the Corded Ware burial practice in the Upper Volga region occurred slightly earlier than in the Upper Bug-Dniester region (cluster 3), but more or less simultaneously with the introduction of Corded Ware burial rites in other eastern provinces, such as the Upper Odra-Morava and Upper Vistula-San regions (cluster 4 and 5). Moreover, this probability peak aligns well with the second main peak observed in the KDE model of the adjacent Eastern Baltic region (see cluster 2). After this period, the probability for burial events of younger age strongly decreases until the time

between c. 2200–2045 cal BC, when a meagre second peak in the KDEs signals a last probable 'revival' of the Corded Ware burial custom before it vanishes entirely after approximately 2034 cal BC, according to the OLE. While Krenke raised legitimate doubts on the validitiy of the few later Fatyanovo dates from post-2300 BCE (*82*), there continues to be subtle evidence for later Fatyanovo sites (see e.g. the burial sites Miloslavka, Turginovsky, Volosovo-Danilovskiy or the RANIS floodplain settlement site), whose late dates were measured by different labs.

Of the 29 radiocarbon dated burials in the cluster, 27 underwent further archaeogenetic analysis (*87, 88*). Both studies revealed that Fatyanovo individuals shared no ancestry with preceding hunter-gatherer populations (associated with the Volosovo culture in the region), signaling the former as being 'newcomers' to the region. In fact, bearers of the Fatyanovo culture revealed a great genetic affinity to Corded Ware-associated populations in the Baltic, Central Europe and southern Scandinavia, as recently further underlined by the detection of high amounts of shared ancient IBD segments among these groups (*89*). Moreover, all analysed males in both studies carried Y-chromosomal DNA haplogroups R1a-M417 (*87*)(or subclade R1a-M93 (*88*), which only appeared in Europe after the steppe migration (*2, 3*). This hitherto observed homogeneity of Y-chromosome haplogroups is associated with founder effects and genetic drift within an isolated ancient migrant group (*88*), supporting the proposition of an eastward migration of Central European Corded Ware group(s) in the formation process of Fatyanovo. At the same time, the genetic make-up of Fatyanovo individuals revealed a mixture of steppe-related ancestry and Anatolian Early Farmer ancestry, implying that a direct migration of Yamnaya-associated individuals (who have no Early Farmer ancestry) from the Pontic Caspian steppe into the East European Plain is ineligible (*87, 88*). While the study by Saag *et al.* foreshadowed that either Trypillia or Globular Amphora culture populations could represent likely proxies accounting for the Early Farmer ancestry in Fatyanovo groups (*87*), this was later verified by Ringbauer *et al.* (*89*) who detected that Fatyanovo individuals shared long IBDs with Globular Amphora-associated populations in Poland and Ukraine.

Cluster 2 (Eastern Baltic) encompasses samples from both coastal and inland sites of the wider Eastern Baltic Sea region. The cluster area stretches from the west Finnish Ostrobothnia region in the north to the Masurian lake-district in the northern Polish Lowlands and the Neman valley in the Grodno region of modern-day Belarus to the south. It includes samples from different Corded Ware provinces in the area, which were traditionally distinguished as: *Baltic Corded Ware* in the Lithuanian inland, Latvia, and Estonia (*90–93*), *Finnish Corded Ware* in Finland (*94*); and the *Rzucewo culture* (also known as *Bay-Coast-, Baltic Coastal-* or *Haffküsten culture*) in coastal Lithuania, the Kaliningrad Region, and Poland (*95, 96*).

Our dataset includes 22 burial events from 18 sites, with most samples derived from direct human bone samples. To minimise the risk of potentially underlying aquatic reservoir effects in samples (*97, 98*), individuals with signals for terrestrial diets (n = 6) or samples from animal bones, for which aquatic reservoir offsets are ineligible (n = 2), were preferred wherever possible (*41, 98, 99*). In ten cases, the effects of underlying reservoir offsets remain unknown. These dated burial events, however, matched the sample selection criteria and based on recent results Corded Ware individuals in the region mainly had a terrestrial diet (*41*), we do not expect these to be significantly impacted by a reservoir effect. It is noteworthy that the age estimates for these cases fit well with the rest of the data. In the case of grave 2 from Kunila (*41*), an RCombine operation was conducted on the skeletal material of the deceased.

Due to the specifically acidic soils in Finland that allow for almost no bone preservation, the sample from Teuva Kortesnevankorpi was derived from a food crust sample on a grave good vessel (*100*), while the sample from Kukkarkoski represents the only charcoal sample in the cluster dataset (from the floor of the burial pit (*101*)). Further, one sample was taken from a nutshell found inside or around a Corded Ware vessel in Narva-Jõesuu IIB, which can also be associated with the burial event (*102*). Eight dates were excluded due to poor fit with the selection criteria.

Compared to other regions of the wider Corded Ware distribution, the burial record from the Eastern Baltic is relatively scarce, consisting of simple flat earth graves without mound cover. These are known from either solitary locations or small grave groups. Except for the adult-child double-burial from Selgas (*103*), all burials considered in the cluster dataset represent single inhumations. The dead were mostly placed in crouched or supine-crouched positions, largely oriented towards the E-W axis, although deviations occur (*101, 104, 105*). In the case of grave 1 at Sope B, more complex secondary burial rites are evident, attesting multi-staged funerary practices among Corded Ware communities in the Eastern Baltic region, which included wrapping or binding of the cadaver (*106*). Typical grave goods include stone battle-axes, flint axes, flint blades, bone tools and pins, cord-ornamented ceramics, as well as amber and shell ornaments. Worked and unworked animal bones are also regularly identified (*101, 104, 105, 107*).

Previous chronologies based on grave and settlement data determine Corded Ware in the Eastern Baltic between 2900/2700 cal BC and 2400/2300 cal BC (*104, 108, 109*), with a longer fade-out in some regions until c. 2000 cal BC (*105, 110*). Our models suggest the earliest possible appearance of canonical Corded Ware burials in the Eastern Baltic from 2944 cal BC onwards, making it the oldest cluster region to feature Corded Ware burials in Europe, followed by cluster 6 (Bohemian Basin). The probability of burial events shows a first KDE peak between 2900-2750 cal BC and a substantial second peak between c. 2575-2450 cal BC, indicating an early and main phase in the introduction of Corded Ware burial practices for the region. After this, the probability distribution steadily decreases, leading to an estimated discontinuation of the burial custom after 2172 cal BC. An example of a late-stage burial is grave IX-1 from Dudka (*111*) in the Masurian lake district, which already shows changes in burial practices at the end of the third millennium BCE. While this burial represents a left-flexed inhumation in south–north orientation and is equipped with two ceramic beads, it still qualifies as part of the wider Corded Ware complex (*111*), despite showcasing influences from both coastal Subneolithic and Bell Beaker communities in the east.

Of the 22 samples in the cluster, nine individuals were archaeogentically analysed (*11, 12, 87*). Most of those individuals can be dated into the 26th-25th century BCE and all show the 'steppe signal' in their genetic make-up. One older dated sample from Plinkaigalis grave 242 even appeared cladal with Yamnaya-associated individuals together with individual 1 at Gyvakarai (*11*), indicating strong ancestral links between some of the inhabitants of the Eastern Baltic Sea region and steppe populations. Three further individuals (Naakamae1, Plinkaigalis241, Spiginas2; (*11*)) were excluded from the analysis due to uncertainties in Corded Ware ascription.

Cluster 3 (Upper Bug-Dniester region) represents the area closest to the European steppe region, extending from the west Ukrainian Yampil region in Podolia to the Sokal Ridge in the Volhynian Uplands of modern-day south-eastern Poland. This region is broadly situated around the Upper (Western) Bug and Upper Dniester River systems. Previous research has ascribed Corded Ware communities of this area to the local Lubaczów-Upper Dniester group of Corded Ware, contrasting them with the more westerly Craków-Sandomierz group found between the Upper Vistula and San River (*112, 113*). It is noteworthy, however, that Corded Ware communities along the San River were excluded here and clustered with burials from the Upper Vistula region (cluster 4), due to their closer archaeological affinity with the Craków-Sandomierz group (e.g., sharing the custom of niche-flat grave construction) than with the barrow graves of the Sokal Ridge (also called the 'Sokal group' (*114*)). This decision was further supported by the close connection of the San River to the Vistula River system, enabling an east–west gradation in the spatiotemporal analysis to test the cluster regions' bridging function between Central Europe and the East European Forest steppe.

A total of 20 burial events from 14 sites were included in the analysis, while three dates were excluded due to poor fit with the established criteria. The majority of samples derive from human bone material, with only two charcoal samples from verified direct context associations included (Úlow 3 (*115*) and Lipie (*116*)). One excluded burial event was burial 9 from Hubinek 2. Despite its very early date indicated by the published calibrated range (Poz-70675: 3025-2898 BC (*36*)), its actual BP age and standard deviation remain unpublished. While mentioned as a Corded Ware grave in Juras *et al.* (*36*), there is no scientific consensus about its ascription to the Corded Ware horizon, with more indications for an ascription to Pre-Yamna or Yamnaya funerary traditions predating Corded Ware. Nevertheless, the early barrow undisputably displays cultural features known from other steppe barrows (*116, 117*). The barrow grave from Siwki (*118*) was included in our KDE model, but exhibited a poor agreement just below the threshold of $A \leq 60$. Therefore, it had no effect on the interpolation surface in figure 1.

The graves in this cluster are exclusively linked to barrows, a characteristic of Corded Ware burials in this region. Most data derive from barrow graves at the Sokal Ridge (western Volhynian Uplands), forming a ~30 km long barrow alignment along an E-W axis. In this alignment, barrows are usually grouped in small concentrations of 2-3 mounds, with distances of 100-500 m between each group (*119*). In the cluster group, graves typically contained single inhumations, with a few double graves known (e.g., an adult-child grave in barrow 1 at Wierszczyca 29, an adult-adult grave in barrow 1, grave 2, at Hubinek 4, and a child-child grave at Łubcze 25, barrow 2, grave 3/W; (*119*)). Single inhumations in rectangular or oval grave pits were broadly oriented along the E-W axis, with males often placed in right-flexed positions with heads towards the west. Secondary grave interments at the barrow parameters represent simple pit- or niche graves (see e.g. grave 2 in barrow 2 at Łubcze 2 (*119*). Specifically the latter, niche graves, must be highlighted as a local idiosyncrasy that is largely confined to Corded Ware communities in south-eastern Poland and has high concentrations along the left bank of the Upper Vistula (see cluster 4; (*120*)). They represent subterranean chambers for the deceased, which were entered through a slightly higher situated entrance pit, commonly found on the eastern or southern sides of the niche constructions. In the cluster region, they were commonly interred as secondary graves into (the perimeters of) barrows, which is why their general orientation is not uniform but slightly more variable (*119*).

Typical grave goods include amphorae, beakers and cups, as well as flat axes, blades and scrapers made from flint. Stone battle-axes and flint arrowheads are found regularly in central barrow graves of

anthropologically determined males and also bone chisels and awls made from animal bone feature frequently in burials. Rarely, graves included copper, bone, or amber ornaments (*118, 119*). Noteworthy is the observation that parts of the pottery assemblages from burials of the Sokal Ridge displayed strong links to the Middle Dnieper Culture (MDC). These are, however, exclusively found in stratigraphically younger graves (*119, 121*).

Radiometric measurements obtained from graves of the Sokal ridge (*122*) represent the second largest series of radiocarbon dates from Lesser Poland (*123*). Initially some of these graves were dated into the period between 2800-2600 cal BC while their assemblages, funerary practices, and stratigraphic context indicated a much younger Corded Ware dating, similar to the majority of graves radiocarbon dated to the mid-third millennium BC (see e.g. old measurement from secondary niche grave 2 in barrow 2 at Łubcze 2 [Ki-6297]; (*116*)). Recently old radiocarbon dates have been redated with AMS radiocarbon dates on human bone samples from these contexts (*116, 124*). They confirmed that some disputed dates obtained from the Kyiv laboratory can be considered outliers (*125, 126*). Therefore, older dates from the Kyiv laboratory were excluded in favor of more recent dates from the Poznan laboratory. Only in the case of grave 2 from barrow 2 in Nedeżów 22 a RCombine operation was conducted successfully in OxCal version 4.4.

Chronologically, our analysis aligns with the latest KDE_SUM models proposed by Jarosz & Włodarczak (*116*) for the Sokal Ridge and adds estimates for the first and last instances of canonical Corded Ware burials in the cluster region. According to our OLE model, the earliest Corded Ware in the cluster region can be expected from c. 2679 cal BC onwards, making it the latest region across the wider Corded Ware complex to exhibit typical funerary customs like barrow graves with distinct Corded Ware assemblages. This is striking in view of the highest possible proximity to the region in which the genetic admixture between genetically ancestral Yamnaya and Globular Amphora communities potentially manifested during the second half of the 30th century BCE (*127*). Considering the overall distribution of dates in the KDE model, the highest KDE probability of Corded Ware burial events is found for the period between 2575 cal BC and 2300 cal BC, which, compared to other regions, reveals a more spread-out probability concentration than e.g. in adjacent cluster 3. This notable extended distribution is interesting in light of the two funerary traditions which co-occurred for a while in the cluster region. While initially only barrow-building was characteristic, a subsequent niche construction phase seems to have emerged from around 2470 cal BC onwards (*116*). Our model suggests that after c. 2216 cal BC the Corded Ware burial custom was discontinued, aligning with the proposed end of the younger Corded Ware niche burial practice around 2270 cal BC (*116*).

Of the included burial events in our analysis, two individuals (pcw350 and pcw 362) underwent further archaeogenetical analysis in Linderholm *et al.* (*124*). According to Linderholm *et al.*(*124*), these individuals shared ancestry with steppe populations but also had strong connections to individuals in Central Germany and the German lowlands.

Cluster 4 (Upper Vistula-San region) encompasses burial sites from an area that is largely drained by rivers linked to the Upper Vistula, including the San River and an area further north-west in the Lublin Uplands. Most sites of the cluster are situated in the loess areas of the Małopolska and Sandomierz Uplands, the Carpathian foothills, as well as the Subcarpathian part of the Sandomierz

basin (including the Rzeszów foothills). These sites represent one of the Corded Ware regions with a rich burial and radiocarbon data record. Corded Ware communities in the cluster area are traditionally ascribed to the regional Craców-Sandomierz group of Corded Ware (*112*, *128*, *129*), whose sites are generally situated on loess patches or directly adjacent zones. They often occupy elevated parts in marginal areas of plateaus, which are in close proximity to larger rivers, or are situated in their valleys (*129*, *130*). As mentioned in cluster 3, sites located near the San River (between Przeworsk and Przemysl), traditionally ascribed to the Lubaczów-Upper Dniester group of Corded Ware, were incorporated into this cluster due to the strong similarity of burial customs in the considered graves and the geographical connection of the San to the Upper Vistula network.

Burials in the cluster area are evident from an array of sites, including isolated locations, small grave groups, or cemeteries containing up to 64 Corded Ware burials (e.g., Żerniki Górne 1, from which the largest radiocarbon date series for a Corded Ware site in Lesser Poland was obtained (*131*)). Due to an extensive history of nearly four decades of small- to large-scale dating programs within the cluster region (*116*, *118*, *123*, *132*, *133*), it represents the second largest source for Corded Ware radiocarbon dates in our analysis. In total, 78 burial events were modelled, of which the vast majority derives from human bone samples. For the well discussed example of grave 3 at Zielona 3 (*126*, *134*) (no RCombine function was feasible due to the enormous temporal distance between both dates from the same individual. Therefore, the more recently dated sample (Poz-9585) was considered. Furthermore, due to poor skeletal preservation at Wilczyce 10 (*135*), an animal bone sample was taken from an accompanying dog in the grave pit. In two other cases charcoal samples with direct links to the grave pit were included. While the sample from the central grave in barrow 1 at Średnia 3 (*136*) yielded a very old date, it has been previously suggested that the measurement was potentially impacted by an 'old-' or 'heartwood effect' affecting the accuracy of the sample (*28*). In our KDE model it was identified as outlier and thus irrelevant for the interpolation surface in figure 1, as it exhibited a model agreement significantly below the threshold of $A \leq 60$ (see methods). Moreover, 20 dates associated with 18 burial events (including almost all samples from the Carpathian foothills; (*116*) were excluded since they did not match the applied criteria. This includes the early sample from Szczytna 6 (*137*), which had no direct association with the burial event and is suspected to be affected by a dating error (*125*). Similarly, samples from the barrows in Hancovke (*138*, *139*) and Bierowka (*132*, *140*) were left out as they originated from outside the actual grave pit and could not be directly linked with the attested Corded Ware burial event. While these dates frequently feature in chronological models of Corded Ware burial rites, we advocate for their rejection in models specifically aimed at dating Corded Ware burial events. This recommendation is based on the lack of direct associations between the dates and the actual burial events, which still remain to be radiocarbon dated.

In the cluster region, the predominant burial type is niche-flat graves, though barrows are also present, making up about 15.38% of our cluster dataset. The deceased in central barrow graves were generally buried in a binary gender-differentiated and bipolar manner, in crouched positions along the west–east axis, with males oriented westwards and facing south (e.g., grave 1 in Gabultow 1 (*118*)). In contrast, deceased in barrow niche graves typically followed a north–south axis orientation (e.g., Koniusza 2 (*133*)). This north(north-east)–south(south-west) axis orientation is also prevalent in niche-flat graves, which constitute the bulk of the data. Individuals in these graves were often placed in supine-flexed positions, facing eastward towards the niche entrance and equipped with various objects typically placed behind the individual's back. Characteristic grave goods are vessels like amphorae, beakers and jugs. Lithic tools include stone battle-axes, flint tools such as flat axes, arrowheads, (retouched) blades, scrapers and flakes as well as sandstone whetstone. Further there are many finds of bone and antler

tools such as chisels, wedges, awls and batons. The range of ornaments encompasses bone, antler or shell beads, spiral copper rings. While it appears that the general body placement principle involves burying males on the right side and females on the left side, there is also a group of men to be distinguished, which were buried on their left side. The reversed situation of a woman positioned on the right side is however absent (*129*).

While the majority of graves represent single inhumations, there are also numerous non-single graves present. These typically represent double graves, with occasional instances of three individuals sharing a niche (see e.g. grave 2 at Gabultow 1 (*118*)). In our cluster dataset, these double and multiple graves constitute ~20%, aligning with patterns observed at large cemetery sites like Żerniki Górne 1 (*131*), where ~23% of the graves represent non-single inhumations.

Given the substantial amount of available radiocarbon dates, the cluster region offers a good opportunity to estimate the absolute chronology of Corded Ware. Recently, Jarosz & Włodarczak (*116*) modelled the presence of Corded Ware graves from the very beginning of the third-millennium BCE (in the Carpathian foothills) up until 2346-2292 cal BC (in the Rzeszów foothills), while the Małopolska Uplands featured Corded Ware barrows between c. 2800-2450 BCE and niche graves in the period between c. 2500-2300/2250 BCE (*116*). According to our models, in which many early graves from the Carpathian foothills were excluded due to indirect sample-context associations, we see the earliest potential emergence of the Corded Ware burial rite in the region from c. 2751 cal BC onwards (OLE). Nevertheless, the unimodal distribution of dates in the KDE model indicates a clearly delineated probability peak for the interval between 2500-2300 cal BC, while the estimated disappearance of the Corded Ware burial custom according to the OLE model manifests shortly after, around c. 2318 cal BC. This indicates that with a high probability the majority of burial events occurred within a period of roughly 200 years.

From the substantial burial record of the cluster region, seven individuals underwent further archaeogenetic examination in Juras *et al.* (*36*) and Linderholm et al. (*124*). Similarly to individuals from cluster 3, ancestry sharing with steppe populations but also strong connections to individuals in Central Germany and the German lowlands were detected.

Cluster 5 (Upper Odra-Morava region) encompasses the historical region of Moravia and extends along the Upper Odra into Upper and Lower Silesia. Corded Ware in the Moravian part of the cluster region is traditionally labelled *Moravian Corded Ware* in order to differentiate it from its Bohemian counterpart and to highlight its strong links to the Carpathian-Balkan cultural sphere (*141–143*). At the same time, Corded Ware communities along the Upper Odra are commonly studied from a Polish perspective and compared with other Polish variants of Corded Ware (*112, 144*). Due to the spatial proximity of some Polish sites to the Moravian Gate and their location along the Upper Odra, these sites were merged into the Moravian cluster.

A total of 40 burial events from 21 sites were considered for the analysis. Most sites are situated within the wider Morava drainage basin, while two sites are situated along the Upper Odra river system in Silesia. The majority of samples derive from human bone material, and only in six instances were

animal bones with direct context association sampled. An RCombine operation was performed for the radiometric measurements from Vyškov-Dědice, Legerní pole (*143*). For feature 780 at Pavlov, Horní pole and grave 19 at Olomouc-Slavonín 1, a poor model agreement (A ≤ 60, see methods) of the radiocarbon dates in the model was observed, having no effect on the rendering of the interpolation surface in figure 1. Additionally, 16 burial events were excluded from the overall analysis based on established criteria, including grave 17 at Olomouc-Řepčín 1, which was, however, excluded due to an insufficient collagen content in the human bone sample (*145*). Several burial events had to be excluded because of a lack of published context data, highlighting the need for these burial contexts to be made available for future chronological modelling.

Corded Ware in the cluster region is primarily known from burial contexts or isolated finds. Although the number of burials is lower than in the Bohemian basin (cluster 6), the burial practices are similarly characterised by single inhumations, either in solitary graves or small cemeteries of up to 40 burials. Usually, the graves are at a greater distance from each other, which is why they are considered as being originally mound-covered. However, current implications suggest that barrow graves seemed to have been less common than flat graves for single individuals (*141, 143*).

In the cluster dataset, there are also three non-single graves, which in the case of grave 67b at Olomouc-Slavonín 1 contained up to three left-flexed individuals (*146*). While grave 18 at Olomouc-Nemilany 3 represents a Corded Ware typical double-inhumation burial (*145*), feature 780 at Pavlov, Horní pole stands out for containing the cremated remains of two individuals (*147*). Although rare, the cremation burial rite is also documented in grave 4 at Držovice-Pastviska (*148*), indicating that cremation was part of the Corded Ware funerary repertoire in Moravia albeit playing a minor role (*141*).

In view of the common inhumation burial make-up, the orientation of the deceased is generally variable, with right-flexed males more often aligned with their heads toward the south and left-flexed females toward the north. This variability in body positioning creates a less pronounced but still recognisable gender differentiation (*141, 149*). Typical grave goods consist primarily of ceramics, including cord-ornamented (handled-) beakers, amphorae, jugs, bowls, and egg-shaped pots. Non-ceramic artefacts include facetted battle-axes, stone flat axes, flint tools (blades, flakes, scrapers), and bone tools such as awls and chisels. Occasionally, also knives and awls made of copper are found, such as in grave 5 at Pavlov, Horní pole (*147*). Ornaments, though rare, include perforated shells, animal teeth, and small copper spirals and rings. Animal bones, particularly of pigs, sheep/goats, and cattle, frequently appear as potential food offerings in graves (*141*).

Chronological models have suggested that Corded Ware in Moravia began later than in Bohemia (cluster 6), where some of the earliest Corded Ware burials are found (*150*). Recent models place the emergence of Corded Ware in Moravia around 2700/2600 cal BC, with an end between 2400/2200 cal BC (Peška, 2023b, p. 147). Other models propose an earlier emergence around 2500 cal BC, with the end extending post-1930 cal BC (*151*). Our OLE model suggests that Corded Ware burial customs emerged around 2746 cal BC, with a main probability peak in the bimodal distribution of the KDE model between 2600 and 2400 cal BC. This mirrors a very similar development as in other clusters, such as the Upper Volga region (cluster 1) or the neighbouring Upper Vistula-San region (cluster 4). Notable for the Upper Odra-Morava region, however, is the long fade-out of the probability distribution, which after around 2300 cal BC still continues deep into the regional Bronze Age period. Our OLE

model suggests a very late discontinuance of the Corded Ware burial rite, around 1867 cal BC, marking the Upper Odra-Morava region as the last region in the dataset in which local Corded Ware practices were maintained.

Further noteworthy are the genetical analyses of three individuals from the cluster dataset in previous studies (*36, 152*). A simple haplogroup analysis was conducted for the male individual from Jagodno (*152*), while two individuals from Držovice-Pastviska were studied for their mitochondrial genomes (*36*). By examining their genetic distance to other ancient populations, it was demonstrated that the latter exhibited a greater maternal genetic affinity to eastern European steppe populations, such as Yamnaya, Srubnaya, and western Scythians, thus indicating that also females with 'steppe ancestry' contributed to the formation of populations associated with the eastern Corded Ware culture (*36*).

Cluster 6 (Bohemian Basin) encompasses burials from an area stretching from the Bohemian part of the Ore mountains in the west to the Bohemian Plateau and the Polabí region of central Bohemia, extending eastward to the East Elbe Table. Many radiocarbon dates however derive from sites situated in a specific area of the North Bohemian basin, the so-called 'Most basin'. A total of 46 burial events were considered for the cluster analysis, with radiometric measurements predominantly conducted on human bone samples. In two instances (grave 1 at Obříství and feature 79 at Nezabylice) artefacts from animal bones were selected to bypass potential aquatic reservoir effect underlying the human bone samples. While a FRE was detected in Nezabylice, a similar effect is expected for Obříství (*13*). Additionally, three RCombine operations were performed on burials from Plotiště nad Labem (grave LX) and Vliněves (graves 965 and 9566A). Twelve burial events were excluded from the analysis due to a lack of grave goods and failure to meet the established criteria.

In the cluster region the primary evidence for Corded Ware derives from single inhumation burials of crouched individuals, typically oriented along the east–west axis. These burials generally represent flat graves, which often are found in clusters on – at times large – cemeteries, mostly located on elevations in the proximity to streams (*142*). The dataset for cluster 6, however, also includes a few non-single graves. Three of these represent adult-child co-burials, while grave 1/95 from Slany 1 contained (at least) three individuals (*28*).

Based on anthropological and/or genetic sex determinations a relatively clear gender differentiation in orienting the dead is notable, with males being buried right-flexed in west–east orientation and females being buried left-flexed in east–west orientation (*149, 150*). The grave good spectrum of characteristic ceramics includes cord-ornamented (handled-) beakers, amphorae, jugs and bowls. Typical non-ceramic artefacts are (facetted) battle-axes, mace-heads, various forms of flint industry (predominantly flint blades), flat axes and sandstone whetstones, as well as bone tools such as chisels and awls. Ornaments like shell discs, copper ornaments, perforated animal teeth or their bone imitations are generally more common finds in left-flexed burials. Relatively seldom but noteworthy are bone pins and so-called belt plates made from bone and antler (*142*). The latter, though present in areas beyond the cluster region (e.g., in cluster 2 'Eastern Baltic' or cluster 7 'North-East European Plain'), show a concentration in Bohemia (*153*) and display remarkable similarity to each other. The fact that they appear around the same time horizon suggests the existence of shared funerary 'dress' items across wider regions in Europe (*150*).

In the last decade, new radiocarbon dating efforts (e.g., Vliněves (*154*)) and an aDNA transect study in Bohemia (*13*) have significantly expanded the number of available dates from Corded Ware burials. Recent chronological models place the beginning of the Corded Ware culture at around 2900 cal BC, contemporaneous with the local post-Baden-Řivnáč and Globular Amphora communities (*150*). Our analysis confirms that the Bohemian basin represents the oldest Corded Ware cluster, together with the Eastern Baltic region (cluster 2). According to our OLE models, the emergence of Corded Ware typical burial customs can be expected from c. 2939 cal BC onwards. The bimodal KDE model distribution suggests a steep probability increase between 2925 cal BC and 2750 cal BC, followed by a subsequent probability decline. Only from c. 2600 cal BC onwards probability increases sharply, leading to a second KDE peak between 2600 cal BC and 2350 cal BC. This is followed by quick decline, proposing the end of Corded Ware around 2306 cal BC, according to our OLE model.

Of the burial events considered in our analysis, 24 underwent further archaeogenetic investigation (*13, 155*). Their genomic analysis revealed an ancestry make-up of eastern populations mixing with local populations. Especially the study by Papac *et al.* (*13*) demonstrated that the earliest Corded Ware individuals had distinct genetic profiles, whose admixture cannot be modelled without a small but significant 5-15% ancestry from a Latvia_MN/Ukraine_Neolithic/PittedWare-like source. This make-up is distinct from Yamnaya and Central European Neolithic groups and suggests allele sharing with ancient north-east Europeans that persisted into later Corded Ware populations of Central Europe. Equally significant was the finding that four out of four early Corded Ware females lacked any 'steppe ancestry', thus suggesting a processes of cultural assimilation of females, that were genetically close to Globular Amphora-associated individuals. The study also observed a sharp decline in Y-chromosomal diversity over time, resulting in one dominant single male lineage (R1a-M417(xZ645)) steadily supplanting all others. This might have been driven by non-random socio-cultural selection processes or an influx of nonlocal R1a-M417(xZ645) lineages which drove the decline in Y-chromosomal diversity of later Corded Ware groups in the Bohemian basin. Interestingly, the initial genetic diversity of Corded Ware groups did not appear to have influenced burial practices. For the Corded Ware cemetery Vliněves, it has been demonstrated that an apparently culturally 'homogeneous' early Corded Ware burial community was marked by strong genetic diversity. The different genetic make-ups, however, seemed to have played no role at all when practicing typical Corded Ware burial customs (*13*).

Cluster 7 (North-East European Plain) encompasses burials from an area stretching from the north-east German Lowlands of Western Pomerania and the Uckermark to the Polish lowlands of Greater Poland and Kujavia, all the way to the Lower Vistula in the east. Depending on the region, Corded Ware communities in the cluster area are also referred to as '*Einzelgrabkultur*' (*156*) or '*Oderschnurkeramik*' (Corded Ware communities near the river Oder (*157*)). Due to similar tendencies in the stereotypical Corded Ware burial practices across the north-east German and Polish Lowlands, both regions, situated within the Central European Plain, have been grouped for the analysis (*156, 158, 159*).

In the cluster region, diverse burial customs can be identified in graves associated with Corded Ware. A peculiarity of the region, especially notable in north-eastern Germany, is the practice of burying the

deceased in existing megalithic monuments. In Mecklenburg-Vorpommern, for example, almost 50% of all Corded Ware-associated burials represent secondary interments within megalithic structures (*156*, *160*). This approach to burial is distinct from other regions. However, due to the generally unknown association of the samples with secondary Corded Ware 'intrusions' in megaliths, such graves were excluded from our dataset. Non-megalithic graves in the cluster region are also evident and typically consist of simple flat or barrow graves, predominantly found as isolates or in small groups. These graves occasionally include stone packages or frames, likely used to stabilise wooden coffins (*156*, *161*). A few cremation graves are also known, such as the one at Przespolew (*161*), which contained typical Corded Ware grave goods.

The remainder of graves in the cluster region are single inhumations, where the orientation of the deceased is largely gender-differentiated along the north–south axis. Males are often buried in a right-flexed position oriented south–north, while females are oriented north–south, both facing eastward (*149*, *156*, *161*). Typical grave goods include ceramic vessels such as beakers, cups, and amphorae, alongside lithic artefacts like stone battle axes, flint axes, arrowheads, blades, and flakes. Ornaments are represented by beads made from bone or amber, while only rarely bone or antler 'belt' plates or copper ornaments feature. Notably, stone axes and amphorae are predominantly found in male graves, while copper items are absent and only appear in the graves of sub-adults and women (*161*). Further, also in this cluster area, the practice of giving parts – or entire carcasses – of animals (pigs and cattle) is observed (*162*).

In addition to these typical non-megalithic single inhumation graves, the cluster region also contains non-single graves, further illustrating the diversity of ritual practices within the region (*162*). Grave 5 at Pikutkowo 6 is particularly noteworthy, where fourteen individuals were arranged in four opposing groups within the same grave pit, representing a single burial event according to the excavator (*163*). No other Corded Ware-associated grave in Europe has been found to contain such a large number of individuals thus far. Completely different, however, is the situation in two other non-single graves. For example, at Zarrenthin (*164*), only the isolated skull of a second adult individual was found near the feet of the primary Corded Ware burial. Similarly, at Żerniki 27 (*165*), only one additional bone from a second adult individual was discovered. In both cases, these remains may derive from a nearby destroyed grave (*164*) (pers. comm. Ł. Pospieszny). But given similar observations in other Corded Ware regions (e.g. cluster 10), these graves alternatively might indicate practices of successive interments in graves that were intended to be shared with partial remains of 'ancestors' (*166*, *167*).

In total, 20 burial events were included in our analysis, of which the majority of dates were derived from human bone samples. For Pikutkowo 6 (*163*), it must be mentioned that the listed radiocarbon samples can be safely linked to the context but not to specific individuals in the grave (*168*)(pers. comm. Ł. Pospieszny). Furthermore, it must be mentioned that the individual from grave 1 at Głuchowo 1 (*169*) was dated via bone carbonate in the skull bone, given the low availability of bone collagen. The only charcoal sample within the cluster dataset comes from grave 30 in Pasewalk 109 (*170*) and is directly associated with the grave pit. At Krusza Zamkowa 3 (*171*), two dates from animal bone plates were combined using the RCombine function in OxCal v. 4.4. The human bone sample in this grave was dated as well but was demonstrated to have a significant aquatic reservoir effect (*172*, *173*). In addition to that, the burial events at Żerniki 27 (*165*) and Ciechrz 25 (*174*) were modelled by using the RCombine function. Data excluded from the cluster area only comprises three dates, which did not meet the selection criteria.

Despite the inclusion of both German and Polish lowland data in chronometric discussions since the 1990s (*174–176*), there remains a significant imbalance in the considered data after careful sample selection. After excluding several dates from megalithic contexts, a particular dominance of data from the Polish lowlands is recognisable. Our analysis confirms the previously proposed long chronology of Corded Ware in the region and even extends it towards the end, resulting in the most prolonged multimodal probability distribution among all Corded Ware clusters. Previous discussions on the German data suggested that the emergence of Corded Ware-typical single inhumation burial practices occurred between 2900-2600 BC, with a concentration of dates until 2400/2300 BC and indications of continuance until 2200 BC (*175–177*). For Kujavia and Greater Poland, on the other hand, previous chronologies proposed four stages of Corded Ware presence between approximately 3050-2200 cal BC (1σ-range) on the basis of summed probability distribution (*159*, *174*).

According to our OLE models, an emergence of Corded Ware can be expected in the cluster region from c. 2883 cal BC onwards, followed by a steady increase in probability between 2800-2700 cal BC. A second, more pronounced increase in probability occurs between 2550-2200 cal BC, aligning only partially with the proposed fourth stage of Corded Ware in the eastern cluster region, traditionally viewed as the most localised stage of 'classical' Corded Ware in the region (*159*, *174*). After this period, our model indicates a gradual decrease in probability, with the most probable end-date of Corded Ware burial customs around 2031 cal BC. This confirms that Corded Ware in the cluster region co-existed with TRB and Globular Amphora communities of the first half of the third millennium BCE and continued into the Early Bronze Age (*159*, *174*, *178*).

Among the burial events selected for analysis, six individuals underwent archaeogenetic analysis in previous studies (*3*, *12*, *168*). All individuals revealed a steppe component in their ancestry to varying degrees, although samples from the multiple burials at Pikutkowo 6 hinted at the complexity of the genetic formation process of Corded Ware-associated individuals. While individuals N44 and N45 foreshadowed findings by Ringbauer *et al.* (*89*) and already indicated a Globular Amphora and Yamnaya admixture scenario, two other individuals from the same grave (N47, N49) exhibited higher genetic affinity to Western hunter-gatherers than to steppe individuals. This contrasts with other published Corded Ware-associated individuals from the Eastern Baltic and Germany (*168*). Another notable case is individual poz81 from the multiple grave E8 at Obłaczkowo 7. Thus far, the sampled male represents one of the earliest Y-chromosome R1a-carriers among Corded Ware-associated individuals in Europe. This haplogroup subsequently became characteristic for the majority of Corded Ware-associated males across Europe and is proclaimed to derive from hunter-gatherer and Eneolithic communities of Eastern Europe (*12*, *179–181*).

Cluster 8 (Scandinavian Peninsula) has been taken separately from cluster 11 (North-West European Plain), because archaeologically these clusters represent the Swedish-Norwegian Batte/Boat Axe Culture and the Single Grave Culture respectively. Both are part of the wider Corded Ware complex. While there are similarities, the Battle Axe Culture has a different material culture than the Single Grave Culture, particularly regarding ceramics and battle-axes (*182*). Moreover, funerary traditions differ; whereas barrow graves are overrepresented in the Single Grave Culture, with a dominant burial orientation of east–west, flat-grave cemeteries are typical for the Battle Axe Culture, with a dominant burial orientation of north–south (*182*, *183*). An issue with the Battle Axe Culture is the scarcity of absolute dates: in 2003, there were only seven dates of varying quality, only three of

which were from graves (*28*). Since then, more absolute dates have become available (*184*). In our study, we have included 13 dated burial events from 11 sites in Sweden. Four of the included burials have been sampled for aDNA (*3, 5*), while three aDNA samples have been excluded as they were not from unequivocal Battle Axe Culture contexts (*3, 11, 12*). In total, 12 dates have been excluded during our selection process.

Battle Axe Culture burials typically consist of single inhumations, buried in a crouched position in a wooden coffin or log or with a stone packing, in a flat-grave cemetery. Typical grave goods are flint axes, rounded beakers, stone battle-/boat-axes, flint blades, antler chopping weapons, antler daggers, arrowheads, bone awls and amber beads (*182, 183, 185*). While gender-specific body positions, orientations and grave goods are often claimed, this cannot be confirmed by the 18 osteologically determined from Scania (*160, 185*). Moreover, multiple graves are also known for the Battle Axe Culture, and two graves have been included in our dataset. There are also a few known cremation graves (*183, 185*).

The Battle Axe period has been divided into phases by Malmer (*186*), whose typochronology still prevails in Swedish archaeology. However, due to the few available C14 dates, his periodisation has not been corroborated with absolute dates (*182, 183, 187*). The Battle Axe period is generally considered to last from c. 2800-2300 BCE (*187*). Our study refutes this early start as our models demonstrate. The OLE reconstructs a much later start date for Corded Ware burial rites in Sweden, at c. 2695 cal BCE. According to the KDE, there is one large peak in probability for Corded Ware burial events between c. 2600 and 2400 cal BCE, and then a smaller peak between c. 2200 and 2100 cal BCE, before steadily decreasing. The OLE predicts an end date of c. 2018 cal BCE.

Cluster 9 (Middle Elbe-Saale region) encompasses Corded Ware graves from the Middle Elbe-Saale region and the Thuringian Basin within the broader expanse of the Central German Uplands region. A large number of radiocarbon dates derive from sites along the Saale River, particularly the undulating 'Querfurt plateau' (= 'Querfurter Platte') and its surroundings near the Saale-Unstrut confluence. Altogether, 88 burial events were considered in the analysis, while 16 dates from nine sites were excluded because of the developed criteria.

Given the abundance of Corded Ware records and materials obtained from the cluster region (*188–196*), the Central German Uplands region represents one of the core concentrations of European Corded Ware (*142*). At the same time, many high-quality radiocarbon dates are available from this area, owing to regional efforts of heritage state offices (as in Saxony-Anhalt, see (*197*)) or major dating programs conducted in the 1990s (*198*) aimed at clarifying the absolute chronology of Corded Ware in Central Germany.

Within the region, the prevailing burial customs involve single inhumations in crouched body positions along the west–east to south-west–north-east axis. Deviations from the norm are exceptional and only known in small numbers (see e.g. Bleckendorf (*199*)). Typically, males were buried right-flexed in west–east orientation, while females were buried left-flexed in east–west orientation, both facing south (*149,*

200). In the dataset, single inhumation graves represent the majority of graves, while 17 % of considered burials are double- or multiple graves. Well-studied examples, such as grave 98 from Eulau 6, containing four individuals of a nuclear family (*201*), or feature 25645 from Oechlitz consisting of five inhumations (*202*) are included in the latter category. Only a few graves reveal unambiguous evidence for mound cover (e.g. Auleben-Solberg (*190*)) or indications thereof (e.g. Oechlitz feature 25815 (*203, 204*)). At the same time, the bulk of burials appears to represent flat graves. Typical Corded Ware grave goods of the region include cord-ornamented beakers, amphorae, and (footed-) bowls, alongside stone battle-axes (especially facetted axes), polished (flint) flat axes, and an array of flint tools. Further, bone tools like chisels and awls feature regularly in Corded Ware grave tool kits. Typical ornaments are represented by bone pins, beads (made from antler, bone or amber), copper spiral rolls and rings. However, the most common ornaments are identified with perforated animal teeth (usually from canids or boar) and paillettes or discs made from shells, which can be part of large assemblages, a regional peculiarity which almost exclusively features in female burials. Boar tusk pendants/lamellae, on the other hand, are often found in male burials (*204*).

Previous absolute chronological work pinpoints the onset of Corded Ware around 2750 cal BC, with a duration until 2100/2000 cal BC (*198*), and a concentration of dates in the period between c. 2460-2200 cal BC (*28*). According to our models, the OLE attests an initiation of first Corded Ware burials in the cluster region after c. 2908 cal BC. As indicated by the multimodal KDE, a swift increase occurred post c. 2850 cal BC, leading to a culmination of burials between c. 2600 and 2460 cal BC. Hereafter, a fade-out in the KDEs emerges until c. 2280–2135 cal BC with a definite discontinuance of Corded Ware burials around 2060 cal BC, as estimated by the OLE. This stretches the boundaries of currently established relative chronologies for Corded Ware in the region on both ends beyond 2800-2200 BC. Nevertheless, it can be stated that the majority of Corded Ware graves in the region most-likely dates in the period between 2600 and 2460 cal BC.

Among the radiocarbon-dated burials from the curated cluster dataset, 17 individuals from 11 burial events were previously archaeogenomically analysed (*2, 31, 179, 201*). All considered samples revealed ancestry components with affinities to Yamnaya-associated populations in the European steppes. Interestingly, all analysed samples also date into the culminating 'main' phase of Corded Ware distribution after c. 2600 cal BC, and thus not into the earliest stage of Corded Ware. This is compelling given the high amount of steppe-related ancestry still found in a late Corded Ware individual from Esperstedt (I1536, feature 4098; (*181*)).

A noteworthy observation can be found in the study by Haak *et al.* (*2*), where it was cautioned that Corded Ware individuals from (Central) Germany could not directly trace back their ancestry to sampled Yamnaya individuals from Samara (*2*). Further, a subsequent study by Papac *et al.* (*13*) identified good model fits for subtle (c. 5-15%) allele sharing between Central German Corded Ware individuals from Esperstedt with (Middle-)Neolithic and Pitted Ware-related groups from (north-)Eastern Europe, when looking for a third ancestry source in proximal 3-way mixture models. This implies that Corded Ware individuals from Esperstedt carried some ancestry related to Latvia_MN/ Ukraine_Neolithic/ PittedWare sample groups, not present in both Yamnaya-associated individuals and European Neolithic/Middle Neolithic farmers known thus far (*13*). Recent genomic studies have provided further insight, revealing that the genetic ancestry of European Corded Ware groups is approximately 25% derived from Globular Amphora-associated individuals and around 75% from Yamnaya-associated individuals. This suggests that Globular Amphora groups played a significant role in the formation of

Corded Ware populations (*5, 89*). Archaeologically, the influence of Globular Amphora groups on the formation of Corded Ware, as well as the connection between Globular Amphora and Yamnaya communities in the western (forest) steppe region, had been discussed prior to the genetic discoveries (*33, 205–208*). In the context of the ongoing debate regarding links between Globular Amphora and Corded Ware communities, the grave from Groß-Weißandt is particularly noteworthy, as it fosters the Globular Amphora connection by encompassing a typical globular amphora next to a Corded Ware beaker while dating into the early Corded Ware phase.

Cluster 10 (Main-Danube region) comprises 51 dated Corded Ware burial events from 21 sites in a cluster region, that encompasses large parts of southern Germany (Bavaria and Baden-Württemberg) and is delineated by the drainages of the Main River in the north and the Danube in the south. Four graves in our dataset have been sampled for aDNA; two additional dates have been excluded because they were from graves without any grave goods (*3, 209*). In total, 11 dates have been excluded due to our developed selection criteria. Furthermore, a poor model agreement ($A \leq 60$, see methods) was observed, for the dated burial event in Haunstetten – Unterer Talweg 121 (*209*), which is why it had no effect on the rendering of the interpolation surface in figure 1.

The Corded Ware burial tradition of the Main-Danube region predominately consists of single burials in flat-grave cemeteries; barrows are rare (*33, 210*). Burials are typically placed in a crouched position, oriented east–west (females)/west–east (males) facing south (*210*). Typical grave goods consist of stone axes, beakers, flint blades and flint daggers, but arrowheads, battle-axes, flint flakes, perforated animal teeth and animal bones are also common (*211*). Shared graves occur frequently, and this is reflected in the 16 non-single graves that are included in our dataset (c. 31%). Excavations in the Lech valley (Bavaria), in the Danube catchment area, have shown that this area was densely populated in the second millennium BCE, with numerous large flat-grave cemeteries surrounded by hamlets, a trend which already started in the third millennium BCE with a few Corded Ware graves and numerous Bell Beaker graves (*212, 213*). Further north, in the Tauber valley (Bavaria and Baden-Württemberg), in the Main catchment area, the largest Corded Ware cemeteries of southern Germany have been found (*166*). While there are clear similarities between the Corded Ware graves in the Tauber and the Danube catchment areas, there are also many striking deviations from the 'Corded Ware norm', such as variable body positions and less gender differentiation, with both men and women mostly buried on their left side (*149, 166, 210, 211*). Due to the high density of well-preserved burials from the third and second millennia BCE, the Main-Danube region, and particularly the Lech valley, is featured in many recent studies employing scientific methods such as stable isotopes analyses and aDNA (*21, 209, 214–216*).

The Corded Ware period in southern Germany is traditionally thought to last from c. 2800/2750 to 2600/2550 BCE (*210*). Earlier attempts at defining a periodisation of the south German Corded Ware were strongly influenced by Danish typochronologies as well as dendrochronological dates from the Alpine region (*28, 210*). Due to the inclusion of new C14 dates, our study contradicts this periodisation, with an earlier start date and a much later end date. The OLE suggests an early emergence date for Corded Ware burial customs in the Main-Danube region, around c. 2918 cal BCE. The KDE probability of Corded Ware burial events is fairly moderate between c. 2885-2755 cal BCE, before increasing considerably between c. 2600 and 2450 cal BCE. A low probability of burials persists until ca. 2100 BC and the OLE predicts an end date of c. 2030 cal BCE.

Cluster 11 (North-West European Plain) consists of 46 dated Corded Ware burial events from 44 sites, from the Netherlands, Denmark and north-western Germany. Due to the poor preservation of bone in these regions, most radiocarbon dates are from charcoal (or wood), and thus, some level of uncertainty must be taken into account (see SI4). Only three dates are from human bone/teeth. 100 dates have been excluded during our selection process.

The regional name of the Corded Ware complex in the Netherlands, Denmark and north-western Germany is Single Grave culture (*Enkelgrafcultuur*, *Einzelgrabkultur*, *Enkeltgravskultur*). These regions have been clustered together in our analysis, on the grounds that graves throughout this area show similar funerary customs and material culture, and there are clear indications for exchange throughout this area (*54*). As its name suggests, the Single Grave Culture typically consists of single inhumations, although there are seven occasions in our dataset which represent double or multiple burials (c. 15%). Individuals are typically buried in a crouched position, oriented east–west with individuals facing south. Graves often have structural elements such as a (charred) wooden coffin and are often placed in or under a barrow, which often have a wooden or stone enclosure surrounding it and are often reused later in prehistory. Grave goods typically consist of beakers with a cord decoration, stone battle-axes, flint axes, flint blades and amber ornaments (*54, 217, 218*).

The Single Grave Culture has a long research history, with well-known typochronologies published by Müller (*219*), Glob (*220*), Struve (*221*) and Van der Waals & Glasbergen (*222*). In the 1990s, large-scale radiocarbon dating programmes took place (*223, 224*). The typochronologies and absolute dates combined suggested a duration of the Single Grave Culture from c. 2850-2400 cal BCE (*217, 225, 226*). This periodisation is more or less confirmed by our models. The OLE indicates the start of Single Grave Culture burial customs after c. 2872 cal BCE. The KDE shows a gradual increase in probability for Corded Ware burials between c. 2850 and 2600 cal BCE, with a distinct peak between 2600 and 2450 cal. BCE. The OLE predicts an end date of c. 2387 cal BCE.

Another consequence of the poor preservation of bone in this region is that aDNA samples are scarce. No Dutch or north-western German Corded Ware burials have been included in aDNA studies. While several Danish individuals have been included in aDNA studies, most cannot be unambiguously attributed to the Single Grave Culture and have therefore been excluded from our dataset. Of the Danish samples, only those from the megalithic site Gjerrild have been included in our study (*227*). The Gjerrild grave consists of at least ten individuals buried in a Bøstrup type cist (typologically dated to the late Single Grave Culture) with late SG material culture: two beakers, a flint adze, two amber beads and three flint arrowheads, one of which embedded in the sternum of one of the individuals. Although the Gjerrild grave is not located in Jutland, the 'core area' of Single Grave Culture, but on the Danish Isles, where late Funnel Beaker traditions prevailed until c. 2350 BCE (*228*), we have decided to include this site as its cultural attribution is unambiguously Single Grave Culture. Six individuals have been radiocarbon dated; the radiocarbon evidence suggests that the cist had been in use for over 300 years during the middle and late Single Grave period, and even into the Early Bronze Age. We included the date from the only individual with a completely terrestrial diet (individual 1), which is the second oldest date from the site. The other individuals had been affected by breastfeeding and/or marine diets.

2. *Bell Beaker*

Cluster 1 (Western Iberia) contains 22 dated Bell Beaker burial events from 12 sites from Portugal. On the basis of our selection criteria, 58 dates have been excluded from the analysis. Notably, the very early dates from settlement contexts (Cardoso, 2001) have not been included as our study takes the Bell Beaker as a funerary tradition (see SI4). Only one aDNA sample has been included in our dataset (I1970), as the other sampled individuals are from uncertain Bell Beaker contexts (*4*).

The Bell Beaker funerary context in Portugal mainly consists of collective or accumulative tombs. Only few new funerary structures were built, and most graves consist of reused funerary spaces from the Neolithic/Chalcolithic periods (*229*). These structures are mainly megalithic tombs, especially in northern Portugal (*230*), and natural or artificial caves known as hypogea and *tholos*-type megalithic monuments in the Tagus Estuary and the Alentejo areas (*231, 232*). Additionally, pit graves appear in the vicinity of the fortified settlements (*233*). Single graves do occur however, and nine of them have been included in our dataset (c. 41%). There are no preferred body positions or orientations (*234*). Typical grave goods consist of Maritime Bell Beakers, copper awls, copper knives, flint arrowheads and V-perforated buttons, with other typical Bell Beaker artefacts such as wristguards, copper daggers, Palmela points, gold ornaments and boar's tusks introduced later (*37*). Inhumations are not placed in a sex-specific position or orientation, but women are associated with copper awls and V-perforated buttons, and men with weapons (*234*). Estremadura and Alentejo areas were clustered together due to the continuous use of Chalcolithic fortified settlements and graves during the Bell Beaker period, in addition to the similarity in material culture, including imported objects (*235, 236*).

A ‘proto-Beaker package’ is considered to have developed in central Portugal around 2700/2600 BCE (*37, 237*). Our study suggests a slightly later emergence of the Bell Beaker burial custom, as the OLE indicates a start date after c. 2616 cal BCE. The KDE probability shows a peak between c. 2505 and 2100 cal BCE. Additionally, the KDE suggests a low probability for Bell Beaker burial events between c. 1900 and 1745 cal BCE, after which the OLE suggests an end date of c. 1626 cal BCE.

Cluster 2 (Central and South-East Iberia) encompasses 48 dated Bell Beaker burial events from 15 sites from central and south-eastern Spain; 45 dates have been excluded in our selection process. While 16 aDNA samples have been included in our dataset, 33 others were excluded due to uncertain or pre-Beaker contexts (*4, 179, 238–240*). For the measured age of the burial event in Área 36, EI-03-VII (Área funeraria 2) at Camino de las Yeseras (*4*), a poor model agreement ($A \leq 60$, see methods) was observed, which is why it had no effect on the rendering of the interpolation surface in figure 1.

Chalcolithic funerary practices continue during the Bell Beaker period in central Spain. These funerary practices show strong localism (*15, 234, 241, 242*), encompassing both collective accumulative or single

graves in megalithic tombs, pits, natural caves or artificial caves/hypogea. In the south-east Mediterranean Coast, the reuse of natural caves or *tholos*-type megalithic monuments along with simple megalithic monuments is contemporary to the construction of pit graves near settlements and mass graves during Early and Late Chalcolithic, including the Bell Beaker period (*243–245*). Sixteen single graves have been included in our study (c. 33%). There are no preferred body positions or orientations, and there is no gender distinction in this either. However, women are typically associated with copper awls and V-perforated buttons, and men with weapons. Grave goods typically consist of Bell Beaker pottery, copper objects such as awls, tanged daggers and Palmela points, archery equipment, gold jewelry and elephant ivory objects. Cinnabar powder occurs regularly in central Spain (*234*).

Central Spain is considered one of the main regions of the Bell Beaker phenomenon, associated with a characteristic regional style of Bell Beaker pottery known as Ciempozuelos (*246, 247*). Due to the scarce number of reliable dates in south-east Mediterranean Coast (nine dates from four graves), we decided to cluster them with the dates from central Spain. This association is based on the reuse of funerary structures, the occurrence of pit graves in settlements as individual or double/triple burials, and the similarities in the material culture, including import objects such as Moroccan elephant ivory (*235*).

The Bell Beaker phenomenon is typically thought to appear from 2850/2750 BCE and last until 2000/1900 BCE (*241*). Our study suggests a much later emergence of the typical Bell Beaker burial custom, as the OLE models a start date of c. 2472 cal BCE. The highest probability for Bell Beaker burial events falls between c. 2450 and 2200 cal BCE according to the KDE. Between c. 2150 and 2000 cal BCE a second, smaller peak in KDE probability is visible. The OLE predicts an end date of c. 1922 cal BCE.

Cluster 3 (North-West Mediterranean region) comprises 12 dated Bell Beaker burial events from 11 sites from north-eastern Spain, south-eastern France (French Midi, Centre-West and the Auvergne)(*248*) and northern Italy. These regions have been clustered together because the area north of the Ebro River, the French Midi and the Catalonia region is traditionally considered to be a regional group (*249, 250*). The megalithic tomb Atalayuela has been included in this cluster, and not in cluster 2, due to its proximity to the Ebro River, which borders other sites of cluster 3 at its lower (coastal) reaches. Due to the scarce number of reliable dates from (northern) Italy, the two included Italian dates have been added to this cluster; archaeologically, however, it can be argued that there are known Italian influences in the Bell Beaker phenomenon in the Provence (*251*). On the basis of our selection criteria, 51 dates have been excluded. Only one aDNA sample features in our dataset; five others were excluded as they derive from uncertain Bell Beaker contexts (*4, 252–254*).

The Bell Beaker phenomenon in southern France is characterised by a large variety in funerary practices, dominated by collective graves in natural and artificial caves and dolmens, but with occasional individual graves in barrows, flat-grave cemeteries and domestic pits (*248*). Mainly decorated pottery is given as grave goods, but archery equipment, copper awls and daggers, V-perforated buttons and arched pendants are common as well (*255*). In north-eastern Spain, collective graves in pits, cists, dolmens, caves and hypogea, as known in the preceding Chalcolithic, continue in the Bell Beaker period (*256*). Hypogea and modified caves are most common. Here, grave goods

typically consist of pottery, copper awls, daggers and Palmela points, and archery equipment, and more rarely, cinnabar powder (*234*). There are no preferred body positions or orientations in north-eastern Spain, and no clear gender distinction (*234*). Single graves rarely occur in southern France (*255*) and north-eastern Spain (*234*), and are more common in northern Italy, where wooden mortuary houses are frequent (Nicolis, 2001). Five single graves have been included in our dataset (c. 42%). One of those is Georges Besse II (France), which has been included to reach sufficient sample size, despite having no grave goods. Here, the crouched single inhumation in a stone cist can be considered typical for the Bell Beaker period or Early Bronze Age (*257*), and from its date can be considered Bell Beaker.

Traditionally, the Bell Beaker period in this region is divided into four phases, on the basis of Guilaine's classification (*258, 259*). More recently, the Bell Beaker phenomenon is divided into only three phases on the basis of typochronology and radiocarbon dates (*260*): an early phase (c. 2550/2500-2400/2350 BCE), a recent phase (c. 2400/2350-2150 BCE) and a late phase (c. 2150-1950 BCE). The recent phase is characterised by regional groups such as the Pyrenean group and the Rhône-Provence group in the French Midi (*260*).

Our study proposes a much earlier starting date for Bell Beaker burial customs; the OLE indicates a start date of c. 2901 cal BCE. The KDE suggests a low but steady increase in probability for Bell Beaker burial events until c. 2600 cal BCE, after which a peak occurs between c. 2450 and 2050 cal BCE. The OLE predicts an end date of c. 1997 cal BCE.

Cluster 4 (Seine-Loire River basins) consists of 17 dates from 16 sites from north-western and central France. During our selection process, 39 dates have been excluded. Only one aDNA sample has been included; two others have been excluded because the attribution to the Bell Beaker complex was uncertain.

The Bell Beaker complex in northern France is considered to be roughly divided into an eastern and western half, with collective tombs being more common along the Atlantic coast, thus showing more connections to southern France and Iberia, and the eastern half dominated by individual burials which show influences from the Rhine valley and Central Europe (*261*). However, there are not many radiocarbon dates from collective tombs in western France, and individual graves in pits and cists are known and are similar to the eastern half of France (*262*). Therefore, we have decided to cluster these regions together, with the exception of dates from the Alsace and Lorraine, which have been clustered together with the southern German dates in cluster 8 (see below). The individual graves in northern France consist of inhumations buried in crouched and supine flexed positions, oriented along the east-west to north-east–south-west axis, in flat-graves with often wooden grave structures (*261*). Men are typically placed on their left side and women on their right side (*263*). Grave goods include beakers, and flint daggers in the early Bell Beaker period and later archery equipment and V-perforated buttons (*261*). Five dates in our dataset are from non-single graves (c. 29%) and two are cremation graves (c. 11%).

The prevailing periodisation of the Bell Beaker period in northern France consists of an early phase (2500-2400 BCE), a recent phase (2400-2150 BCE) and a late phase (2150-1950 BCE) (*260, 261*). Our study suggests that the Bell Beaker burial custom starts earlier than typically thought: the OLE models a start date of c. 2634 cal BCE. The KDE indicates two peaks in probability for Bell Beaker burials: between c. 2600 and 2400 cal BCE and between c. 2300 and 2100 cal BCE. The OLE predicts an end date of c. 2006 cal BCE.

Cluster 5 (Southern Britain) consists of 75 dated Bell Beaker burial events from 58 sites from England and Wales. Britain has been divided into clusters 5 (Southern Britain) and 6 (Northern Britain). Just as on the continent, the Bell Beaker burial custom in Britain is characterized by single inhumation burials with a standardized grave inventory. Yet there is a difference between the southern half, which is characterized by north–south oriented burials with Maritime Bell Beakers, and the northern half, with its east–west oriented burials and All-Over-Cord Beakers (*264*). In contrast to Britain, the Bell Beaker burial custom in Ireland is not characterized by single inhumation graves, but by megalithic graves (*265*); unfortunately, all dates from Irish graves have been excluded from this study due to contextual problems with these dates (*266*).

The majority of dates in the cluster region are from the *Beaker People Project* (hereafter *BPP*) (*53*); pre-Beaker dates and dates from graves with Food Vessels or from the Early Bronze Age included in this project have been omitted from our dataset, as we sought out typical Bell Beaker dates. In total, 67 Beaker dates have been excluded on the basis of our selection criteria. 19 aDNA samples have been included, while eight aDNA samples have been excluded due to being from uncertain Beaker contexts (*4, 267*). A poor model agreement ($A \leq 60$, see methods) was observed for the dated burial events F919 (secondary) at Radley Barrow Hills (*264*) and grave 40557 at Northumberland Bottom, Southfleet (*53*), which is why they had no effect on the rendering of the interpolation surface in figure 1.

The typical Bell Beaker burial package in Britain consists of a single inhumation buried in a gender-differentiated crouched-flexed position in a flat-grave or under a small barrow, together with a Beaker and sometimes other grave goods: flint flakes, knives, scrapers, strike-a-lights and arrowheads, bone points, antler spatulae, stone wristguards, copper knives/daggers, awls and ornaments, beads and buttons of various materials, and more rarely, gold ornaments (*43, 268, 269*). While males are often given the whole array of grave goods, females are rarely given anything other than a Beaker, and then typically flint or bone tools or a copper awl (*270*). Males are generally placed with their head to the north and females with their head to the south (*270*).

In addition to the typical crouched single inhumations described above, other Beaker funerary traditions consist of cremation graves, disarticulated burials and multiple graves (*43*). In our dataset, seven non-single graves have been included (c. 9%), one of which includes a cremated child and a neonate in a Beaker buried together with an inhumed child (Radley Barrow Hills, grave F919; (*271*)).

The *BPP* found that the Bell Beaker phenomenon starts around 2450-2325 cal BC in England and around 2375-2325 cal BC in Wales (*272*). Our study confirms the early part of this range, with the OLE

indicating a start date of c. 2448 cal BCE. The KDE finds one big peak in probability for Bell Beaker burial practices between c. 2350 and 2100 cal BCE, and the OLE predicts an end date of c. 1982 cal BCE. This conforms to the general periodisation of the ‘Beaker period', c. 2450-1950 BC (*43*). However, Jay *et al.* (*272*) write that the last dated use of Beakers in graves in England took place in 1850-1690 cal BC and in Wales in 2010-1995 cal BC. Because we have excluded dates from clear Early Bronze Age graves, e.g. those with typical Early Bronze Age artefacts, our model predicts an earlier end date.

Cluster 6 (Northern Britain) comprises of 57 dated Bell Beaker burial events from 48 sites from Scotland and northern England. The majority of the dates are from the *Beakers and Bodies Project* (hereafter *BBP*; (*53*)); as is the case for cluster 5, we omitted pre- and post-Beaker dates. Forty-one dates have been excluded from our dataset. Five aDNA samples have been included and 11 excluded as they were from uncertain Beaker contexts or did not have any grave goods (*4*). For the age of the burial event in Lochend (*53*), a poor model agreement (A ≤ 60, see methods) was observed, which is why it had no effect on the rendering of the interpolation surface in figure 1.

Bell Beaker burials in Northern Britain typically consist of flat graves with single inhumations buried in crouched positions in stone cists, although earlier megalithic tombs and monuments were also reused (*270, 273*). Males are generally buried on their left side, with their head to the east, and females on their right side, head to the west (*270*). Beakers are the most common grave goods, but other grave goods can also accompany the dead: mainly flint artefacts such as flakes, knives and arrowheads, with the typical Beaker artefacts such as wristguards occurring much more rarely (*273*). Additionally, a distinct local practice in north-east Scotland is the inclusion of unworked pebbles in the grave (*273*). While single inhumations are the standard in Northern Britain, six non-single graves (c. 8%) have been included in our dataset. Moreover, four cremation graves have been included.

The *BBP* found a start date for the Bell Beaker phenomenon around 2415-2315 cal BC in Scotland (*272*). Our study confirms this range, as the OLE demonstrates a start date of c. 2381 cal BCE for Bell Beaker burial practices in Northern Britain. The KDE suggests a large peak in probability for Bell Beaker burial events between c. 2300 and 2150 cal BCE, followed by a low probability between c. 2010 and 1900 cal BCE, indicating a longer persistence of beaker burials. The OLE predicts an end date of c. 1899 cal BCE, which corroborates the last dated use of Beakers in Scotland in 1900-1745 cal BC according to the *BBP* (*272*).

Cluster 7 (North-West European Plain) consists of 25 dated Bell Beaker burial events (22 sites) from the Netherlands and “Bell Beaker-like” graves in Denmark. The Bell Beaker phenomenon in Denmark is not as pronounced as in other regions and is considered to be “a material culture of Bell Beaker derivation” (*274*) during the Late Neolithic I/early Dagger period rather than a typical Bell Beaker group itself. Graves included in this cluster are those with LN I flint daggers as well as graves with straight-walled Beakers from the Upper Grave Period of the Single Grave Culture, as both of these artefacts are considered to be typical for this “Bell Beaker-like” phenomenon in Denmark (particularly north-western Jutland) (*217, 274, 275*). Contrary to the Bell Beaker typical crouched position of single

inhumations, Danish "Beaker-like" inhumation burials are often buried in a supine extended position, often placed in communal graves or as secondary graves in Corded Ware barrows (*276*). While the Bell Beaker phenomenon in the Netherlands is more "classic", with the typical Bell Beakers and archery sets as common grave goods (*54*), the dates from the Danish "Bell Beaker-like" graves had to be clustered together with the Dutch dates, because there would not be sufficient dates in a separate Danish cluster for the analysis. The shared Single Grave Culture background in both regions is further grounds for clustering these dates together. As with the Single Grave Culture graves, the Bell Beaker funerary tradition is associated with single graves and burial mounds, yet now with a new grave set including new pottery and metal objects (*54*). Despite inhumation graves being the standard, nine graves in our dataset were cremation graves (c. 3%).

During our selection process, 69 dates have been excluded amongst which eight dates are from burial events in Belgium. Only two aDNA samples have been included in our dataset; 11 other aDNA samples have been excluded because of a lack of (diagnostic) grave goods and in one case, a possible marine reservoir effect (*4*). Furthermore, a poor model agreement ($A \leq 60$, see methods) was observed for the dated burial event in Højris, Ringkøbing (*224*), which is why it had no effect on the rendering of the interpolation surface in figure 1.

The Bell Beaker complex is thought to appear from c. 2450 BCE in the Netherlands and c. 2350 BCE in Denmark (*223, 274*). Our OLE roughly confirms this, suggesting a start date of c. 2462 cal BCE. The KDE indicates a large peak in probability for Bell Beaker burials between c. 2400 and 2000 cal BCE. The OLE predicts an end date of c. 1956 cal BCE.

Cluster 8 (Upper Rhine and Main-Danube region) consists of 36 dated Bell Beaker burial events from 26 sites in north-eastern France, Luxembourg and southern Germany – a region broadly delineated by the Upper Rhine, Main and Danube drainage systems. While 12 aDNA samples are included in this cluster, 12 others have been excluded due to the samples being from uncertain Bell Beaker contexts, or due to insufficient information about the context. In total, 28 dates have been excluded on the basis of our selection criteria.

The Upper Rhine and Main-Danube region has a very high density of Bell Beaker sites, although large Bell Beaker cemeteries are mostly confined to the Danube catchment area (*210*)(see also Corded Ware cluster 10). The dates from Lorraine, Alsace and Luxembourg have been clustered together with the southern German dates, because the Bell Beaker burial custom in these regions is characterised by individual burials which show influences from the Rhine valley and Central Europe (*261*). Yet in Alsace, inhumations are typically oriented north–south, which stands out compared to other regions (*261*). In the region as a whole, however, the Bell Beaker burial tradition consists of single inhumations in flat graves, in crouched positions, with males buried on their left side and females on their right side. Wooden coffins are common in north-eastern France (*277*). Non-single graves are also common, of which nine are included in our dataset (25%). Typical grave goods are beakers, copper daggers, flint flakes, flint arrowheads, wristguards, stone axes, bone bow-shaped pendants, V-perforated buttons, beads and animal bones (*210, 261*).

In southern Germany, the Bell Beaker phenomenon is thought to begin from c. 2550 BCE and last until c. 2150 BCE, smoothly transitioning into the EBA (*212, 213*). In north-eastern France and Luxembourg, the Bell Beaker phenomenon is thought to start around 2500 BCE and continue for longer, until c. 1950 BCE (*261*). Our study deviates from the southern German periodisation and partially agrees with the north-eastern French periodisation. The OLE suggests an earlier start date, of c. 2634 cal BCE, and an end date of c. 1982 cal BCE. According to the KDE, the highest probability of Bell Beaker burials date in the range of c. 2400-2100 cal BCE.

Cluster 9 (Lower and Middle Elbe-Saale region) comprises of Bell Beaker burials from the Central German Uplands region and the adjacent North German plain, that are situated in the Lower and Middle Elbe drainage system. However, most of the sites are concentrated in the Circum-Harz region, between the Magdeburg Börde to the north and the Thuringian Basin to the south. In total, 102 dated burial events from 36 sites were analyzed. Radiocarbon dates were derived exclusively from human bone samples. For graves 3/19 from Halle-Trotha (*278*) and grave 772 from Wetzendorf 4 (*279*), an RCombine operation in OxCal 4.4 was successfully conducted. After modeling the dataset, poor model agreement was observed for features 1544 from Hettstedt (*280*) and 1265 from Quedlinburg XII (*281*), therefore having no effect on the interpolation surface in figure 1 (see methods). Due to the established criteria, 28 sites were excluded from the analysis.

The Bell Beaker burial customs in the region are marked by a predominant single inhumation burial rite, in which individuals are buried in crouched positions along the north–south axis. Females were typically right-flexed, with their heads facing south, while males were left-flexed, with their heads to the north, both facing east. The inhumations most commonly were interred in simple flat graves, which at times, were furnished with stone slab frames (e.g. grave 2 in Osmarsleben (*282*)) or wooden chambers, which themselves were occasionally covered by stone slabs (e.g. feature 10030 in Rothenschirmbach A38 (*283*)). Occasionally cemeteries reveal linear burial alignments, in which the graves are arranged along a roughly north–south-oriented axis (see e.g. Oechlitz (*284*)). In rare cases, there is even evidence for mound covers (see e.g. central barrow grave from Tarthun (*278*)) or circular ditches (e.g. feature 28051 in Oechlitz moser 2017(*284*)). Non-single graves also appear in the cluster region, such as the multiple burial from Waren, containing three individuals (*176*) or the double graves from Hettstedt (feature 2424) (*280*), Egeln Galgenberg (grave A) or Egeln-Mühlenbreite (*282, 285*). Of special interest is the cremation grave from Oldenstadt, Kr. Uelzen, which contained the remains of four individuals (*285*). Alongside the cremation grave from Eythra/Zwenkau (*286*), these provide evidence of a less frequently practiced cremation rite among Bell Beaker communities in this region (*204, 282*).

The spectrum of typical grave goods includes typical bell beakers, (footed-) bowls, cups, and jugs, as well as lithic artefacts such as flint blades, flakes, scrapers, arrowheads, but also wristguards. Bone tools like awls and chisels, and copper items such as daggers and awls/pinchers, are also found occasionally. Special items include pins and beads made from amber or bone, cushion stones, stone arrow shaft smoothers, and gold/electrum ornaments (small rings or sheets), which, in rare cases, co-occur in the same burial (e.g., cremation grave from Eythra/Zwenkau; Conrad, 2019). Furthermore, animal bones as potential food offers feature regularly (*202*).

Substantial efforts have clarified the absolute chronology of Bell Beaker communities in Central Germany (*197, 198*). The latest models for Saxony-Anhalt suggest a start around 2550 cal BC and an end around 2200/2100 cal BC (*197*), although earlier assessments (*198*) even suspected a potential end around 1900 cal BC. According to our OLE model, an emergence of Bell Beaker burial rites in the region can be expected from no earlier than c. 2578 cal BC onwards. After that, the probability of the bimodal KDE distribution increases steadily. The major probability peak in the model is found during a 200-year interval between 2350-2150 cal BC. After that, a sharp probability drop becomes recognisable, before the OLE-model suggests a discontinuance of Bell Beaker customs after c. 2079 cal BC. This demonstrates that Bell Beaker and Corded Ware burial rites co-existed in the Central German Uplands during the second half of the third millennium BCE.

Of the radiocarbon-dated burial events analysed, 11 individuals underwent genomic analysis in Brandt *et al.* (*31*). The study noted that Bell Beaker-associated individuals could be distinguished from Corded Ware-associated individuals by a relatively frequent mitochondrial haplogroup (H), which was absent in Central European hunter-gatherers but prevalent in ancient populations of the Iberian Peninsula since the Mesolithic. This was seen as indicative for a genetic influx from south-west Europe (*31*), though a later study by Olalde *et al.* (*4*) identified steppe ancestry amongst Central European Bell Beaker individuals and challenged this finding (cf. the individual from feature 19614 in Quedlinburg VII 2, which reveals Y-haplogroup R1b1a1a2 (= R1b-M269) (*31*), a lineage now associated with the arrival of steppe populations in Central Europe during the third millennium BCE (*2, 3*).

Cluster 10 (Upper Elbe-Isar region) encompasses sites located between the Upper Elbe and Lower Vltava rivers in the Bohemian basin, as well as those near the Danube-Isar confluence, where cemeteries extend along the edges of the Gäuboden region into the northern Lower Bavarian Uplands. The region roughly corresponds to the central part of the traditionally recognised 'Eastern Bell Beaker Province' (*38*). The decision to group sites around the Bohemian massif into a single cluster stems from their spatial proximity. It further allows for a compartmentalisation of the Elbe and Danube rivers, which enables observations regarding the spatiotemporal spread of burial customs along the rivers. As both rivers flow through multiple cluster regions, they likely played crucial roles in the transmission of Bell Beaker burial practices during the third millennium BC.

A total of 41 burial events were included in the analysis, primarily sourced from cemetery sites, which serve as the main source of information on Bell Beaker communities in the region (*287*). In all cases, radiometric measurements were conducted on human bone material of the buried individuals. The two dates of the individual from grave 53/80-1 at Radovesice XIII (*13*) were successfully combined using the RCombine function in OxCal v. 4.4. Due to the developed criteria, seven burial events were excluded from the analysis.

The burial practices of Bell Beaker communities in this region closely resemble those found in the Central German Uplands (cluster 9). Burials typically occur in small cemeteries, characterised by a predominant single inhumation rite, where individuals are buried in crouched positions along a north–south axis. Females were typically positioned on their right side with their heads to the south, while males were placed on their left side with their heads to the north, both facing eastward. Most burials occurred in simple flat graves, though stepped grave pits (e.g., grave 4335/H225 at Vliněves (*288*)) with

indications of organic coffins and graves with wooden constructions (e.g., grave 14 at Praha-Kobylisy, Ke Stírce St. (*4*)) are also documented. Unlike in the Central German Uplands, stone-framed graves were rare (*287, 289*). But also, the evidence for mound covers is very rare and only proposed for few graves surrounded by a circular ditch (e.g., grave 1 in Trieching (*290*)). Except for the potentially multi-staged burial from grave 77/99 in Tišice (Turek, 2006), non-single graves are almost absent in the cluster dataset, and in general seemed to have been a rare occurrence in the region (< 10%; (*289*)). Among the here considered burial events, there is also one cremation of a child in grave 269/06 (2290) at Tuněchody (*291*), which illustrates the supplementary role of the cremation burial rite in the regional Bell Beaker burial custom (*292*).

The typical range of grave goods includes bell beakers, footed bowls, cups, and jugs. Lithic artefacts are primarily found in the form of blades, flakes, scrapers, and arrowheads made out of flint. In addition to that, there are also stone wristguards which complete the widely proposed archery assemblage. Less frequent are bone tools like awls and chisels, and copper items such as daggers and awls/pinchers. The latter are often seen as evidence for metallurgists, and occur in burials of both males and females (see e.g. Tišice (*293*)). Ornaments include amber or bone beads, bone pins, as well as V-perforated buttons. Further there are small rings made of copper, electrum or gold, which more often feature in female burials (*47, 287*).

The absolute chronology of Bell Beaker societies in this region has been enhanced by recent archaeoscientific studies (*4, 13, 214*), which further included dates and basic context information from burials of yet-to-be-published sites (e.g. Praha-Ruzyně; Praha - Kobylisy, Ke Stírce St.; Radovesice XIII; etc.). Prior to this, the lack of data seriously hampered the establishment of an absolute chronology in Bohemia, which is why absolute chronologies from neighbouring regions as well as the earliest dates for the Únetice culture were used to anchor the Bohemian stage of Bell Beaker burials chronologically in the period between 2500-2300/2200 cal BC (*47, 287*).

According to our models, the temporality of Bell Beaker burials in this cluster region shares many similarities with Bell Beaker cluster 9, in which comparable temporal patterns were observed. Likewise, our OLE model proposes the emergence of Bell Beaker burial practices no earlier than c. 2577 cal BC. While a first small probability peak in the bimodal distribution of the KDE model becomes recognisable between 2500-2400 cal BC, there is a continuous probability increase, ultimately peaking in the period between 2300-2150 cal BC, followed by a sharp decline, ending with a discontinuance of Bell Beaker burials around c. 2105 cal BC, as estimated by our OLE model. This indicates that Bell Beaker burial customs were held up slightly longer than previously assumed and not only co-existed alongside Corded Ware burial practices in the earlier stages but also lasted into the beginning Early Bronze Age (*294*).

Of the included burial events in our analysis, 36 individuals underwent further archaeogenetic analysis (*4, 13*), making this cluster one of the most comprehensively studied Bell Beaker clusters in Europe. Results indicate that individuals from the cluster area exhibited varying degrees of steppe-associated ancestry (*4*), with a resurgence of Middle Eneolithic-like ancestry observed in Bohemian Bell Beaker individuals after 2400 cal BC (*13*). Furthermore, the study demonstrated that Bohemian Bell Beaker males and English Bell Beaker males (cluster 5) were not descendants of one another but instead diversified in parallel (*13*).

Cluster 11 (Oder-Vistula-Danube region) encompasses sites in the easternmost sphere of the European Bell Beaker phenomenon, extending to its periphery along the Danube in the Great Hungarian Plain and the Vistula River in the east. In the cluster's western sector, sites are concentrated in the Morava River basin. Along the Danube, sites are particularly clustered on the left banks near the south-eastern foothills of the Pilis Mountains and further downstream on Csepel Island (*295*). To the north-east, the cluster extends into the eastern North European Plain, where Bell Beaker burial sites concentrate along the Upper Odra in Silesia (*296*) and the Vistula (*297*), marking the easternmost boundary of the Bell Beaker phenomenon in Europe. Within this region, concentrations appear in Kujavia and in Lesser Poland, between the Vistula and the Holy Cross Mountains (*298*).

Bell Beaker contexts from these various regions are typically viewed in their respective regional settings, leading to the establishment of several group designations. Hungarian complexes are commonly referred to as part of the (Budapest) Csepel Group of Bell Beakers, while Moravian Bell Beaker sites are viewed separately, as are the Polish sites (*47*). In Poland, two groups are distinguished: Bell Beaker burials in Lesser Poland, often seen as influenced by Moravian Bell Beaker communities and associated with the Eastern Province of Bell Beakers (*297*), and burials in Greater Poland, particularly Kujavia, which are attributed to the Iwno group/culture. The latter is considered a local expression of Bell Beaker culture (*159, 298*). Due to its display of syncretism between Bell Beaker and late Single Grave Culture (= Corded Ware) in its early stage (particularly in terms of pottery inventory and –decoration) it is traditionally linked to the northern European Bell Beaker province instead (*298*). Given the limited sample size of the Iwno group, however, this group was not established as a separate cluster but was merged with other burial events from the traditional Eastern Province of Bell Beakers (*38*).

In total, 79 radiocarbon-dated burial events from 22 sites were considered for the analysis, with most samples derived from human bone material. Notably, burnt human bones were sampled from graves 23 and 37 at Stříbrnice 1 – Lopaty (Peška, 2012), while in grave 27 at Kyjovice Sutny II (*299*) wooden haft relics of a copper dagger were sampled. In ten cases, RCombine operations were successfully performed. Nine sites had to be excluded due to the developed criteria, which included the only radiocarbon-dated burial event from Austria, Poysbrunn (*300*), whose full BP age, standard deviation and laboratory number remain to be published. Further, four burial events were ineffective for the rendering of the interpolation surface in figure 1, due to poor model agreement of their radiocarbon ages below a threshold of $A \leq 60$. This included graves 501 and 516 from Pavlov-Horní pole (*301*), grave 37 from Stříbrnice 1 – Lopaty (*302*) and grave 1 from Strachów (*4*).

Burials in the eastern group of the Bell Beaker phenomenon display both commonalities and regional differences in burial customs. One of the most consistent elements in the cluster group is the orientation of the deceased, who were typically buried in contracted positions on their back or side, facing east. Males were generally buried left-flexed with their heads to the north, while females were right-flexed with their heads to the south, in line with the stereotypical Bell Beaker burial customs observed in Central Europe (*47, 297, 298, 303*). Burial orientations slightly deviated only along the Middle Danube, often following a NE-SW axis (*304*). Further, burials are commonly aggregated into small- to medium-sized cemeteries. In the southern parts of the cluster region, however, also considerably larger sites emerged, containing up to 150 graves (see, e.g. Hoštice in Moravia (*305*) or Szigetszentmiklós-Felső

Ürge-hegyi dűlő in Hungary (*304*)). On the Bell Beaker cemetery Budakalász in Hungary, even ~1,000 graves were discovered (*306*).

Similar to observations in cluster 9, graves were often aligned along an axis that coincided with the orientation of individual burials (*15*). Most common were rectangular flat graves for single individuals, while also a few ring ditches are known from sites in Moravia (*299*), Silesia (*296*) and Hungary (*304*). These may indicate the presence of ancient mound covers that did not always survive (*303*, *304*, *307*).

In the cluster region, non-single graves were also present. While feature 37 at Łojewo 4 (Poland) contained three individuals (*308*), there are further one antipodal double burial (grave 67 at Záhlinice 1 (*309*)) and one bi-ritual double burial (grave 570 at Pavlov-Horní pole (*310*)) in the cluster dataset. In the latter, one inhumation and one cremation were found in the same burial. Such a co-occurrence of inhumation and cremation burial rites on one site marks a peculiarity of the cluster region, which increases towards the south-east and describes regional differences in burial customs. While inhumation rites were predominant in the north-eastern part of the cluster region (*120*, *298*), there is a recognisable increase of cremation burials towards the south-east, leading to a prevalence of cremation burials among Bell Beaker communities of the so-called Csepel group. In this area along the Middle Danube, both scattered and urned cremations were more frequent than inhumations (*295*, *304*). A further regional peculiarity is the presence of cenotaphs—symbolic graves containing grave goods, but no human remains. At Szigetszentmiklós-Felső Ürge-hegyi dűlő, 29 such features were found, with similar makeups as on other Bell Beaker cemeteries in the area (*304*). This feature category, however, was not considered for the analysis.

The grave goods of the Eastern Bell Beaker group predominantly consist of vessels, including bell beakers, bowls, cups, jugs, and plates, known as *'Begleitkeramik'* (*47*). Typical tools include flint flakes, blades, as well as copper items like daggers and awls. Similar to other European regions, flint arrowheads and stone wristguards are typically seen as part of archery equipment. Ornaments include amber or bone beads and pendants, V-perforated buttons, and, more rarely, items made of copper, silver, or gold, such as spiral rings or metal sheets. Additionally, animal bones, likely representing food offerings, are frequently found (*120*, *296*, *298*, *303*, *304*, *307*).

In terms of absolute chronology, different timeframes have been proposed for various regions within the Eastern Bell Beaker group. While absolute chronologies for Lesser Poland propose Bell Beaker burial customs for the time between 2400-2250/2200 cal BC (*297*), the Iwno group in more northernly Kujavia has been dated to 2210-1880 cal BC (*298*). In Moravia, the Bell Beaker phenomenon is dated to 2500-2200 cal BC (*307*), while the Csepel group in Hungary is dated to c. 2550-1990 cal BC (*311*). According to our analysis, which merges observations of the different Eastern Bell Beaker provinces into a single model, the earliest Bell Beaker burial events in the region are OLE-estimated to have emerged from c. 2476 cal BC onwards. The bimodal probability distribution of the KDE model shows a major probability peak between 2400–2150 cal BC, followed by a decline that ends with the discontinuation of Bell Beaker burial rites around c. 1930 cal BC, as indicated by the OLE model. These findings largely align with previous proposals on the absolute chronology.

Of the modelled burial events, 12 individuals were previously analysed in genetic studies (*4, 124*). Especially the study by Olalde *et al.* (*4*) observed high heterogeneity in genetic ancestry at a regional scale, pointing out that roughly contemporaneous Bell Beaker-associated individuals at the Szigetszentmiklós cemetery, for instance, revealed different proportions of 'steppe ancestry' (ranging from 0% to 75%). This indicates that even at a local scale, people associated with the Bell Beaker complex had diverse ancestries, irrelevant to the practised burial rite(*4*).

Criteria for the Selection of Radiocarbon Dates and Radiocarbon Modelling

Authors:

Quentin P.J. Bourgeois, Florian Helmecke, Erik J. Kroon, S. Louise Olerud*

*Faculty of Archaeology, Leiden University, Einsteinweg 2, 2333CC Leiden, the Netherlands

To ensure the quality of our datasets, we first collected and critically assessed all reported and published radiocarbon dates associated with Corded Ware and Bell Beaker burials. We found that a significant amount of radiocarbon dates are poorly associated with the event that they are expected to date (in our case, the burial). After this careful review more than 646 radiocarbon dates generally reported in the literature had to be discarded. In the document below we expand on our selection criteria, and we discuss the effects of the calibration curve on the modeling we performed.

1. *Data selection*

After the large radiocarbon dating programs of the 1990s and early 2000s, the recent surge of archaeogenetic studies has contributed to a considerable increase in radiocarbon dates from the third millennium BCE. At the same time, radiocarbon date calibration and modelling techniques have advanced. The latest IntCal20 curve is an updated and considerably more robust calibration curve than earlier calibration curves (*59*). Although it does not bring about drastic changes for the third millennium BCE compared to the previous IntCal13 curve, it provides an update for the calibration of radiocarbon dates (*312*). Most narratives about regional spatio-temporal developments during the third millennium BCE rely on often relatively few samples, with sometimes extremely high standard deviations, which were taken until ca. 1999/2000 and calibrated along the IntCal98 curve, thus representing a state of knowledge state from the early 2000s. An update on available radiocarbon dates and their calibration along the most recent curve is therefore necessary in order to revisit established models about the chronology of the third millennium BCE. In our study, we perform the first, continent-wide survey of radiocarbon dates for the appearance of Corded Ware and Bell Beaker burial rites. During this survey, we critically assessed the quality of the dates in order to make a reliable selection of relevant dates. Here, we will describe the choices made behind this selection as well as the process of our rigorous quality control.

2. *Archaeological context of samples*

For our assessment of emerging Corded Ware and Bell Beaker communities, we focused on one of the most denoting contexts for both archaeological cultures: their funerary contexts. Indeed, the Corded Ware and Bell Beaker burial rites are now understood to be the denominating factor behind these archaeological cultures. Furholt (*313*) even coins the term 'Single Grave Burial Ritual Complex' (SGBR) to describe the polythetic package of burial rituals that appears during the Corded Ware period and remains the predominant way of burying the dead until c. 1400 BCE. The SGBR consists of single inhumation burials, buried in a crouched flexed position according to strict, gendered orientation rules, together with a standardised set of grave goods. For the Corded Ware, these grave goods typically consist of Corded Ware beakers and amphorae, stone and flint axes, flint flakes and blades/daggers, and amber and bone ornaments (*33, 54*). For the Bell Beaker, the typical grave set consists of Bell Beaker beakers and accompanying pottery (*Begleitkeramik*), archery equipment (flint arrowheads, stone wristguards, arrow shaft smoothers), copper daggers, cushion stones and amber and bone ornaments (*54, 314*).

Due to the characterising nature of the funerary context for the Corded Ware and Bell Beaker phenomena, we only selected dates from burials. Settlement dates have been excluded as the cultural attribution of settlements is often problematic due to their longevity and complex stratigraphical nature. Furthermore the cultural attribution of settlements is often disputed, f.e. so-called Corded Ware settlements in the Dutch wetlands only contain low amounts of Corded Ware pottery and lack the classical burials often associated with Corded Ware (*42*). Similarly, early Bell Beaker settlement in Portugal are often dated from contexts indirectly associated with Bell Beaker pottery (*237*).

Dates have only been selected from burials which obey the typical Corded Ware and Bell Beaker burial rite. Therefore, some dates from human remains sampled for genetic sequencing, have been excluded because they were not from clear burial contexts (e.g. Thames skulls (*4*)). As we have not excavated and analysed the graves ourselves, we are dependent on local specialists for the correct cultural identification of a burial. However, we have in general excluded dates from burials attributed to Corded Ware/Bell Beaker but without (diagnostic) grave goods. Indeed, in our selection, diagnostic grave goods weigh heavier than e.g. body positioning and grave orientation, since the latter two are extremely prone to change and local idiosyncrasy (*33*).

Dates from burials containing more than one individual, i.e. non-single graves, have been included, but only when they can be assigned unequivocally to Corded Ware/Bell Beaker burial practices. Thus, burials and depositions of Corded Ware ceramics in older megalithic graves, which may have appeared very early in a region, (f.e. Danish Isles, NE Germany, NL) have been excluded as they are not unambiguously attributable to Corded Ware. The Corded Ware/Bell Beaker finds are often intermixed with other material that is attributable to other archaeological cultures, and discrete burials are often not distinguishable within these open contexts. This exclusion, however, does create knowledge gaps and/or a selection bias for certain regions: Switzerland, northeastern Germany (Mecklenburg-Vorpommern), the Danish Isles, the Scandinavian Peninsula, the Netherlands, western France and Iberia. We consider this bias permittable, due to our focus on the spread of typical Corded Ware/Bell Beaker burial rites. The deposition of individuals in already existing megalithic tombs can be seen as a 'community anchoring' mechanism (*315*), by either keeping up locally established rites or potentially even framing the new in the symbolism of the old, in contrast to the novel SGBR expressed through the typical Corded Ware/Bell Beaker burial rites.

Lastly, radiocarbon dates should be directly associated with the actual grave itself. In other words, the sample must be taken from within the burial pit. Due to this criterion, numerous dates from outside the grave, for instance the burial mound cover or a post from the surrounding post circle, have been excluded, as no direct association between the radiocarbon date and the burial event can be established.

Next to our rigorous control of the archaeological context of the dates, we reviewed the quality of the radiocarbon dates themselves. Wherever possible, we collected dates from short-lived sample material directly associated with the buried individual. Ideally, this consisted of collagen from human bone. Depending on the context, however, other materials were also considered.

A crucial aspect was the avoidance of samples affected by the freshwater reservoir effect (FRE). For example, in the case of the Corded Ware burial from Ząbie (Poland), the date from an antler belt plate with a 'terrestrial signal' was preferred over the date derived from the buried individual, which was determined to be affected by FRE due to a highly aquatic diet (*41, 97, 98*). In other instances, significant discrepancies in dates from the same context were treated as potential indicators of FRE, leading to the exclusion of samples wherever possible. This is exemplified by the Corded Ware multiple burial from Bolshnevo 3 (*79*), where one of three radiocarbon dates—taken from human bone material of adult individual BOL001—produced a significantly older result (UBA-41613: 4005±39 BP) compared to two other samples, one from charcoal (GIN-5240: 3960±130 BP; Krenke, 2019) and the other from human bone material of the second adult, BOL002 (UBA-41614: 3876±36 BP). Although both individuals belong to the same burial event, for which no confirmed FRE is yet documented but only assumed (*87*), we opted to consider only the younger date from the second individual's bone sample.

It is important to note, however, that the full impact of FRE on large portions of the sample set remains to be thoroughly investigated (*41, 87*). Generally, we have excluded dates for which there are known reservoir effects. However, we could only rely on published δ13C and δ15N values, and if the values were not published we had to assume no FRE effect was found.

In other cases, samples from human bone were simply not available due to the poor preservation conditions in some regions, such as in large parts of the Netherlands and Denmark. In these regions, radiocarbon dating is mostly based on charcoal. Although there are many issues regarding charcoal samples for radiocarbon dating (*28, 316*), such as a potential old wood effect, we have decided to include these lesser quality dates nevertheless, in order to be able to incorporate these crucial regions in our study. Dates from charcoal have only been entered on the condition that the samples were either from short-lived charred material (such as twigs) or from large concentrations of charcoal within the grave (such as a charred coffin). Dates from small specks of charcoal in the fill of the grave or charcoal from the surface under the grave were thus excluded, as the association of these samples to the burial event is unsure.

Lastly, we devised a protocol for multiple dates from one grave, which is a common occurrence. In the case of a more recent AMS date replacing an older date, we selected the most recent date. In the case of multiple dates from the same individual (human bone or dentine), we combined the dates using the R_Combine function in OxCal. In the case of multiple dates from different individuals (human bone or dentine) in a multiple grave, we did not combine the dates, but made a decision on which date to include. Typically, we included the youngest date, except if the dated individual was an infant, whose radiocarbon-detected age can be off-set through breastfeeding and weaning, which both enrich the carbon and nitrogen content of the bone sample (*317, 318*). In the case of multiple charcoal dates from the same grave, we included the youngest date in order to diminish any potential old wood effect.

A consequence of these stringent criteria is that we had to reject upwards of 40% of all reported radiocarbon-dated Corded Ware or Bell Beaker burial events. This rejection of dates is not uniform though, with the rejection rate in some clusters reaching more than 80% of burial events. This is particularly true for regions where burial in (open) caves or megaliths was common. Open-source databases like Radon B, while valuable as initial resources, are substantially compromised by inconsistencies, such as frequent misattributions, and insufficiently robust context data. This is most pertinent when the association between the dated sample and the intended context is unclear.

Consequently, a qualitative assessment and recalibration of each date relative to the specific research question are still required to enhance data reliability.

3. *Assessing the effect of the calibration curve on the KDE models.*

We use the OXCAL KDE_Model to calculate the probability densities of radiocarbon dated Corded Ware and Bell Beaker burial events across Europe on the basis of 453 Corded Ware and 514 Bell Beaker burial events. The probability distributions generated by the KDE_Model are less affected by platforms and inclines on the IntCal20 calibration curve (*59*) than the commonly used Summed Probability Distributions (SPDs) and Summed Calibrated Probability Distributions (SCPDs). However, discerning the impact of plateaus and inclines remains crucial (*29, 61*).

To examine for the impact of the calibration curve on the KDE, we adapted the modelling approach from Capuzzo *et al.* (2023). We simulate 968 radiocarbon dates with a 2-sigma range of 30 years, evenly distributed across the range 3500-1250 BCE in OxCal v4.4.4 (*58*). This temporal range and the number of dates corresponds to the empirical data (see Fig. 3-4). The code for this simulation in Oxcal v4.4 is appended below. Output of the model in OxCal is shown in Supplementary Figure 1. The uniform temporal distribution of these simulated data are not meant to approximate the degree of temporal clustering seen in the archaeological data. Instead, it shows a hypothetical situation in which the probability of a burial event in any given year is constant and any peak is caused by the shape of the calibration curve. Therefore, a comparison of the simulated KDE against the empirical KDEs highlights the areas in which the probability densities of burial events may have been affected by the shape of the calibration curve.

The outcome of the simulation shows no major peaks in the posterior probability distribution, which suggests that the plateaus and steps in the calibration curve have minor impact on the probability distributions in Fig. 3-4. Therefore, we conclude the KDE for Corded Ware and Bell Beaker burials are an accurate representation of a pattern in the empirical data (*61*). Minor fluctuations are visible around the edges of the KDE. These are edge effects which result from the parameters for the simulation (*29*). However, the use of OLE to infer the start and end dates should counteract these edge effects, as OLE is built to deal with sparse data and works from the spacing of the individual points rather than the KDE (*30*).

OxCal code estimation calibration effect

```
Plot()
 {
  KDE_Model()
  {
   // define and initialise the variable
   var(a);
   var(b);
   var(n);
   var(i);
   a=3500;
   // start date in BC
```

```
  b=1250;
  // end date in BC
  n=967;
  // number of dates in simulation
  i=0;
  while(i<n)
  {
   // calibrate the date
   R_Simulate(BC(a+i*(b-a)/n),30);
   i=i+1;
  };
 };
};
```

4. *Regional KDE Models (Oxcal v.4.4.4)*

Corded Ware regions:

1. Upper Volga

```
Plot()
 {
  KDE_Model("Upper Volga Region")
  {
   R_Date("UBA-41637",4036,32);
   R_Date("UBA-41622",4033,29);
   R_Date("UBA-41635",4031,36);
   R_Date("UBA-41623",3763,30);
   R_Date("UBA-41634",3931,35);
   R_Date("UBA-41621",4092,35);
   R_Date("UBA-41618",4083,33);
   R_Date("UBA-41619",4036,37);
   R_Date("KIA-50462",3975,35);
```

```
  R_Date("UBA-41615",3956,34);
  R_Date("UBA-41614",3876,36);
  R_Date("UBA-41640",4153,33);
  R_Date("UBA-41629",4148,49);
  R_Date("UBA-41628",4141,33);
  R_Date("UBA-41626",4100,34);
  R_Date("UBA-41630",4047,54);
  R_Date("UBA-41631",4039,33);
  R_Date("UBA-41617",4037,32);
  R_Date("UBA-41625",4036,40);
  R_Date("UBA-41641",4002,54);
  R_Date("UBA-41639",3987,29);
  R_Date("UBA-41627",3972,54);
  R_Date("UBA-41616",3968,32);
  R_Date("UBA-41638",3943,41);
  R_Date("UGAMS-63145",3700,25);
  R_Date("UGAMS-63143",3920,25);
  R_Date("UGAMS-63142",3980,25);
  R_Date("UGAMS-63141",3900,25);
  R_Date("UGAMS-63144",3850,25);
 };
};
```

2. Eastern Baltics

```
Plot()
 {
  KDE_Model("Eastern Baltics")
  {
  R_Date("OxA-X-2417-15",4187,31);
  R_Date("Poz-89314",4005,35);
  R_Date("UBA-38464",4034,41);
  R_Date("Poz-10827",4090,35);
  R_Date("Poz-10803",4035,35);
  R_Date("UBA-29064",3969,32);
  R_Date("R-combine",3992,20);
  R_Date("Poz-15499",3805,35);
  R_Date("Hela-3429",4216,28);
```

```
  R_Date("Hela-4083",4181,60);
  R_Date("Ua-19801",4285,75);
  R_Date("Ua-19811",4280,60);
  R_Date("Ua-15545",4190,90);
  R_Date("Ua-19802",4165,60);
  R_Date("OxA-5936",4280,75);
  R_Date("Poz-61584",4030,30);
  R_Date("Poz-66923",4025,30);
  R_Date("Poz-64678",3955,30);
  R_Date("Ki-5717",3775,40);
  R_Date("Poz-87715",3980,35);
  R_Date("Poz-91682",4010,35);
  R_Date("Poz-91680",3970,35);
 };
};
```

3. Upper Bug-Dniester

```
Plot()
 {
  KDE_Model("Upper Bug-Dniester region")
  {
   R_Date("Poz-9587",4145,35);
   R_Date("Poz-9450",3915,35);
   R_Date("Poz-134519",4075,35);
   R_Date("Poz-25614",4045,35);
   R_Date("Poz-73135",4045,35);
   R_Date("Poz-134518",4005,35);
   R_Date("Poz-25616",3940,35);
   R_Date("Poz-134515",3975,35);
   R_Date("Poz-90760",3925,35);
   R_Date("Poz-134516",3910,35);
   R_Date("Poz-90899",3875,35);
   R_Date("Poz-90897",3865,35);
   R_Date("Poz-90898",3865,35);
   R_Date("Poz-90896",3825,35);
   R_Date("Poz-91069",3790,35);
   R_Date("R-combine",3981,26);
   R_Date("Ki-6889",3995,55);
```

```
  R_Date("Ki-6894",4020,55);
  R_Date("Ki-8955",3845,50);
  R_Date("Ki-8952",3850,60);
 };
};
```

4. Upper Vistula-San

```
Plot()
 {
  KDE_Model("Upper Vistula-San")
  {
  R_Date("Gd-10397",4290,90);
  R_Date("Ki-7931",4160,50);
  R_Date("Ki-5065",4160,50);
  R_Date("Ki-6755",4155,50);
  R_Date("Ki-7948",4150,80);
  R_Date("Poz-9451",4115,30);
  R_Date("Ki-5833",4105,35);
  R_Date("Ki-7930",4080,50);
  R_Date("Ki-5066",4080,55);
  R_Date("Ki-6758",4065,50);
  R_Date("Ki-5824",4060,50);
  R_Date("GrN-12515",4055,35);
  R_Date("Poz-60364",4055,30);
  R_Date("Poz-9454",4050,35);
  R_Date("Ki-5836",4035,40);
  R_Date("Poz-90877",4020,30);
  R_Date("Poz-90782",4020,35);
  R_Date("Ki-5119",4020,55);
  R_Date("Poz-9458",4015,35);
  R_Date("Poz-90771",4015,35);
  R_Date("Ki-5122",4010,65);
  R_Date("Poz-9582",4005,35);
  R_Date("AA-90116",4000,40);
  R_Date("GrN-12517",4000,30);
```

```
R_Date("Poz-90769",3995,35);
R_Date("Poz-90770",3995,35);
R_Date("Poz-90881",3985,35);
R_Date("Ki-5835",3980,40);
R_Date("Ki-5115",3970,70);
R_Date("Poz-80189",3960,30);
R_Date("Ki-5067",3960,60);
R_Date("Poz-90882",3955,35);
R_Date("Poz-9585",3955,35);
R_Date("Poz-9600",3950,40);
R_Date("Poz-90777",3950,35);
R_Date("Poz-90778",3950,35);
R_Date("Poz-27990",3940,40);
R_Date("Poz-54038",3940,35);
R_Date("Ki-5235",3940,60);
R_Date("Poz-90880",3935,35);
R_Date("Poz-90779",3935,30);
R_Date("Poz-90772",3935,35);
R_Date("Poz-27992",3930,40);
R_Date("Poz-90774",3930,30);
R_Date("Ki-5123",3930,60);
R_Date("Poz-9452",3930,35);
R_Date("GrN-12516",3925,30);
R_Date("R-combine",3913,23);
R_Date("Poz-52608",3915,25);
R_Date("Poz-90765",3910,30);
R_Date("Poz-27991",3910,35);
R_Date("Ki-5116",3910,50);
R_Date("Poz-90773",3910,35);
R_Date("Poz-9584",3905,35);
R_Date("Poz-90761",3905,35);
R_Date("Ki-5124",3900,55);
R_Date("Poz-58115",3895,30);
R_Date("Ki-6757",3895,55);
R_Date("Poz-9577",3895,30);
R_Date("Poz-49278",3895,35);
R_Date("Poz-90875",3890,35);
R_Date("Poz-90780",3890,35);
R_Date("Poz-90874",3890,35);
```

```
  R_Date("Poz-90775",3885,35);
  R_Date("Poz-9588",3885,35);
  R_Date("Poz-9583",3885,35);
  R_Date("Poz-90885",3880,35);
  R_Date("Poz-12580",3880,35);
  R_Date("Poz-90768",3875,35);
  R_Date("Poz-90876",3875,35);
  R_Date("Poz-9457",3870,35);
  R_Date("Poz-58114",3870,30);
  R_Date("Poz-54043",3870,35);
  R_Date("GrN-10745",3865,35);
  R_Date("Poz-59407",3860,35);
  R_Date("Poz-54041",3855,25);
  R_Date("Poz-9581",3840,35);
  R_Date("Poz-9579",3835,35);
 };
};
```

5. Upper Odra-Morava

```
Plot()
 {
  KDE_Model("Upper Odra-Morava")
  {
  R_Date("Erl-4726",3938,59);
  R_Date("Poz-14919",3890,35);
  R_Date("Poz-14924",3720,40);
  R_Date("Erl-4680",3643,49);
  R_Date("Erl-4727",3528,56);
  R_Date("KIA-34706",4235,30);
  R_Date("KIA-34711",4090,30);
  R_Date("KIA-34693",4075,35);
  R_Date("KIA-34696",4055,25);
  R_Date("KIA-34701",4050,25);
  R_Date("KIA-34704",4045,25);
  R_Date("KIA-34709",4035,30);
  R_Date("KIA-34702",4025,25);
  R_Date("KIA-34703",4000,25);
  R_Date("KIA-34705",4000,30);
```

```
  R_Date("KIA-34700",3965,25);
  R_Date("KIA-34695",3965,25);
  R_Date("KIA-34733",3955,30);
  R_Date("KIA-34732",3910,35);
  R_Date("KIA-34708",3900,25);
  R_Date("Poz-25936",4080,40);
  R_Date("GrN-20926",3960,40);
  R_Date("MAMS-30751",3896,22);
  R_Date("DeA-35010",4035,25);
  R_Date("Poz-98508",3850,40);
  R_Date("DeA-16085",3919,30);
  R_Date("DeA-16086",4067,30);
  R_Date("BETA-487621",4100,30);
  R_Date("MAMS-52820",3988,20);
  R_Date("MAMS-52833",3771,23);
  R_Date("DeA-19323",3920,38);
  R_Date("DeA-36656",3979,22);
  R_Date("DeA-35009",3951,26);
  R_Date("DeA-26662",4043,27);
  R_Date("MAMS-48835",4003,25);
  R_Date("MAMS-34184",3951,26);
  R_Date("MAMS-34183",3986,26);
  R_Date("MAMS-34182",3988,27);
  R_Date("DeA-33257",4004,26);
  R_Date("R-combine",4016,19);
 };
};
```

6. Bohemian Basin

```
Plot()
 {
 KDE_Model("Bohemian Basin")
  {
  R_Date("MAMS-45793",4314,25);
  R_Date("BRAMS-2959",4016,26);
  R_Date("R-combine",4250,20);
  R_Date("CRL-9182",4261,118);
  R_Date("CRL-19317",3989,44);
  R_Date("MAMS-30795",4259,23);
```

```
R_Date("CRL-9200",4256,82);
R_Date("CRL-9198",4247,80);
R_Date("CRL-9203",4241,84);
R_Date("CRL-9192",4226,95);
R_Date("MAMS-30759",4212,21);
R_Date("MAMS-30758",4196,21);
R_Date("MAMS-45792",4177,25);
R_Date("R-combine",4166,25);
R_Date("MAMS-45798",4171,26);
R_Date("MAMS-45787",4147,24);
R_Date("MAMS-45789",4130,24);
R_Date("Poz-86648",4110,35);
R_Date("MAMS-45796",4105,25);
R_Date("MAMS-45794",4093,24);
R_Date("MAMS-45791",4081,25);
R_Date("R-combine",4067,24);
R_Date("MAMS-38481",4055,29);
R_Date("MAMS-38483",4048,26);
R_Date("MAMS-38472",4027,25);
R_Date("MAMS-41374",4017,25);
R_Date("MAMS-44709",3987,25);
R_Date("MAMS-30761",3966,25);
R_Date("MAMS-45788",3947,24);
R_Date("MAMS-30760",3941,21);
R_Date("PSUAMS-3887",3935,25);
R_Date("MAMS-46362",3918,21);
R_Date("MAMS-41377",3917,26);
R_Date("MAMS-38473",3913,26);
R_Date("MAMS-38474",3902,28);
R_Date("MAMS-41375",3898,26);
R_Date("PSUAMS-3888",3885,25);
R_Date("MAMS-41378",3853,27);
R_Date("PSUAMS-4026",3850,25);
R_Date("MAMS-44710",4127,25);
R_Date("GrN-9376",4015,30);
R_Date("CRL-17484",3981,22);
R_Date("GrN-9379",3940,35);
R_Date("GrN-9481",3935,35);
R_Date("GrN-9482",3860,35);
```

```
  R_Date("KIA-11798",3854,39);
 };
};
```

7. Northeast European Plain

```
Plot()
 {
  KDE_Model("Northeast European Plain")
  {
   R_Date("Bln-3992",3670,50);
   R_Date("Bln-4399",3892,40);
   R_Date("Hd-15773",3963,23);
   R_Date("R-combine",4163,25);
   R_Date("R-combine",4148,30);
   R_Date("Poz - 36250",4160,35);
   R_Date("Poz-75446",4000,30);
   R_Date("Poz-33427",4140,40);
   R_Date("OxA-26755",4086,29);
   R_Date("Poz-77329",3950,35);
   R_Date("Ki-6242",3940,40);
   R_Date("R-combine",3877,23);
   R_Date("Ki-6881",3865,45);
   R_Date("Gd-1684",3840,50);
   R_Date("Poz-16596",3830,35);
   R_Date("Poz-20655",3730,70);
   R_Date("MAMS-13338",3927,26);
   R_Date("Poz-74635",4025,35);
   R_Date("Poz-85608",3775,35);
   R_Date("KIA-28329",3920,30);
  };
 };
```

8. Scandinavian Peninsula

```
Plot()
 {
  KDE_Model("Scandinavian Peninsula")
```

```
 {
  R_Date("Beta - 423305",4020,30);
  R_Date("Ua-37496",4135,45);
  R_Date("Ua-19469",4080,65);
  R_Date("Ua-37497",4075,40);
  R_Date("Ua-37494",3990,40);
  R_Date("UBA-39934",3987,34);
  R_Date("Ua-36982",3975,35);
  R_Date("OxA-39287",3955,20);
  R_Date("Lu-474",3930,80);
  R_Date("Ua-19468",3890,55);
  R_Date("Ua-33978",3755,35);
  R_Date("Ua-5361",3730,50);
  R_Date("R-combine",4031,27);
 };
};
```

9. Middle Elbe-Saale

```
Plot()
 {
 KDE_Model("Middle Elbe-Saale")
  {
  R_Date("KI-4139",3960,85);
  R_Date("KN-4886",3728,48);
  R_Date("KI-4140",4040,45);
  R_Date("KI-4141",3939,45);
  R_Date("KIA-40716",4015,35);
  R_Date("KN-4887",3868,40);
  R_Date("KN-4888",3789,59);
  R_Date("KN-4889",3648,52);
  R_Date("KN-4890",3700,60);
  R_Date("KIA-162",4080,20);
  R_Date("KN-4891",3875,40);
  R_Date("Erl-7807",4032,56);
  R_Date("Hd-18963",3830,18);
  R_Date("KN-4892",3834,39);
  R_Date("KN-4866",3991,54);
  R_Date("KI-4142/KIA-2689",3890,95);
```

```
R_Date("KI-4143",4040,45);
R_Date("KI-4144",4060,95);
R_Date("KI-4145",3840,50);
R_Date("MAMS 21488",4000,31);
R_Date("Erl7779",3967,57);
R_Date("KIA-34264",4078,30);
R_Date("KIA-27850",4073,27);
R_Date("KIA-27852",4053,27);
R_Date("KIA-27878",3969,29);
R_Date("KI-4146",3760,45);
R_Date("KI-4147",3920,50);
R_Date("KIA-354",4150,30);
R_Date("KN-4893",3876,52);
R_Date("KI-4149/KIA-2968",3760,30);
R_Date("KI-4150",3770,50);
R_Date("KI-4148",3740,55);
R_Date("Hd-19048",3942,24);
R_Date("HD-19634",4024,27);
R_Date("KIA32304",4135,28);
R_Date("MAMS-111771",4070,28);
R_Date("MAMS-18152",4047,19);
R_Date("KI-4153",3890,35);
R_Date("MAMS 19530",4096,18);
R_Date("KI-4154",3800,45);
R_Date("KI-4155",3890,35);
R_Date("KI-4156",3850,35);
R_Date("KI-4157",3930,45);
R_Date("KI-4158",3960,40);
R_Date("Erl-4195",4013,56);
R_Date("Erl-4193",3690,65);
R_Date("KI-4151",3910,45);
R_Date("MAMS-17070",4032,20);
R_Date("MAMS-17064",3999,20);
R_Date("KIA-40715",3955,35);
R_Date("KIA-40717",3965,30);
R_Date("MAMS-17173",4027,20);
R_Date("MAMS-111739",4017,26);
R_Date("MAMS-17087",4006,28);
R_Date("MAMS-17181",4034,20);
```

```
  R_Date("MAMS-17073",3989,20);
  R_Date("MAMS-21691",4021,25);
  R_Date("MAMS-17082",4015,23);
  R_Date("KIA-41736",4203,32);
  R_Date("Erl-8629",3926,57);
  R_Date("Erl-10966",4239,59);
  R_Date("MAMS-111747",4022,25);
  R_Date("MAMS-111775",3988,29);
  R_Date("KIA 38983",4115,25);
  R_Date("KIA 38984",4305,30);
  R_Date("MAMS 21487",3927,37);
  R_Date("KIA34261",4113,25);
  R_Date("KIA29116",4078,31);
  R_Date("KIA29118",4009,32);
  R_Date("KIA26664",4027,34);
  R_Date("KIA29551",4127,25);
  R_Date("Erl7042",3792,50);
  R_Date("KIA 38724",4100,30);
  R_Date("KIA 38725",4095,30);
  R_Date("MAMS-17071",4033,19);
  R_Date("MAMS-17270",3879,24);
  R_Date("MAMS-21675",4064,29);
  R_Date("R-combine",4028,25);
  R_Date("R-combine",3994,27);
  R_Date("KIA-23647",4056,29);
  R_Date("Erl 10901",4216,49);
  R_Date("MAMS-14075",3878,24);
  R_Date("KIA39547",3987,38);
  R_Date("KIA39548",4073,33);
  R_Date("KIA39549",4163,27);
  R_Date("KIA39550",3829,26);
  R_Date("KIA39552",4095,26);
  R_Date("KIA39553",4006,26);
 };
};
```

10. Main-Danube

```
Plot()
```

```
{
KDE_Model("Main-Danube")
{
R_Date("HD-14008-13651",4010,45);
R_Date("HD-14009-13748",4015,25);
R_Date("HD-14010-13871",4050,25);
R_Date("HD-14011-13896",3905,35);
R_Date("Hd-14232-13797",4155,40);
R_Date("Hd-19564",4057,24);
R_Date("Hd-19669",4044,29);
R_Date("Hv-9225",3960,60);
R_Date("KN-2164",3860,90);
R_Date("KN-3308",3910,81);
R_Date("KN-3309",3860,84);
R_Date("KN-3310",3720,84);
R_Date("KN-3312",3700,78);
R_Date("KN-3313",4190,84);
R_Date("R-combine",4097,13);
R_Date("MAMS 23728",4234,24);
R_Date("MAMS 23729",4155,23);
R_Date("UBA-27948",4124,31);
R_Date("UBA-27950",4015,38);
R_Date("OxA-32091",4143,29);
R_Date("MAMS 18499",4055,18);
R_Date("HD-22060",4131,23);
R_Date("OxA-31175",4005,32);
R_Date("OxA-31062",4032,30);
R_Date("OxA-31063",4004,29);
R_Date("OxA-31064",3965,31);
R_Date("OxA-31065",4023,31);
R_Date("OxA-31066",4001,33);
R_Date("OxA-31067",4013,30);
R_Date("MAMS-19696",4071,22);
R_Date("Erl-16793",4212,42);
R_Date("Erl-17862",4083,52);
R_Date("R-combine",4232,33);
R_Date("OxA-32092",4120,29);
R_Date("UBA-27949",3968,33);
R_Date("OxA-31805",4043,27);
```

```
   R_Date("OxA-31806",4047,27);
   R_Date("OxA-31807",3992,28);
   R_Date("OxA-31894",4048,27);
   R_Date("OxA-31879",4047,27);
   R_Date("OxA-31809",3996,28);
   R_Date("OxA-31811",4050,28);
   R_Date("OxA-31947",4080,33);
   R_Date("OxA-31948",4016,31);
   R_Date("OxA-31949",3994,32);
   R_Date("OxA-31951",3971,31);
   R_Date("OxA-31952",4003,32);
   R_Date("OxA-31988",3979,30);
   R_Date("OxA-31989",4064,30);
   R_Date("Hv-9767",3715,40);
   R_Date("KN-2400",3800,60);
  };
 };
```

11. North-West European Plain

```
Plot()
 {
  KDE_Model("North-West European Plain")
  {
   R_Date("K-4533",4010,60);
   R_Date("GrN-6644",4160,30);
   R_Date("GrN-7802",4140,50);
   R_Date("GrA-41646",4075,30);
   R_Date("GrA-14965",4065,45);
   R_Date("GrA-12384",4005,60);
   R_Date("GrN-6349",3945,40);
   R_Date("GrN-6368",3935,35);
   R_Date("K-2966",4190,90);
   R_Date("K-1582",4150,100);
   R_Date("AAR-7028",4140,50);
   R_Date("K-2499",4140,70);
   R_Date("K-2933",4140,85);
   R_Date("AAR-4992",4135,45);
   R_Date("K-2185",4100,100);
```

```
  R_Date("K-1843",4080,100);
  R_Date("K-3229",4060,65);
  R_Date("K-2298",4050,100);
  R_Date("K-4705",4050,60);
  R_Date("K-2442",4040,100);
  R_Date("K-3248",4040,85);
  R_Date("K-3245",4020,85);
  R_Date("K-3628",4010,85);
  R_Date("K-1925",4000,100);
  R_Date("K-3625",4000,85);
  R_Date("K-3232",3990,85);
  R_Date("K-4270",3990,60);
  R_Date("K-2181",3980,100);
  R_Date("K-2672",3980,65);
  R_Date("K-2807",3980,90);
  R_Date("K-3472",3980,85);
  R_Date("AAR-6114",3975,55);
  R_Date("K-2711",3970,90);
  R_Date("UBA-36754",3950,31);
  R_Date("K-2712",3940,80);
  R_Date("K-4023",3920,85);
  R_Date("K-1831",3910,100);
  R_Date("K-4022",3910,85);
  R_Date("KIA-40269",4225,30);
  R_Date("GrN-8008",4215,40);
  R_Date("Hv. 22262",4185,55);
  R_Date("Hv. 22263",4105,60);
  R_Date("Hv. 22264",4160,55);
  R_Date("GrN-7518",4170,45);
  R_Date("AAR-10038",3974,46);
  R_Date("K-2118",4150,100);
 };
};
```

Bell Beaker regions

1. West Iberia

Plot()

```
{
 KDE_Model("West Iberia")
 {
  R_Date("Beta- 330091",3740,30);
  R_Date("SANU-53513",3871,35);
  R_Date("SANU-53512",3805,35);
  R_Date("SANU-53514",3772,32);
  R_Date("SANU-53101",3940,25);
  R_Date("SANU-53106",3905,25);
  R_Date("SANU-53037",3795,26);
  R_Date("Beta-496305",3720,30);
  R_Date("Sac-2790",3900,45);
  R_Date("Sac-2791",3940,50);
  R_Date("Wk-45324",3965,18);
  R_Date("Wk-45325",3875,19);
  R_Date("Beta-178468",3790,40);
  R_Date("Beta-178467",3830,40);
  R_Date("Wk52161",3739,17);
  R_Date("Beta-194027",3900,40);
  R_Date("16B0304",3550,30);
  R_Date("Beta-467882",3430,30);
  R_Date("GrN-10972",4100,60);
  R_Date("GrN-10971",3960,40);
  R_Date("GrN-10973",4000,35);
  R_Date("ICEN-1242",3940,45);
 };
};
```

2. Central and South-East Iberia

```
Plot()
 {
  KDE_Model("Central and South-East Iberia")
  {
   R_Date("Beta- 83083",3690,50);
   R_Date("UGA-15903",3870,30);
   R_Date("PSUAMS-2320",3875,20);
   R_Date("Ua-35021",3525,40);
   R_Date("Beta-184837",3650,40);
```

```
R_Date("PSUAMS-2120",3870,30);
R_Date("Rome-1687",3720,70);
R_Date("Beta-145275",3890,40);
R_Date("Beta-157732",3830,40);
R_Date("GX-29950",3650,40);
R_Date("Beta-157730",3810,40);
R_Date("Beta-157729",3790,40);
R_Date("Ua39420",3780,32);
R_Date("CSIC-970",3680,20);
R_Date("Ua- 43524",3917,33);
R_Date("R-combine 398",3805,27);
R_Date("GrM- 16341",3820,45);
R_Date("GrM-15295",3945,25);
R_Date("R-combine 401",3889,17);
R_Date("Ua-423526",3875,31);
R_Date("R-combine 403",3922,20);
R_Date("CNA- 4025",3918,33);
R_Date("GrM- 15291",3910,25);
R_Date("CNA-4023",3941,33);
R_Date("GrM- 15289",3905,20);
R_Date("Ua-40218",3825,37);
R_Date("Ua-40217",3781,36);
R_Date("GrM- 15290",3910,25);
R_Date("GrM-15296",3920,25);
R_Date("Ua-41491",3679,35);
R_Date("KIA- 18000",3862,28);
R_Date("KIA- 17999",3860,30);
R_Date("R-combine 415",3959,25);
R_Date("Beta- 229791",3920,40);
R_Date("Beta- 222443",3830,40);
R_Date("R-combine 419",3910,22);
R_Date("Beta - 471832",3850,30);
R_Date("Beta - 471834",3840,30);
R_Date("Beta - 473695",3830,30);
R_Date("Beta-471833",3870,30);
R_Date("R-combine 425",3830,22);
R_Date("CNA4435-1-1",3690,30);
R_Date("CNA4436-1-1",3880,30);
R_Date("MAMS-11826",3780,30);
```

```
  R_Date("MAMS-11827",3711,29);
  R_Date("MAMS-11828",3701,26);
  R_Date("OxA- 2907",3730,65);
  R_Date("Poz-49174",3730,40);
 };
};
```

3. North-West Mediterranean

```
Plot()
{
 KDE_Model("North-West Mediterranean ")
 {
  R_Date("Tuc-20951",4060,65);
  R_Date("Ly-6650",3870,150);
  R_Date("BM 2365",4060,60);
  R_Date("LTL- 13808A",3764,45);
  R_Date("Poz- 28213",3905,35);
  R_Date("Poz-28212",4100,35);
  R_Date("UBAR-860",3870,45);
  R_Date("UBAR-1061",3890,45);
  R_Date("Beta-230406",4150,40);
  R_Date("ETH-12184",3800,60);
  R_Date("LTL-5035A",3671,40);
  R_Date("Erl-12190",3832,97);
 };
};
```

4. Seine-Loire River basins

```
Plot()
{
 KDE_Model("Seine-Loire River basins ")
 {
  R_Date("Poz-94880",4070,35);
  R_Date("Lyon-2065(Poz)",4030,35);
  R_Date("GrA-15981",4015,40);
  R_Date("Ly-10817",3995,45);
  R_Date("GrA-32767",3970,30);
```

```
  R_Date("GrA-37945",3955,35);
  R_Date("Poz-64782",3890,35);
  R_Date("R-combine 196",3823,28);
  R_Date("Ly-7681",3830,55);
  R_Date("Poz-64785",3805,30);
  R_Date("GrA-32811",3780,30);
  R_Date("GrA-17222",3780,40);
  R_Date("Gif-7456",3760,90);
  R_Date("Ly-7487",3700,50);
  R_Date("Lyon-3520(GrA)",3670,30);
  R_Date("PSUAMS-9404",3825,25);
  R_Date("Lyon-8255 (Oxa)",3845,35);
 };
};
```

5. Southern Britain

```
Plot()
 {
  KDE_Model("Southern Britain")
  {
  R_Date("OxA-1875",3990,80);
  R_Date("R-combine 101",3819,28);
  R_Date("OxA-V-2271-35",3935,32);
  R_Date("OxA-V-2271-33",3921,30);
  R_Date("SUERC-31857",3905,30);
  R_Date("SUERC-26185",3905,30);
  R_Date("HAR-4426",3900,100);
  R_Date("OxA-13541",3895,32);
  R_Date("SUERC-26162",3885,30);
  R_Date("OxA-4356",3880,90);
  R_Date("NZA-29534",3878,20);
  R_Date("SUERC-26177",3875,30);
  R_Date("SUERC-26186",3875,30);
  R_Date("OxA-V-2228-45",3873,28);
  R_Date("R-combine 115",3881,25);
  R_Date("NZA-23745",3856,30);
  R_Date("SUERC-26158",3855,30);
  R_Date("SUERC-31859",3855,30);
```

```
R_Date("Wk-18733",3852,33);
R_Date("R-combine 120",3838,21);
R_Date("R-combine 121",3838,30);
R_Date("SUERC-31867",3840,30);
R_Date("OxA-V-2271-32",3838,28);
R_Date("NZA-32788",3835,25);
R_Date("OxA-V-2271-34",3834,29);
R_Date("SUERC-43376",3832,27);
R_Date("SUERC-26194",3830,30);
R_Date("OxA-13562",3829,38);
R_Date("SUERC-54823",3829,30);
R_Date("OxA-V-2197-54",3825,36);
R_Date("R-combine 131",3814,25);
R_Date("R-combine 132",3758,18);
R_Date("R-combine 133",3803,22);
R_Date("SUERC-26196",3800,30);
R_Date("SUERC-30814",3800,35);
R_Date("OxA-V-2226-45",3799,29);
R_Date("OxA-V-2199-44",3795,32);
R_Date("NZA-16624",3792,60);
R_Date("OxA-V-2271-36",3791,30);
R_Date("SUERC-26178",3790,30);
R_Date("SUERC-26167",3790,30);
R_Date("NZA-23746",3789,30);
R_Date("OxA-1073",3780,80);
R_Date("OxA-8729",3780,40);
R_Date("NZA-32485",3779,30);
R_Date("OxA-V-2197-50",3774,36);
R_Date("NZA-32495",3774,30);
R_Date("BM-2956",3770,35);
R_Date("SUERC-26198",3765,30);
R_Date("OxA-V-2199-37",3764,28);
R_Date("OxA-V-2228-44",3763,27);
R_Date("SUERC-74755",3760,30);
R_Date("BM-2518",3760,50);
R_Date("Poz-83404",3760,35);
R_Date("OxA-V-2199-18",3749,30);
R_Date("OxA-8868",3740,40);
R_Date("R-combine 157",3715,23);
```

```
   R_Date("NZA-32490",3734,25);
   R_Date("OxA-V-2228-40",3734,30);
   R_Date("OxA-V-2228-43",3732,30);
   R_Date("SUERC-43375",3728,24);
   R_Date("SUERC-49483",3722,31);
   R_Date("BM-2703",3720,50);
   R_Date("OxA-12132",3711,28);
   R_Date("OxA-24595",3703,28);
   R_Date("HAR-9245",3680,100);
   R_Date("HAR-340",3670,80);
   R_Date("NZA-32494",3664,30);
   R_Date("BM-2522",3660,50);
   R_Date("BM-2704",3650,50);
   R_Date("BM-2725",3630,60);
   R_Date("BM-2642",3630,50);
   R_Date("BM-2520",3630,60);
   R_Date("CAR-1193",3610,70);
   R_Date("NZA-22735",3601,40);
  };
 };
```

6. Northern Britain

```
Plot()
 {
  KDE_Model("Northern Britain")
  {
   R_Date("SUERC-49872",3874,32);
   R_Date("OxA-26255",3686,28);
   R_Date("R-combine 173",3623,39);
   R_Date("R-combine 490",3887,23);
   R_Date("SUERC-15119",3915,40);
   R_Date("OxA-V-2172-22",3910,33);
   R_Date("R-combine 493",3881,27);
   R_Date("GrA-29077",3865,40);
   R_Date("OxA-V-2243-40",3854,31);
   R_Date("GrA-26515",3850,40);
   R_Date("R-combine 497",3821,23);
   R_Date("OxA-V-2172-23",3845,32);
```

```
R_Date("GrA-29078",3845,40);
R_Date("OxA-V-2166-34",3835,33);
R_Date("R-combine 501",3828,29);
R_Date("OxA-V-2243-49",3834,29);
R_Date("OxA-V-2246-41",3833,28);
R_Date("R-combine 504",3824,21);
R_Date("OxA-V-2243-52",3829,29);
R_Date("SUERC-71005",3827,33);
R_Date("OxA-V-2167-42",3826,39);
R_Date("OxA-V-2168-42",3824,32);
R_Date("OxA-V-2243-57",3819,27);
R_Date("OxA-13213",3816,29);
R_Date("OxA-V-2228-39",3815,29);
R_Date("OxA-V-2243-41",3813,30);
R_Date("OxA-V-2243-51",3809,29);
R_Date("OxA-13514",3806,30);
R_Date("R-combine 515",3823,25);
R_Date("OxA-V-2172-31",3803,32);
R_Date("BM-2523",3800,50);
R_Date("OxA-13513",3797,31);
R_Date("OxA-V-2243-47",3795,28);
R_Date("OxA-13660",3794,26);
R_Date("OxA-V-2246-34",3785,26);
R_Date("BM-2515",3780,60);
R_Date("OxA-V-2243-54",3777,32);
R_Date("OxA-V-2166-44",3770,31);
R_Date("OxA-13098",3770,33);
R_Date("OxA-V-2172-14",3769,32);
R_Date("OxA-V-2243-45",3768,31);
R_Date("OxA-V-2243-46",3757,29);
R_Date("OxA-V-2166-42",3755,32);
R_Date("R-combine 530",3736,33);
R_Date("GrA-29079",3750,45);
R_Date("R-combine 532",3752,27);
R_Date("OxA-V-2166-41",3743,33);
R_Date("OxA-V-2166-46",3741,32);
R_Date("R-combine 535",3733,22);
R_Date("OxA-V-2172-19",3725,33);
R_Date("GU-2169",3710,70);
```

```
  R_Date("GrA-23982",3690,45);
  R_Date("AA-29066",3645,65);
  R_Date("SRR-553",3630,125);
  R_Date("BM-2512",3630,50);
  R_Date("OxA-13215",3605,37);
  R_Date("OxA-V-2172-27",3534,31);
 };
};
```

7. North-West European Plain

```
Plot()
 {
 KDE_Model("North-West European Plain")
  {
  R_Date("K-1451",4000,100);
  R_Date("K-3015",3840,85);
  R_Date("K-2963",3740,85);
  R_Date("K-5039",3730,60);
  R_Date("AAR-6273",3655,45);
  R_Date("K-5492",3640,75);
  R_Date("K-5491",3550,80);
  R_Date("GrA-14080",3810,40);
  R_Date("R-combine 447",3931,32);
  R_Date("GrA-13617",3910,50);
  R_Date("GrA-13602",3880,50);
  R_Date("GrA-41636",3875,35);
  R_Date("GrA-14840",3850,40);
  R_Date("GrA-14066",3840,35);
  R_Date("GrA-11264",3840,50);
  R_Date("SUERC-26372",3840,35);
  R_Date("GrN-6856",3835,55);
  R_Date("GrA-39655",3830,30);
  R_Date("GrA-14067",3830,35);
  R_Date("GrA-28356",3765,35);
  R_Date("GrN-6340",3760,35);
  R_Date("GrN-6711",3735,35);
  R_Date("GrN-6146",3725,35);
  R_Date("PSUAMS-7847",3700,25);
```

```
  R_Date("GrN-5131",3665,40);
 };
};
```

8. Upper-Rhine and Main-Danube region

```
Plot()
 {
  KDE_Model("Upper-Rhine and Main-Danube region")
  {
   R_Date("GrN-26995",4070,50);
   R_Date("MAMS-25935",4047,29);
   R_Date("GrA-15976",4045,40);
   R_Date("GrN-25476",4020,50);
   R_Date("GrN-20491",3940,90);
   R_Date("R-combine 190",3929,23);
   R_Date("GrN-32101",3925,40);
   R_Date("R-combine 192",3893,25);
   R_Date("Ly-3554/SacA-5448",3850,30);
   R_Date("GrN-44686",3830,35);
   R_Date("GrA-44685",3805,35);
   R_Date("Poz-68164",3795,35);
   R_Date("Ly-3555/SacA-5449",3755,30);
   R_Date("Hd-13664",3685,40);
   R_Date("HD-22049",3891,19);
   R_Date("HD-22050",3675,26);
   R_Date("HD-22070",3859,56);
   R_Date("MAMS 18935",3910,20);
   R_Date("HD-21795",3818,19);
   R_Date("GrN-9298",3830,35);
   R_Date("Hv-9436",3680,60);
   R_Date("R-combine 316",3831,22);
   R_Date("MAMS 18922",3810,19);
   R_Date("HD-22074",3777,44);
   R_Date("Hd-13652",3817,32);
   R_Date("MAMS 18913",3788,23);
   R_Date("MAMS 18921",3748,19);
   R_Date("R-combine 341",3794,13);
   R_Date("MAMS-18505",3632,24);
```

```
  R_Date("MAMS-29075",3870,30);
  R_Date("MAMS 18934",3840,20);
  R_Date("MAMS 18949",3819,24);
  R_Date("MAMS-18496",3857,18);
  R_Date("MAMS-18495",3828,17);
  R_Date("Beta-145712",3820,40);
  R_Date("Beta-145714",3680,40);
 };
};
```

9. Lower and Middle Elbe-Saale region

```
Plot()
 {
  KDE_Model("Lower and Middle Elbe-Saale region")
  {
  R_Date("Erl8538",3940,60);
  R_Date("Erl-8703",3876,46);
  R_Date("Erl8537",3858,57);
  R_Date("KIA27869",3825,30);
  R_Date("KIA-40713",3835,30);
  R_Date("KIA27952",3758,33);
  R_Date("KIA27951",3751,27);
  R_Date("KIA27950",3737,28);
  R_Date("Hd-19285",3871,22);
  R_Date("Hd-18738",3700,25);
  R_Date("KIA-41737",3843,33);
  R_Date("Hd-19165",3810,27);
  R_Date("KN-4865",3738,42);
  R_Date("Hd-19207",3758,21);
  R_Date("KIA-23646",3921,36);
  R_Date("Hd-18825",3748,25);
  R_Date("Hd-21777",4009,19);
  R_Date("Hd-21982",3823,16);
  R_Date("Erl-10957",3935,60);
  R_Date("Erl-10958",3812,59);
  R_Date("R-combine 233",3836,19);
  R_Date("KIA-35422",3788,26);
  R_Date("KIA-35423",3718,25);
```

```
R_Date("Hd-19265",3810,24);
R_Date("Erl 10902",4102,59);
R_Date("Erl 10908",4066,53);
R_Date("Erl 10906",4008,50);
R_Date("Erl 10903",3935,48);
R_Date("Erl 10905",3923,49);
R_Date("Erl 10907",3909,52);
R_Date("Erl 11021",3894,51);
R_Date("Erl 10909",3878,57);
R_Date("Erl 10910",3793,55);
R_Date("Erl 10911",3771,52);
R_Date("MAMS-16902",3814,27);
R_Date("MAMS-16903",3726,26);
R_Date("Hd-22154",3848,15);
R_Date("Hd-22134",3830,19);
R_Date("Hd-19333",3733,54);
R_Date("KIA35402",3853,31);
R_Date("KIA-35401",3787,30);
R_Date("KIA-35403",3685,40);
R_Date("BLN-1447",3810,61);
R_Date("KIA-32308",3928,28);
R_Date("KIA-32306",3914,26);
R_Date("KIA-32313",3826,23);
R_Date("KIA-32307",3816,34);
R_Date("MAMS-16774",3867,19);
R_Date("MAMS-16789",3863,20);
R_Date("MAMS-18153",3840,18);
R_Date("MAMS-16753",3836,21);
R_Date("MAMS-16757",3821,22);
R_Date("MAMS-16772",3815,18);
R_Date("MAMS-16773",3800,19);
R_Date("MAMS-16770",3795,18);
R_Date("MAMS-16769",3794,18);
R_Date("MAMS-16761",3782,22);
R_Date("MAMS-16760",3754,22);
R_Date("GrA-16981",3905,50);
R_Date("KIA-35417",3939,28);
R_Date("KIA-35416",3859,27);
R_Date("KIA-35415",3852,37);
```

```
R_Date("KIA-35418",3837,25);
R_Date("KIA-35414",3834,25);
R_Date("KIA-35419",3822,27);
R_Date("KIA-35421",3818,25);
R_Date("KIA-31431",3959,27);
R_Date("KIA-31426",3874,30);
R_Date("KIA-31437",3864,35);
R_Date("KIA-31429",3834,29);
R_Date("KIA-31440",3824,29);
R_Date("KIA-31439",3801,30);
R_Date("Erl8560",3871,61);
R_Date("Erl8558",3839,55);
R_Date("Erl8559",3782,56);
R_Date("Erl7038",3820,42);
R_Date("Erl-7041",3655,48);
R_Date("GrN-23312",3870,30);
R_Date("Erl8710",3953,47);
R_Date("Erl-8714",3885,45);
R_Date("Erl-8709",3876,46);
R_Date("Erl-8542",3871,57);
R_Date("MAMS-22819",3822,25);
R_Date("KIA-35427",3882,27);
R_Date("KIA-35426",3860,28);
R_Date("KIA-35425",3830,25);
R_Date("KIA-35428",3827,28);
R_Date("KIA-35430",3824,37);
R_Date("KIA-35429",3806,37);
R_Date("KIA-31740",3794,28);
R_Date("KIA-31747",3709,27);
R_Date("Bln-3914",3810,88);
R_Date("MAMS-16009",3905,29);
R_Date("MAMS-16026",3900,33);
R_Date("MAMS-17058",3870,28);
R_Date("MAMS-16035",3829,19);
R_Date("MAMS-16034",3825,18);
R_Date("MAMS-16027",3812,27);
R_Date("MAMS-16028",3762,22);
R_Date("R-combine 326",3812,25);
R_Date("KIA26663",3856,28);
```

```
  R_Date("BETA-437546",3840,30);
 };
};
```

10. Upper Elbe-Isar region

```
Plot()
 {
  KDE_Model("Upper Elbe-Isar region")
  {
   R_Date("MAMS-30800",3995,23);
   R_Date("MAMS-30797",3995,23);
   R_Date("MAMS-30801",3957,24);
   R_Date("MAMS-30791",3907,24);
   R_Date("MAMS-38482",3893,25);
   R_Date("MAMS-30784",3892,24);
   R_Date("PSUAMS-4349",3890,20);
   R_Date("MAMS-30796",3887,25);
   R_Date("KI-4448",3860,45);
   R_Date("MAMS-44707",3848,24);
   R_Date("MAMS-30763",3848,26);
   R_Date("PSUAMS-4346",3840,25);
   R_Date("MAMS-30798",3837,24);
   R_Date("PSUAMS-4347",3835,20);
   R_Date("R-combine 51",3836,18);
   R_Date("MAMS-30762",3815,21);
   R_Date("PSUAMS-4350",3810,20);
   R_Date("PSUAMS-2801",3805,20);
   R_Date("PSUAMS-2854",3795,20);
   R_Date("MAMS-30799",3795,25);
   R_Date("PSUAMS-2843",3790,20);
   R_Date("MAMS-30783",3787,24);
   R_Date("PSUAMS-2853",3785,20);
   R_Date("KIA-34738",3785,25);
   R_Date("MAMS-30782",3783,25);
   R_Date("PSUAMS-2847",3765,20);
   R_Date("PSUAMS-2848",3765,20);
   R_Date("PSUAMS-2852",3750,20);
   R_Date("PSUAMS-2844",3740,20);
```

```
  R_Date("Poz-84460",3740,35);
  R_Date("PSUAMS-2845",3730,20);
  R_Date("PSUAMS-2846",3700,20);
  R_Date("BRAMS-1218",3844,33);
  R_Date("BRAMS-1219",3825,26);
  R_Date("BRAMS-1215",3817,26);
  R_Date("BRAMS-1051",3813,20);
  R_Date("BRAMS-1217",3802,26);
  R_Date("Hd-19835",3848,34);
  R_Date("Hd-19797",3915,28);
  R_Date("Hd-19792",3848,24);
  R_Date("Poz-84553",3955,35);
 };
};
```

11. Oder-Vistula-Danube

```
Plot()
 {
  KDE_Model("Oder-Vistula-Danube")
  {
  R_Date("DeA-2876",3831,35);
  R_Date("DeA-2877",3874,33);
  R_Date("R-combine 3",3865,22);
  R_Date("DeA-2875",3845,36);
  R_Date("deb-13295",3600,50);
  R_Date("deb-13932",3690,50);
  R_Date("VERA-4720",3775,35);
  R_Date("VERA-4724",3940,35);
  R_Date("VERA-4726",3720,40);
  R_Date("VERA-4727",3815,35);
  R_Date("DeA-11507",3931,31);
  R_Date("Poz-83639",3850,35);
  R_Date("Poz-83640",3840,35);
  R_Date("R-combine 16",3833,27);
  R_Date("VERA-4748",3920,40);
  R_Date("VERA-4750",3775,35);
  R_Date("VERA-4757",3845,35);
  R_Date("Erl-4721",4007,62);
```

```
R_Date("Erl-4719",3990,54);
R_Date("Poz-44322",3940,40);
R_Date("VERA-5530",3935,40);
R_Date("R-combine 29",3839,30);
R_Date("R-combine 30",3853,30);
R_Date("VERA-5533",3900,40);
R_Date("VERA-5209",3885,35);
R_Date("R-combine 39",3853,30);
R_Date("VERA-5206",3860,35);
R_Date("Erl-4720",3859,57);
R_Date("VERA-5532",3855,40);
R_Date("VERA-5207",3850,35);
R_Date("VERA-5529",3845,40);
R_Date("VERA-5528",3835,40);
R_Date("VERA-5208",3820,40);
R_Date("VERA-5205",3800,35);
R_Date("VERA-5531",3780,40);
R_Date("KIA-34838",3980,35);
R_Date("KIA-34665",3950,50);
R_Date("KIA-34666",3935,25);
R_Date("KIA-34669",3900,25);
R_Date("KIA-34840",3875,30);
R_Date("KIA-34842",3865,35);
R_Date("R-combine 79",3830,22);
R_Date("KIA-34686",3835,30);
R_Date("KIA-34845",3835,35);
R_Date("KIA-34836",3830,30);
R_Date("KIA-34835",3825,30);
R_Date("R-combine 84",3809,20);
R_Date("KIA-34833",3805,30);
R_Date("KIA-34670",3790,30);
R_Date("KIA-34841",3790,35);
R_Date("KIA-34844",3790,25);
R_Date("KIA-34837",3785,35);
R_Date("KIA-34834",3780,30);
R_Date("KIA-34843",3775,30);
R_Date("KIA-34839",3760,30);
R_Date("Poz-86947",3755,35);
R_Date("Poz-75954",3780,35);
```

```
  R_Date("Poz-66259",3860,40);
  R_Date("Poz-75936",3830,35);
  R_Date("Poz-66185",3825,35);
  R_Date("Poz-75951",3790,35);
  R_Date("Poz-66184",3785,35);
  R_Date("Gd-5117",3910,50);
  R_Date("R-combine 474",3644,30);
  R_Date("Poz-34734",3830,35);
  R_Date("R-combine 476",3857,19);
  R_Date("PSUAMS-2321",3825,25);
  R_Date("R-combine 478",3787,19);
  R_Date("Ki-6153",3790,40);
  R_Date("Ki-6239",3820,50);
  R_Date("Poz-86950",3545,35);
  R_Date("Poz-65207",3770,30);
  R_Date("Ki-7922",3870,60);
  R_Date("Ki-7923",3850,50);
  R_Date("Ki-7924",3940,50);
  R_Date("Ki-7925",3885,50);
  R_Date("Ki-7927",3850,50);
  R_Date("Ki-7928",3870,60);
  R_Date("Ki-6335",3730,40);
 };
};
```

OLE Code for Modelling ‘Start’ and ‘End’ Dates

Author:

Igor Djakovic*

*Faculty of Archaeology, Leiden University, Einsteinweg 2, 2333CC Leiden, the Netherlands

```
#### Set up
library(dplyr)
alpha <- 0.05
iteration <- 10000

######### Creating tables for results
OLE.results <- matrix(0,1,5)
for.plots <- matrix(0,iteration,1)
OLE.results[,1] <- c("Extinction")

##### Import data from CSV
datalist <- read.csv("PATH TO CSV FILE", header=FALSE, sep=';')

######## Creating OLE calculation function (taken and adapted from sExtinct package)
OLE.test <-
  function(dd, alpha){
    # records are sorted in a reverse order, as required by OLE method
    sights <- rev(sort(dd))
    # calculation of k, v, e, lambda and other values
    k <- length(sights)
    v <- (1/(k-1)) * sum(log((sights[1] - sights[k])/(sights[1] - sights[2:(k-1)])))
    e <- matrix(rep(1,k), ncol=1)
    SU<-(-log(alpha)/length(sights))^-v
    myfun <- function(i,j,v){(gamma(2*v+i)*gamma(v+j))/(gamma(v+i)*gamma(j))}
    lambda <- outer(1:k, 1:k, myfun, v=v)
```

```
    lambda <- ifelse(lower.tri(lambda), lambda, t(lambda))
    a <- as.vector(solve(t(e)%*%solve(lambda)%*%e)) * solve(lambda)%*%e
    # calculation of CI ("upperCI") and extinction time ("extest")
    upperCI<-max(sights) + ((max(sights)-min(sights))/(SU-1))
    extest<-sum(t(a)%*%sights)
    # return of results produced by the function
    res<-data.frame(Estimate=extest, upperCI=upperCI)
    return(res)
  }

#### EXTINCTION ESTIMATE ####################
datalist <- datalist %>%
  mutate_if(is.numeric, funs(. * -1))

####### applying OLE to each of the eight datasets
for (i in 1:1) {
  # data file is split into separate datasets based on the first column
  dataset <- datalist[which(datalist[,1]==OLE.results[i,1]),2:4]
  # record timing is adapted for OLE by expressing them as time since the earliest record in the dataset
  mean.record <- dataset[,1] - min(dataset[,1]) + 1
  # OLE function is applied to the data
  OLE <- OLE.test(mean.record,alpha)
  # results are included in the matrix with results
  OLE.results[i,2] <- min(dataset[,1]) + OLE[[1]]
  OLE.results[i,4] <- min(dataset[,1]) + OLE[[2]]

    ###### applying OLE to random samples taken from ranges of age estimates
    # vectors are established where results from random sampling will be stored
    lowCI <- dataset[,1] - dataset[,3]
    highCI <- dataset[,2] - dataset[,1]
    TE.list <- numeric(0)
    CI.list <- numeric(0)
```

```
  # loop counter is established, which will count number of random samples generated
  loop <- 1
  # start of the random sampling loop
  while(loop <= iteration) {
    uniqueSampleFound <- FALSE

    while(!uniqueSampleFound) {
      dd <- numeric(length = length(dataset[,1]))
      for(ii in 1:length(dataset[,1])) {
        repeat {
          potentialSample <- round(runif(1, min = dataset[ii,2], max = dataset[ii,3]))
          if(!(potentialSample %in% dd)) {
            dd[ii] <- potentialSample
            break
          }
        }
      }
      if(length(unique(dd)) == length(dd)) {
        uniqueSampleFound <- TRUE
      }
    }

    # record timing is adapted for OLE by expressing them as time since the earliest record in the
dataset
    sample.record <- dd - min(dd) + 1
    # OLE function is applied to the data
    OLE <- OLE.test(sample.record,alpha)
    # results are included in vectors with results
    TE.list <- c(TE.list, (min(dd) + OLE[[1]]))
    CI.list <- c(CI.list, (min(dd) + OLE[[2]]))
    # loop counter increases by 1
    loop <- loop + 1
  }
```

```
}

# results are included in the matrix with results
OLE.results[i,2] <- min(dataset[,1]) + OLE[[1]]
OLE.results[i,4] <- min(dataset[,1]) + OLE[[2]]

###### applying OLE to random samples taken from ranges of age estimates
# vectors are established where results from random sampling will be stored
lowCI <- dataset[,1] - dataset[,2]
highCI <- dataset[,3] - dataset[,1]
TE.list <- numeric(0)
CI.list <- numeric(0)
# loop counter is established, which will count number of random samples generated
loop <- 1
# start of the random sampling loop
while(loop <= iteration) {
 uniqueSampleFound <- FALSE

 while(!uniqueSampleFound) {
  dd <- numeric(length = length(dataset[,1]))
  for(ii in 1:length(dataset[,1])) {
   repeat {
    potentialSample <- round(runif(1, min = dataset[ii,3], max = dataset[ii,2]))
    if(!(potentialSample %in% dd)) {
     dd[ii] <- potentialSample
     break
    }
   }
  }
  if(length(unique(dd)) == length(dd)) {
   uniqueSampleFound <- TRUE
  }
 }
```

```
    # record timing is adapted for OLE by expressing them as time since the earliest record in the dataset
    sample.record <- dd - min(dd) + 1
    # OLE function is applied to the data
    OLE <- OLE.test(sample.record,alpha)
    # results are included in vectors with results
    TE.list <- c(TE.list, (min(dd) + OLE[[1]]))
    CI.list <- c(CI.list, (min(dd) + OLE[[2]]))
    # loop counter increases by 1
    loop <- loop + 1
  }
  }
  #mean values are taken from vectors with results are included in the matrix with results
  OLE.results[i,3] <- mean(TE.list)
  OLE.results[i,5] <- mean(CI.list)
  for.plots[,i] <- TE.list
  print(i)
####### show OLE results and generate csv files with results
print(OLE.results)
write.csv(for.plots, file="PATH TO LOCATION FOR EXPORT OF EMERGENCE RESAMPLING RESULTS")
```

```
  }
  #mean values are taken from vectors with results are included in the matrix with results
  OLE.results[i,3] <- mean(TE.list)
  OLE.results[i,5] <- mean(CI.list)
  for.plots[,i] <- TE.list
  print(i)
}

####### convert for.plots to positive numbers
for.plots <- abs(for.plots)

####### show OLE results and generate csv files with results
print(OLE.results)
write.csv(for.plots, file="PATH TO LOCATION FOR EXPORT OF EXTINCTION RESAMPLING
RESULTS")

#### EMERGENCE ESTIMATE ############################################

OLE.results <- matrix(0,1,5)
for.plots <- matrix(0,iteration,1)
OLE.results[,1] <- c("Emergence")

datalist <- datalist %>%
  mutate_if(is.numeric, funs(. * -1))

####### applying OLE to each of the eight datasets
for (i in 1:1) {
  # data file is split into separate datasets based on the first column
  dataset <- datalist[which(datalist[,1]==OLE.results[i,1]),2:4]
  # record timing is adapted for OLE by expressing them as time since the earliest record in the dataset
  mean.record <- dataset[,1] - min(dataset[,1]) + 1
  # OLE function is applied to the data
  OLE <- OLE.test(mean.record,alpha)
```

Fig. S1.

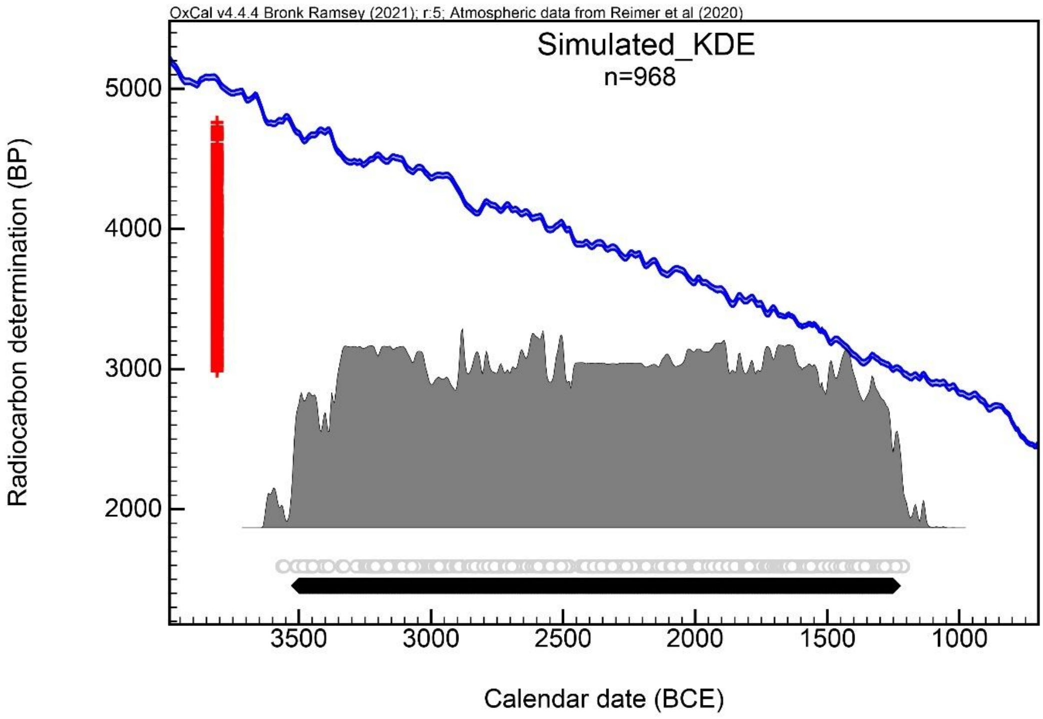

Supplementary Figure 1: Simulated KDE_Model with 968 randomly generated radiocarbon dates using the OxCal Simulate function. (see Supplementary Text).

Data S1. (separate file)

S1 : Radiocarbon dates directly associated with Corded Ware burials.

S2 : Radiocarbon dates directly associated with Bell Beaker burials.

S3 : Excluded Radiocarbon dates with no or poor association with Corded Ware burials.

S4 : Excluded Radiocarbon dates with no or poor association with Corded Ware burials.

S5 : References for all radiocarbon dates in S1 to S4.

S6 : Metadata comparison steppe ancestry vs CW and BB burial rituals

S7 : Comparison of points with arrival of steppe ancestry according to Racimo *et al.* 2020 vs CW and BB burial rituals

S8 : Comparison of points with arrival of steppe ancestry according to Allentoft *et al.* 2024 vs CW and BB burial rituals

REFERENCES AND NOTES

1. I. Lazaridis, N. Patterson, D. Anthony, L. Vyazov, R. Fournier, H. Ringbauer, I. Olalde, A. A. Khokhlov, E. P. Kitov, N. I. Shishlina, S. C. Ailincăi, D. S. Agapov, S. A. Agapov, E. Batieva, B. Bauyrzhan, Z. Bereczki, A. Buzhilova, P. Changmai, A. A. Chizhevsky, I. Ciobanu, M. Constantinescu, M. Csányi, J. Dani, P. K. Dashkovskiy, S. Évinger, A. Faifert, P. Flegontov, A. Frînculeasa, M. N. Frînculeasa, T. Hajdu, T. Higham, P. Jarosz, P. Jelínek, V. I. Khartanovich, E. N. Kirginekov, V. Kiss, A. Kitova, A. V. Kiyashko, J. Koledin, A. Korolev, P. Kosintsev, G. Kulcsár, P. Kuznetsov, R. Magomedov, A. M. Mamedov, E. Melis, V. Moiseyev, E. Molnár, J. Monge, O. Negrea, N. A. Nikolaeva, M. Novak, M. Ochir-Goryaeva, G. Pálfi, S. Popovici, M. P. Rykun, T. M. Savenkova, V. P. Semibratov, N. N. Seregin, A. Šefčáková, R. S. Mussayeva, I. Shingiray, V. N. Shirokov, A. Simalcsik, K. Sirak, K. N. Solodovnikov, J. Tárnoki, A. A. Tishkin, V. Trifonov, S. Vasilyev, A. Akbari, E. S. Brielle, K. Callan, F. Candilio, O. Cheronet, E. Curtis, O. Flegontova, L. Iliev, A. Kearns, D. Keating, A. M. Lawson, M. Mah, A. Micco, M. Michel, J. Oppenheimer, L. Qiu, J. N. Workman, F. Zalzala, A. Szécsényi-Nagy, P. F. Palamara, S. Mallick, N. Rohland, R. Pinhasi, D. Reich, The genetic origin of the Indo-Europeans. *Nature* **639**, 132–142 (2025).

2. W. Haak, I. Lazaridis, N. Patterson, N. Rohland, S. Mallick, B. Llamas, G. Brandt, S. Nordenfelt, E. Harney, K. Stewardson, Q. Fu, A. Mittnik, E. Bánffy, C. Economou, M. Francken, S. Friederich, R. G. Pena, F. Hallgren, V. Khartanovich, A. Khokhlov, M. Kunst, P. Kuznetsov, H. Meller, O. Mochalov, C. Roth, A. Szécsényi-Nagy, J. Wahl, M. Meyer, J. Krause, D. Brown, D. Anthony, A. Cooper, K. W. Alt, D. Reich, Massive migration from the steppe was a source for Indo-European languages in Europe. *Nature* **522**, 207–211 (2015).

3. M. E. Allentoft, M. Sikora, K.-G. Sjögren, S. Rasmussen, M. Rasmussen, J. Stenderup, P. B. Damgaard, H. Schroeder, T. Ahlström, L. Vinner, A.-S. Malaspinas, A. Margaryan, T. Higham, D. Chivall, N. Lynnerup, L. Harvig, J. Baron, P. Della Casa, P. Dąbrowski, P. R. Duffy, A. V. Ebel, A. Epimakhov, K. Frei, M. Furmanek, T. Gralak, A. Gromov, S. Gronkiewicz, G. Grupe, T. Hajdu, R. Jarysz, V. Khartanovich, A. Khokhlov, V. Kiss, J. Kolář, A. Kriiska, I. Lasak, C. Longhi, G. McGlynn, A. Merkevicius, I. Merkyte, M. Metspalu, R. Mkrtchyan, V. Moiseyev, L. Paja, G. Pálfi, D. Pokutta, Ł. Pospieszny, T. D. Price, L. Saag, M. Sablin, N. Shishlina, V. Smrčka, V. I. Soenov, V. Szeverényi, G. Tóth, S. V. Trifanova, L. Varul, M. Vicze, L. Yepiskoposyan, V. Zhitenev, L. Orlando, T. Sicheritz-Pontén, S. Brunak, R. Nielsen, K.

Kristiansen, E. Willerslev, Population genomics of Bronze Age Eurasia. *Nature* **522**, 167–172 (2015).

4. I. Olalde, S. Brace, M. E. Allentoft, I. Armit, K. Kristiansen, T. Booth, N. Rohland, S. Mallick, A. Szécsényi-Nagy, A. Mittnik, E. Altena, M. Lipson, I. Lazaridis, T. K. Harper, N. Patterson, N. Broomandkhoshbacht, Y. Diekmann, Z. Faltyskova, D. Fernandes, M. Ferry, E. Harney, P. de Knijff, M. Michel, J. Oppenheimer, K. Stewardson, A. Barclay, K. W. Alt, C. Liesau, P. Ríos, C. Blasco, J. V. Miguel, R. M. García, A. A. Fernández, E. Bánffy, M. Bernabò-Brea, D. Billoin, C. Bonsall, L. Bonsall, T. Allen, L. Büster, S. Carver, L. C. Navarro, O. E. Craig, G. T. Cook, B. Cunliffe, A. Denaire, K. E. Dinwiddy, N. Dodwell, M. Ernée, C. Evans, M. Kuchařík, J. F. Farré, C. Fowler, M. Gazenbeek, R. G. Pena, M. Haber-Uriarte, E. Haduch, G. Hey, N. Jowett, T. Knowles, K. Massy, S. Pfrengle, P. Lefranc, O. Lemercier, A. Lefebvre, C. H. Martínez, V. G. Olmo, A. B. Ramírez, J. L. Maurandi, T. Majó, J. I. McKinley, K. McSweeney, B. G. Mende, A. Mod, G. Kulcsár, V. Kiss, A. Czene, R. Patay, A. Endrődi, K. Köhler, T. Hajdu, T. Szeniczey, J. Dani, Z. Bernert, M. Hoole, O. Cheronet, D. Keating, P. Velemínský, M. Dobeš, F. Candilio, F. Brown, R. F. Fernández, A.-M. Herrero-Corral, S. Tusa, E. Carnieri, L. Lentini, A. Valenti, A. Zanini, C. Waddington, G. Delibes, E. Guerra-Doce, B. Neil, M. Brittain, M. Luke, R. Mortimer, J. Desideri, M. Besse, G. Brücken, M. Furmanek, A. Hałuszko, M. Mackiewicz, A. Rapiński, S. Leach, I. Soriano, K. T. Lillios, J. L. Cardoso, M. P. Pearson, P. Włodarczak, T. D. Price, P. Prieto, P.-J. Rey, R. Risch, M. A. R. Guerra, A. Schmitt, J. Serralongue, A. M. Silva, V. Smrčka, L. Vergnaud, J. Zilhão, D. Caramelli, T. Higham, M. G. Thomas, D. J. Kennett, H. Fokkens, V. Heyd, A. Sheridan, K.-G. Sjögren, P. W. Stockhammer, J. Krause, R. Pinhasi, W. Haak, I. Barnes, C. Lalueza-Fox, D. Reich, The Beaker phenomenon and the genomic transformation of northwest Europe. *Nature* **555**, 190–196 (2018).

5. M. E. Allentoft, M. Sikora, A. Refoyo-Martínez, E. K. Irving-Pease, A. Fischer, W. Barrie, A. Ingason, J. Stenderup, K.-G. Sjögren, A. Pearson, B. S. da Mota, B. S. Paulsson, A. Halgren, R. Macleod, M. L. S. Jørkov, F. Demeter, L. Sørensen, P. O. Nielsen, R. A. Henriksen, T. Vimala, H. McColl, A. Margaryan, M. Ilardo, A. Vaughn, M. F. Mortensen, A. B. Nielsen, M. U. Hede, N. N. Johannsen, P. Rasmussen, L. Vinner, G. Renaud, A. Stern, T. Z. T. Jensen, G. Scorrano, H. Schroeder, P. Lysdahl, A. D. Ramsøe, A. Skorobogatov, A. J. Schork, A. Rosengren, A. Ruter, A. Outram, A. A. Timoshenko, A. Buzhilova, A. Coppa, A. Zubova, A.

M. Silva, A. J. Hansen, A. Gromov, A. Logvin, A. B. Gotfredsen, B. H. Nielsen, B. González-Rabanal, C. Lalueza-Fox, C. J. McKenzie, C. Gaunitz, C. Blasco, C. Liesau, C. Martinez-Labarga, D. V. Pozdnyakov, D. Cuenca-Solana, D. O. Lordkipanidze, D. En'shin, D. C. Salazar-García, T. D. Price, D. Borić, E. Kostyleva, E. V. Veselovskaya, E. R. Usmanova, E. Cappellini, E. B. Petersen, E. Kannegaard, F. Radina, F. E. Yediay, H. Duday, I. Gutiérrez-Zugasti, I. Merts, I. Potekhina, I. Shevnina, I. Altinkaya, J. Guilaine, J. Hansen, J. E. A. Tortosa, J. Zilhão, J. Vega, K. B. Pedersen, K. Tunia, L. Zhao, L. N. Mylnikova, L. Larsson, L. Metz, L. Yepiskoposyan, L. Pedersen, L. Sarti, L. Orlando, L. Slimak, L. Klassen, M. Blank, M. González-Morales, M. Silvestrini, M. Vretemark, M. S. Nesterova, M. Rykun, M. F. Rolfo, M. Szmyt, M. Przybyła, M. Calattini, M. Sablin, M. Dobisíková, M. Meldgaard, M. Johansen, N. Berezina, N. Card, N. A. Saveliev, O. Poshekhonova, O. Rickards, O. V. Lozovskaya, O. Gábor, O. C. Uldum, P. Aurino, P. Kosintsev, P. Courtaud, P. Ríos, P. Mortensen, P. Lotz, P. Persson, P. Bangsgaard, P. de Barros Damgaard, P. V. Petersen, P. P. Martinez, P. Włodarczak, R. V. Smolyaninov, R. Maring, R. Menduiña, R. Badalyan, R. Iversen, R. Turin, S. Vasilyev, S. Wåhlin, S. Borutskaya, S. Skochina, S. A. Sørensen, S. H. Andersen, T. Jørgensen, Y. B. Serikov, V. I. Molodin, V. Smrcka, V. Merts, V. Appadurai, V. Moiseyev, Y. Magnusson, K. H. Kjær, N. Lynnerup, D. J. Lawson, P. H. Sudmant, S. Rasmussen, T. S. Korneliussen, R. Durbin, R. Nielsen, O. Delaneau, T. Werge, F. Racimo, K. Kristiansen, E. Willerslev, Population genomics of post-glacial western Eurasia. *Nature* **625**, 301–311 (2024).

6. D. Hofmann, What have genetics ever done for us? The implications of aDNA data for interpreting identity in early neolithic central Europe. *Eur. J. Archaeol.* **18**, 454–476 (2015).

7. C. J. Frieman, D. Hofmann, Present pasts in the archaeology of genetics, identity, and migration in Europe: A critical essay. *World Archaeol.* **51**, 528–545 (2019).

8. S. Alpaslan-Roodenberg, D. Anthony, H. Babiker, E. Bánffy, T. Booth, P. Capone, A. Deshpande-Mukherjee, S. Eisenmann, L. Fehren-Schmitz, M. Frachetti, R. Fujita, C. J. Frieman, Q. Fu, V. Gibbon, W. Haak, M. Hajdinjak, K. P. Hofmann, B. Holguin, T. Inomata, H. Kanzawa-Kiriyama, W. Keegan, J. Kelso, J. Krause, G. Kumaresan, C. Kusimba, S. Kusimba, C. Lalueza-Fox, B. Llamas, S. MacEachern, S. Mallick, H. Matsumura, A. Y. Morales-Arce, G. M. Matuzeviciute, V. Mushrif-Tripathy, N. Nakatsuka, R. Nores, C. Ogola, M. Okumura, N. Patterson, R. Pinhasi, S. P. R. Prasad, M. E. Prendergast, J. L. Punzo, D.

Reich, R. Sawafuji, E. Sawchuk, S. Schiffels, J. Sedig, S. Shnaider, K. Sirak, P. Skoglund, V. Slon, M. Snow, M. Soressi, M. Spriggs, P. W. Stockhammer, A. Szécsényi-Nagy, K. Thangaraj, V. Tiesler, R. Tobler, C.-C. Wang, C. Warinner, S. Yasawardene, M. Zahir, Ethics of DNA research on human remains: Five globally applicable guidelines. *Nature* **599**, 41–46 (2021).

9. S. Eisenmann, E. Bánffy, P. van Dommelen, K. P. Hofmann, J. Maran, I. Lazaridis, A. Mittnik, M. McCormick, J. Krause, D. Reich, P. W. Stockhammer, Reconciling material cultures in archaeology with genetic data: The nomenclature of clusters emerging from archaeogenomic analysis. *Sci. Rep.* **8**, 13003 (2018).

10. T. J. Booth, A stranger in a strange land: A perspective on archaeological responses to the palaeogenetic revolution from an archaeologist working amongst palaeogeneticists. *World Archaeol.* **51**, 586–601 (2019).

11. A. Mittnik, C. C. Wang, S. Pfrengle, M. Daubaras, G. Zariņa, F. Hallgren, R. Allmäe, V. Khartanovich, V. Moiseyev, M. Tõrv, A. Furtwängler, A. Andrades Valtueña, M. Feldman, C. Economou, M. Oinonen, A. Vasks, E. Balanovska, D. Reich, R. Jankauskas, W. Haak, S. Schiffels, J. Krause, The genetic prehistory of the Baltic Sea region. *Nat. Commun.* **9**, 442 (2018).

12. H. Malmström, T. Günther, E. M. Svensson, A. Juras, M. Fraser, A. R. Munters, Ł. Pospieszny, M. Tõrv, J. Lindström, A. Götherström, J. Storå, M. Jakobsson, The genomic ancestry of the Scandinavian Battle Axe Culture people and their relation to the broader Corded Ware horizon. *Proc. R. Soc. B Biol. Sci.* **286**, 20191528 (2019).

13. L. Papac, M. Ernée, M. Dobeš, M. Langová, A. B. Rohrlach, F. Aron, G. U. Neumann, M. A. Spyrou, N. Rohland, P. Velemínský, M. Kuna, H. Brzobohatá, B. Culleton, D. Daněček, A. Danielisová, M. Dobisíková, J. Hložek, D. J. Kennett, J. Klementová, M. Kostka, P. Krištuf, M. Kuchařík, J. K. Hlavová, P. Limburský, D. Malyková, L. Mattiello, M. Pecinovská, K. Petriščáková, E. Průchová, P. Stránská, L. Smejtek, J. Špaček, R. Šumberová, O. Švejcar, M. Trefný, M. Vávra, J. Kolář, V. Heyd, J. Krause, R. Pinhasi, D. Reich, S. Schiffels, W. Haak, Dynamic changes in genomic and social structures in third millennium BCE central Europe. *Sci. Adv.* **7**, eabi6941 (2021).

14. M. Furholt, Mobility and social change: Understanding the European neolithic period after the archaeogenetic revolution. *J. Archaeol. Res.* **29**, 481–535 (2021).

15. M. Vander Linden, *The Bell Beaker Phenomenon in Europe* (Cambridge Univ. Press, 2024); www.cambridge.org/core/product/identifier/9781009496872/type/element.

16. Q. Bourgeois, E. Kroon, "Emergent properties of the Corded Ware culture: An information approach," in *The Indo-European Puzzle Revisited* (Cambridge Univ. Press, 2023), pp. 81–92.

17. Q. Bourgeois, E. Kroon, The impact of male burials on the construction of Corded Ware identity: Reconstructing networks of information in the 3rd millennium BC. *PLOS ONE* **12**, e0185971 (2017).

18. J. Robb, O. J. T. Harris, Becoming gendered in European prehistory: Was neolithic gender fundamentally different? *Am. Antiq.* **83**, 128–147 (2018).

19. K. Kristiansen, M. E. Allentoft, K. M. Frei, R. Iversen, N. N. Johannsen, G. Kroonen, Ł. Pospieszny, T. D. Price, S. Rasmussen, K.-G. Sjögren, M. Sikora, E. Willerslev, Re-theorising mobility and the formation of culture and language among the Corded Ware culture in Europe. *Antiquity* **91**, 334–347 (2017).

20. J. Müller, "Demographic traces of technological innovation, social change and mobility: From 1 to 8 million Europeans (6000–2000 BCE)," in *Environment and Subsistence - Forty Years after Janusz Kruk's Settlement Studies*, S. Kadrow, P. Wlodarczak, Eds. (Dr. Rudolf Habelt GmbH, 2013), pp. 1–14.

21. K. Sjögren, T. D. Price, K. Kristiansen, Diet and mobility in the Corded Ware of Central Europe. *PLOS ONE* **11**, e0155083 (2016).

22. V. Heyd, "Yamnaya, Corded Wares, and Bell Beakers on the move," in *Yamnaya Interactions: Proceedings of the International Workshop Held in Helsinki, 25-26 April 2019*, V. Heyd, G. Kulcsár, B. Preda-Bălănică, Eds. (Archaeolingua, 2021), pp. 383–414.

23. T. J. Booth, J. Brück, S. Brace, I. Barnes, Tales from the supplementary information: Ancestry change in chalcolithic–Early bronze age britain was gradual with varied kinship organization. *Camb. Archaeol. J.* **31**, 379–400 (2021).

24. F. Racimo, J. Woodbridge, R. M. Fyfe, M. Sikora, K. G. Sjögren, K. Kristiansen, M. Vander Linden, The spatiotemporal spread of human migrations during the European Holocene. *Proc. Natl. Acad. Sci. U.S.A.* **117**, 8989–9000 (2020).

25. A. Scott, S. Reinhold, T. Hermes, A. A. Kalmykov, A. Belinskiy, A. Buzhilova, N. Berezina, A. R. Kantorovich, V. E. Maslov, F. Guliyev, B. Lyonnet, P. Gasimov, B. Jalilov, J. Eminli, E. Iskandarov, E. Hammer, S. E. Nugent, R. Hagan, K. Majander, P. Onkamo, K. Nordqvist, N. Shishlina, E. Kaverzneva, A. I. Korolev, A. A. Khokhlov, R. V. Smolyaninov, S. V. Sharapova, R. Krause, M. Karapetian, E. Stolarczyk, J. Krause, S. Hansen, W. Haak, C. Warinner, Emergence and intensification of dairying in the Caucasus and Eurasian steppes. *Nat. Ecol. Evol.* **6**, 813–822 (2022).

26. J. Müller, *Soziochronologische Studien Zum Jung- Und Spätneolithikum Im Mittelelbe-Saale-Gebiet (4100-2700 v.Chr.): Eine Sozialhistorische Interpretation Prähistorischer Quellen*, vol. 21 of *Vorgeschichtliche Forschungen Band* (Leidorf, 2001). [Sociochronological Studies on the Early and Late Neolithic in the Middle Elbe-Saale Region (4100-2700 BC): A Sociohistorical Interpretation of Prehistoric Sources].

27. J. N. Lanting, J. Van der Plicht, De 14C-chronologie van de Nederlandse pre- en protohistorie III: Neolithicum. *Palaeohistoria* , 1–110 (2000). [The 14C Chronology of Dutch Pre- and Protohistory III: Neolithicum].

28. R.-D. Fabian, "Rezension zu: Martin Furholt, Die Absolutchronologische Datierung Der Schnurkeramik in Mitteleuropa Und Südskandinavien" (Dr. Rudolf Habelt GmbH, 2003), vol. 101. https://doi.org/10.11588/ger.1999.60284. [Review of: Martin Furholt, The Absolute Chronological Dating of the Corded Ware Culture in Central Europe and Southern Scandinavia].

29. C. Bronk Ramsey, Methods for summarizing radiocarbon datasets. *Radiocarbon* **59**, 1809–1833 (2017).

30. A. Key, D. Roberts, I. Jarić, Reconstructing the full temporal range of archaeological phenomena from sparse data. *J. Archaeol. Sci.* **135**, 105479 (2021).

31. G. Brandt, W. Haak, C. J. Adler, C. Roth, A. Szécsényi-Nagy, S. Karimnia, S. Möller-Rieker, H. Meller, R. Ganslmeier, S. Friederich, V. Dresely, N. Nicklisch, J. K. Pickrell, F. Sirocko, D. Reich, A. Cooper, K. W. Alt, Ancient DNA reveals key stages in the formation of Central European mitochondrial genetic diversity. *Science* **342**, 257–261 (2013).

32. V. Heyd, Kossinna's smile. *Antiquity* **91**, 348–359 (2017).

33. M. Furholt, Upending a 'totality': Re-evaluating Corded Ware variability in late neolithic Europe. *Proc. Prehist. Soc.* **80**, 1–20 (2014).

34. D. L. Roberts, A. R. Solow, When did the dodo become extinct. *Nature* **426**, 245 (2003).

35. I. Djakovic, A. Key, M. Soressi, Optimal linear estimation models predict 1400–2900 years of overlap between *Homo sapiens* and Neandertals prior to their disappearance from France and northern Spain. *Sci. Rep.* **12**, 15000 (2022).

36. A. Juras, M. Chyleński, E. Ehler, H. Malmström, D. Żurkiewicz, P. Włodarczak, S. Wilk, J. Peška, P. Fojtík, M. Králík, J. Libera, J. Bagińska, K. Tunia, V. I. Klochko, M. Dabert, M. Jakobsson, A. Kośko, Mitochondrial genomes reveal an east to west cline of steppe ancestry in Corded Ware populations. *Sci. Rep.* **8**, 11603 (2018).

37. R. Harrison, V. Heyd, The Transformation of Europe in the Third Millennium BC: The example of 'Le Petit-Chasseur I + III' (Sion, Valais, Switzerland). *Praehistorische Zeitschrift* **82**, 129–214 (2007).

38. R. J. Harrison, *The Beaker Folk. Copper Age Archaeology in Western Europe* (Thames and Hudson, 1980).

39. A. C. Valera, R. Mataloto, A. C. Basílio, "The South Portugal perspective. Beaker sites or sites with Beakers?" in *Bell Beaker Settlement of Europe. The Bell Beaker Phenomenon from a Domestic Perspective*, A. M. Gibson, Ed. (Oxbow Books, 2019), pp. 1–23.

40. I. Armit, D. Reich, The return of the Beaker folk? Rethinking migration and population change in British prehistory. *Antiquity* **95**, 1464–1477 (2021).

41. E. Oras, M. Tõrv, K. Johanson, E. Rannamäe, A. Poska, L. Lõugas, A. Lucquin, J. Lundy, S. Brown, S. Chen, L. Varul, V. Haferberga, D. Legzdiņa, G. Zariņa, L. Cramp, V. Heyd, M. Reay, Ł. Pospieszny, H. K. Robson, K. Nordqvist, C. Heron, O. E. Craig, A. Kriiska, Parallel worlds and mixed economies: Multi-proxy analysis reveals complex subsistence systems at the dawn of early farming in the northeast Baltic. *R. Soc. Open Sci.* **10**, 230880 (2023).

42. E. Kroon, *Serial Learners. Interactions between Funnel Beaker West and Corded Ware Communities in the Netherlands during the Third Millennium BCE from the Perspective of Ceramic Technology* (Sidestone Press Dissertations, 2024).

43. A. Bloxam, M. P. Pearson, Funerary diversity and cultural continuity: The British Beaker Phenomenon beyond the stereotype. *Proc. Prehist. Soc.* **88**, 261–284 (2022).

44. R. Iversen, *Transformation of Neolithic Societies: An East Danish Perspective on the 3rd Millennium BC* (Det Humanistiske Fakultet, Københavns Universitet, 2014).

45. D. Centola, A. Baronchelli, The spontaneous emergence of conventions: An experimental study of cultural evolution. *Proc. Natl. Acad. Sci. U.S.A.* **112**, 201418838 (2015).

46. D. Centola, J. Becker, D. Brackbill, A. Baronchelli, Experimental evidence for tipping points in social convention. *Science* **360**, 1116–1119 (2018).

47. V. Heyd, "When the West meets the East: The Eastern Periphery of the Bell Beaker Phenomenon and its Relation with the Aegean Early Bronze Age," in *Between the Aegean and Baltic Seas*, I. Galanaki, Ed. (Universitè de Liège, Histoire de l'Art et Archéologie de la Grèce Antique, 2007), pp. 91–107.

48. J. Czebrszuk, J. Müller, *Die Absolute Chronologie in Mitteleuropa 3.000-2.000 v.Chr. / The Absolute Chronology of Central Europe 3.000-2.000 B.C.* (Leidorf, 2001).

49. F. Bertemes, V. Heyd, “The transition Copper Age/Early Bronze Age at the north-western edge of the Carpatian Basin - culture-historical and palaeometallurgical considerations,” in *Die Anfange Der Metallurgie in Der Alten Welt. The Beginnings of Metallurgy in the Old World*, M. Bartelheim, R. Krause, E. Pernicka, Eds. (Verlag Marie Leidorf, 2002), pp. 185–228.

50. H. Meller, Armies in the early bronze age? An alternative interpretation of Únětice Culture axe hoards. *Antiquity* **91**, 1529–1545 (2017).

51. H. W. Nørgaard, E. Pernicka, H. Vandkilde, Shifting networks and mixing metals: Changing metal trade routes to Scandinavia correlate with Neolithic and Bronze Age transformations. *PLOS ONE* **16**, e0252376 (2021).

52. H. Fokkens, B. Steffens, S. van As, *Farmers, Fishers, Fowlers, Hunters: Knowledge Generated by Development-Led Archaeology in the Late Neolithic, the Early Bronze Age and the Start of the Middle Bronze Age (2850-1500 Cal BC) in the Netherlands (Nederlandse Archeologische Rapporten 53)* (Rijksdienst voor het Cultureel Erfgoed, 2006).

53. M. Parker Pearson, A. Sheridan, M. Jay, A. Chamberlain, J. Evans, *The Beaker People* (Oxbow Books, 2019).

54. K. Wentink, *Stereotype. The Role of Grave Sets in Corded Ware and Bell Beaker Funerary Practices* (Sidestone Press, 2020).

55. H. Meller, Princes, armies, sanctuaries: The emergence of complex authority in the central german únětice culture. *Acta Archaeologica* **90**, 39–79 (2019).

56. P. Librado, N. Khan, A. Fages, M. A. Kusliy, T. Suchan, L. Tonasso-Calvière, S. Schiavinato, D. Alioglu, A. Fromentier, A. Perdereau, J. M. Aury, C. Gaunitz, L. Chauvey, A. Seguin-Orlando, C. Der Sarkissian, J. Southon, B. Shapiro, A. A. Tishkin, A. A. Kovalev, S. Alquraishi, A. H. Alfarhan, K. A. S. Al-Rasheid, T. Seregély, L. Klassen, R. Iversen, O. Bignon-Lau, P. Bodu, M. Olive, J. C. Castel, M. Boudadi-Maligne, N. Alvarez, M. Germonpré, M. Moskal-del Hoyo, J. Wilczyński, S. Pospuła, A. Lasota-Kuś, K. Tunia, M. Nowak, E. Rannamäe, U. Saarma, G. Boeskorov, L. Lōugas, R. Kyselý, L. Peške, A.

Bălășescu, V. Dumitrașcu, R. Dobrescu, D. Gerber, V. Kiss, A. Szécsényi-Nagy, B. G. Mende, Z. Gallina, K. Somogyi, G. Kulcsár, E. Gál, R. Bendrey, M. E. Allentoft, G. Sirbu, V. Dergachev, H. Shephard, N. Tomadini, S. Grouard, A. Kasparov, A. E. Basilyan, M. A. Anisimov, P. A. Nikolskiy, E. Y. Pavlova, V. Pitulko, G. Brem, B. Wallner, C. Schwall, M. Keller, K. Kitagawa, A. N. Bessudnov, A. Bessudnov, W. Taylor, J. Magail, J. O. Gantulga, J. Bayarsaikhan, D. Erdenebaatar, K. Tabaldiev, E. Mijiddorj, B. Boldgiv, T. Tsagaan, M. Pruvost, S. Olsen, C. A. Makarewicz, S. V. Lamas, S. A. Canadell, A. N. Espinet, M. P. Iborra, J. L. Garrido, E. R. González, S. Celestino, C. Olària, J. L. Arsuaga, N. Kotova, A. Pryor, P. Crabtree, R. Zhumatayev, A. Toleubaev, N. L. Morgunova, T. Kuznetsova, D. Lordkipanize, M. Marzullo, O. Prato, G. B. Gianni, U. Tecchiati, B. Clavel, S. Lepetz, H. Davoudi, M. Mashkour, N. Y. Berezina, P. W. Stockhammer, J. Krause, W. Haak, A. Morales-Muñiz, N. Benecke, M. Hofreiter, A. Ludwig, A. S. Graphodatsky, J. Peters, K. Y. Kiryushin, T. O. Iderkhangai, N. A. Bokovenko, S. K. Vasiliev, N. N. Seregin, K. V. Chugunov, N. A. Plasteeva, G. F. Baryshnikov, E. Petrova, M. Sablin, E. Ananyevskaya, A. Logvin, I. Shevnina, V. Logvin, S. Kalieva, V. Loman, I. Kukushkin, I. Merz, V. Merz, S. Sakenov, V. Varfolomeyev, E. Usmanova, V. Zaibert, B. Arbuckle, A. B. Belinskiy, A. Kalmykov, S. Reinhold, S. Hansen, A. I. Yudin, A. A. Vybornov, A. Epimakhov, N. S. Berezina, N. Roslyakova, P. A. Kosintsev, P. F. Kuznetsov, D. Anthony, G. J. Kroonen, K. Kristiansen, P. Wincker, A. Outram, L. Orlando, The origins and spread of domestic horses from the Western Eurasian steppes. *Nature* **598**, 634–640 (2021).

57. M. Furholt, Social Worlds and Communities of Practice: A polythetic culture model for 3rd millennium BC Europe in the light of current migration debates. *Préhistoires Méditerranéennes*, 10.4000/pm.2383 (2020).

58. C. Bronk Ramsey, OxCal. 4.4.4 (2021); https://c14.arch.ox.ac.uk/oxcal.html.

59. P. J. Reimer, W. E. N. Austin, E. Bard, A. Bayliss, P. G. Blackwell, C. Bronk Ramsey, M. Butzin, H. Cheng, R. L. Edwards, M. Friedrich, P. M. Grootes, T. P. Guilderson, I. Hajdas, T. J. Heaton, A. G. Hogg, K. A. Hughen, B. Kromer, S. W. Manning, R. Muscheler, J. G. Palmer, C. Pearson, J. van der Plicht, R. W. Reimer, D. A. Richards, E. M. Scott, J. R. Southon, C. S. M. Turney, L. Wacker, F. Adolphi, U. Büntgen, M. Capano, S. M. Fahrni, A. Fogtmann-Schulz, R. Friedrich, P. Köhler, S. Kudsk, F. Miyake, J. Olsen, F. Reinig, M.

Sakamoto, A. Sookdeo, S. Talamo, The IntCal20 Northern Hemisphere radiocarbon age calibration curve (0–55 cal kBP). *Radiocarbon* **62**, 725–757 (2020).

60. E. R. Crema, A. Bevan, Inference from large sets of radiocarbon dates: Software and methods. *Radiocarbon* **63**, 23–39 (2021).

61. G. Capuzzo, G. De Mulder, C. Sabaux, S. Dalle, M. Boudin, R. Annaert, M. Hlad, K. Salesse, A. Sengeløv, E. Stamataki, B. Veselka, E. Warmenbol, C. Snoeck, M. Vercauteren, Final neolithic and bronze age funerary practices and population dynamics in belgium, the impact of radiocarbon dating cremated bones. *Radiocarbon* **65**, 51–80 (2023).

62. A. N. Williams, The use of summed radiocarbon probability distributions in archaeology: A review of methods. *J. Archaeol. Sci.* **39**, 578–589 (2012).

63. M. Vidal-Cordasco, G. Terlato, D. Ocio, A. B. Marín-Arroyo, Neanderthal coexistence with *Homo sapiens* in Europe was affected by herbivore carrying capacity. *Sci. Adv.* **9**, eadi4099 (2023).

64. A. R. Solow, Inferring extinction from a sighting record. *Math. Biosci.* **195**, 47–55 (2005).

65. A. J. M. Key, I. Jarić, D. L. Roberts, Modelling the end of the Acheulean at global and continental levels suggests widespread persistence into the Middle Palaeolithic. *Humanit. Soc. Sci. Commun.* **8**, 55 (2021).

66. R. J. Telford, E. Heegaard, H. J. B. Birks, The intercept is a poor estimate of a calibrated radiocarbon age. *Holocene* **14**, 296–298 (2004).

67. C. Bronk Ramsey, Bayesian analysis of radiocarbon dates. *Radiocarbon* **51**, 337–360 (2009).

68. C. F. F. Karney, Algorithms for geodesics. *J. Geod.* **87**, 43–55 (2013).

69. T. Russell, F. Silva, J. Steele, Modelling the spread of farming in the bantu-speaking regions of Africa: An archaeology-based phylogeography. *PLOS ONE* **9**, e87854 (2014).

70. European Environment Agency, Europe coastline shapefile, Environmental Data (2019); www.eea.europa.eu/data-and-maps/data/eea-coastline-for-analysis-1/gis-data/europe-coastline-shapefile.

71. European Environment Agency, SRTM90 - The shuttle radar topography mission DEM100, EAA Datahub (2023); www.eea.europa.eu/en/datahub/datahubitem-view/d0a1dedf-e1df-4e36-a80c-5667002b4057.

72. Esri, World Major Rivers, ArcGIS Online (2023); www.arcgis.com/home/item.html?id=44e8358cf83a4b43bc863646cd695945.

73. Food and Agriculture Organization of the United Nationas, Rivers of Europe, Aquastat (2022); https://data.apps.fao.org/catalog/iso/e0243940-e5d9-487c-8102-45180cf1a99f.

74. M. Hollander, D. A. Wolfe, E. Chicken, *Nonparametric Statistical Methods* (Wiley, 2015).

75. P. Virtanen, R. Gommers, T. E. Oliphant, M. Haberland, T. Reddy, D. Cournapeau, E. Burovski, P. Peterson, W. Weckesser, J. Bright, S. J. van der Walt, M. Brett, J. Wilson, K. J. Millman, N. Mayorov, A. R. J. Nelson, E. Jones, R. Kern, E. Larson, C. J. Carey, İ. Polat, Y. Feng, E. W. Moore, J. VanderPlas, D. Laxalde, J. Perktold, R. Cimrman, I. Henriksen, E. A. Quintero, C. R. Harris, A. M. Archibald, A. H. Ribeiro, F. Pedregosa, P. van Mulbregt, SciPy 1.0 Contributors, SciPy 1.0: Fundamental algorithms for scientific computing in Python. *Nat. Methods* **17**, 261–272 (2020).

76. M. G. Kendall, The treatment of ties in ranking problems. *Biometrika* **33**, 239–251 (1945).

77. M. G. Kendall, A new measure of rank correlation. *Biometrika* **30**, 81 (1938).

78. K. Nordqvist, V. Heyd, The forgotten child of the wider Corded Ware family: Russian fatyanovo culture in context. *Proc. Prehist. Soc.* **86**, 65–93 (2020).

79. D. A. Kraynov, *Drevneishaya Istoriya Volgo-Okskogo Mezhdurech'ya: Fat'yanovskaya Kul'tura, II Tysyacheletie Do n.e [The Oldest History of the Volga-Oka Interfluve: The Fatyanovo Culture, 2nd Millennium Bc]* (Nauka, 1972).

80. D. A. Kraynov, "Fat'yanovskaya kul'tura [Fatyanovo Culture]," in *Epokha Bronzy Lesnoi Polosy SSSR [The Bronze Age of the Forest Belt of the USSR]*, O. N. Bader, D. A. Kraynov, M. F. Kosarev, Eds. (Nauka, 1987), pp. 58–76.

81. O. N. Bader, Kuz'minskii mogil'nik fat'yanovskogo tipa pod Moskvoi [The Kuzminki burial ground of the Fatyanovo type near Moscow]. *Arkheologicheskiy Sbornik Gosudarstvennogo Ermitazha* **5**, 5–30 (1962).

82. N. Krenke, Radiocarbon chronology of the Fatyanovo culture. *Rossiiskaia Arkheologiia* **2**, 110–116 (2019).

83. D. A. Krainov, "On the problem of origin, chronology and periodization of the Fatyanovo-Balanovo cultural community," in *Die Kontinentaleuropäischen Gruppen Der Kultur Mit Schnurkeramik. Schnurkeramik Symposium 1990*, M. Buchvaldek, C. Strahm, Eds. (Universita Karlova, Prague, 1992), pp. 321–327.

84. D. A. Kraynov, Volosovo-Danilovskii mogil'nik Fat'yanovskoi kul'tury [Volosovo-Danilovskiy burial ground of the Fatyanovo Culture]. *Sovetskaya Arkheologiya* **4**, 68–83 (1964).

85. V. M. Raushenbah, "Fat'yanovskoe pogrebenie na neoliticheskoi stoyanke Nikolo-Perevoz [Fatyanovo burial at the Neolithic settlement Nikolo-Perevoz]," in *Arkheologicheskiy Sbornik*, V. P. Levasheva, Ed. (Trudy Gosudarstvennogo Istoricheskogo Muzeya, 1960), vol. 37, pp. 28–37.

86. N. A. Kirianova, Naumovskii fat'yanovskii mogil'nik [Naumovsky Fatyanovo cemetery]. *Kratkie Soobshcheniya* **134**, 73–77 (1973).

87. L. Saag, S. V. Vasilyev, L. Varul, N. V. Kosorukova, D. V. Gerasimov, S. V. Oshibkina, S. J. Griffith, A. Solnik, L. Saag, E. D'Atanasio, E. Metspalu, M. Reidla, S. Rootsi, T. Kivisild, C. L. Scheib, K. Tambets, A. Kriiska, M. Metspalu, Genetic ancestry changes in Stone to Bronze Age transition in the East European plain. *Sci. Adv.* **7**, eabd6535 (2021).

88. A. V. Engovatova, I. E. Alborova, K. K. Mustafin, V. Y. Lunkov, Y. V. Lunkova, A. A. Kanapin, A. A. Samsonova, M. B. Mednikova, Drevnjaja DNK nositelej fat'janovskoj i abashevskoj kul'tur (k voprosu o migracijah naselenija jepohi bronzy v lesnoj polose na Russkoj ravnine)[Ancient DNA of the Bearers of the Fatyanovo and Abashevo Cultures (Concerning Migrations of the Bronze Age peoples in the Forest Belt on the Russian Plain)]. *Stratum plus. No. 2. Archaeol. Cultural Anthropol.* **2**, 207–389 (2023).

89. H. Ringbauer, Y. Huang, A. Akbari, S. Mallick, I. Olalde, N. Patterson, D. Reich, Accurate detection of identity-by-descent segments in human ancient DNA. *Nat. Genet.* **56**, 143–151 (2024).

90. E. Šturms, *Die Steinzeitlichen Kulturen Des Baltikums* (Habelt, 1970). [The Stone Age Cultures of the Baltic States].

91. A. Butrimas, "Corded Pottery Culture graves from Lithuania," in *Die Kontinentaleuropäischen Gruppen Der Kultur Mit Schnurkeramik. Schnurkeramik Symposium 1990*, M. Buchvaldek, C. Strahm, Eds. (Universita Karlova, 1992), pp. 307–311.

92. I. Loze, "Corded Pottery Culture in Latvia," in *Die Kontinentaleuropäischen Gruppen Der Kultur Mit Schnurkeramik. Schnurkeramik Symposium 1990*, M. Buchvaldek, C. Strahm, Eds. (Universita Karlova, 1992), pp. 313–320.

93. A. Girininkas, Migraciniai procesai Rytų Pabaltijyje vėlyvajame neolite. Virvelinės keramikos kultūra. *Lietuvos Archeologija* **23**, 73–92 (2002). [Migration processes in the Eastern Baltic in the Late Neolithic].

94. T. Edgren, *Studier Över Den Snörkeramiska Kulturens Keramik i Finland* (Suomen Muinaismuistoyhdistys, 1970). [Studies on the Ceramics of the Snörk Ware Culture in Finland].

95. R. Rimantienė, "Die Haffküstenkultur in Litauen," in *Die Kontinentaleuropäischen Gruppen Der Kultur Mit Schnurkeramik. Schnurkeramik Symposium 1990*, M. Buchvaldek, C. Strahm, Eds. (Universita Karlova, 1992), pp. 301–305. [The lagoon culture in Lithuania].

96. L. Kilian, *Haffküstenkultur Und Ursprung Der Balten* (Habelt, 1955). [Lagoon Coast Culture and the Origin of the Balts].

97. G. Piličiauskas, C. Heron, Aquatic radiocarbon reservoir offsets in the southeastern baltic. *Radiocarbon* **57**, 539–556 (2015).

98.. Pospieszny, Freshwater reservoir effect and the radiocarbon chronology of the cemetery in Ząbie, Poland. *J. Archaeol. Sci.* **53**, 264–276 (2015).

99. J. Meadows, V. Bērziņš, U. Brinker, H. Lübke, U. Schmölcke, A. Staude, I. Zagorska, G. Zariņa, Dietary freshwater reservoir effects and the radiocarbon ages of prehistoric human bones from Zvejnieki, Latvia. *J. Archaeol. Sci. Rep.* **6**, 678–689 (2016).

100. E. Holmqvist, Å. M. Larsson, A. Kriiska, V. Palonen, P. Pesonen, K. Mizohata, P. Kouki, J. Räisänen, Tracing grog and pots to reveal neolithic Corded Ware Culture contacts in the Baltic Sea region (SEM-EDS, PIXE). *J. Archaeol. Sci.* **91**, 77–91 (2018).

101. M. Ahola, V. Heyd, The Northern Way: Graves and funerary practices in Corded Ware Finland. *Praehistorische Zeitschrift* **95**, 78–111 (2020).

102. S. Vanhanen, A. Kriiska, K. Nordqvist, Corded Ware culture plant gathering at the Narva-Jõesuu IIB settlement and burial site in Estonia. *Environ. Archaeol.* **30**, 341–353 (2023).

103. N. Grasis, The Skaistkalnes Selgas double burial and the Corded Ware/Rzucewo culture: A model of the culture and the development of burial practices. *Lietuvos Archeologija* **31**, 39–70 (2007).

104. G. Piličiauskas, *Virvelinės Keramikos Kultūra Lietuvoje 2800-2400 Cal BC* (Lietuvos Istorijos Institutas, 2018). [Corded Pottery Culture in Lithuania 2800-2400 Cal BC].

105. A. Kriiska, K. Nordqvist, E. Jerem, W. Meid, "Estonian Corded Ware culture (2800–2000 cal. BC) – Defining a regional group in the eastern Baltic," in *Yamnaya Interactions: Proceedings of the International Workshop Held in Helsinki, 25–26 April 2019*, V. Heyd, Ed. (Archaeolingua, 2021), vol. 44, pp. 478–485.

106. L. Varul, R. M. Galeev, A. A. Malytina, M. Tõrv, S. V. Vasilyev, L. Lõugas, A. Kriiska, Complex mortuary treatment of a Corded Ware culture individual from the Eastern Baltic: A case study of a secondary deposit in Sope, Estonia. *J. Archaeol. Sci. Rep.* **24**, 463–472 (2019).

107. L. Lõugas, A. Kriiska, L. Maldre, New dates for the late neolithic Corded Ware culture burials and early husbandry in the East baltic region. *Archaeofauna* **16**, 21–31 (2007).

108. I. Antanaitis-Jacobs, M. Richards, L. Daugnora, R. Jankauskas, N. Ogrinc, Diet in early Lithuanian prehistory and the new stable isotope evidence. *Archaelogia Baltica* **12**, 12–30 (2009).

109. P. Pesonen, Å. M. Larsson, E. Holmqvist, The chronology of Corded Ware culture in finland - reviewing new data. *Fennoscandia Archaeologica* **36**, 130–141 (2019).

110. A. Kriiska, K. Nordqvist, D. V. Gerasimov, S. Sandell, Preliminary results of the research at Corded Ware sites in the Narva-Luga interfluve, Estonian-Russian border area in 2008-2014. *Archeol. Fieldw. Estonia* , 39–50 (2014).

111. W. Gumiński, T. Kowalski, "Aby na górce. Dwa późnoneolityczne groby z Dudki w Krainie Wielkich Jezior Mazurskich," in *Kurhany i Obrządek Pogrzebowyw IV-II Tysiącleciu p.n.e.*, H. Kowaleska-Marszałek, P. Włodarczak, Eds. (Instytut Archeologii i Etnologii Polskiej Akademii Nauk, Instytut Archeologii Uniwersytetu Warszawskiego, 2011), pp. 467–497. [Aby on the hill. Two late Neolithic graves from Dudka in the Masurian Lake District.].

112. J. Machnik, Der Stand der Erforschung der schnurkeramischen Gruppen im Gebiet der VR Polen. *Jahresschrift für Mitteldeutsche Vorgeschichte* **64**, 189–210 (1981). [The state of research into the Corded Ware groups in the territory of the People's Republic of Poland].

113. A. Häusler, Bemerkungen zu den östlichen Regionalgruppen der schnurkeramischen Becherkulturen. *Jahresschrift für Mitteldeutsche Vorgeschichte* **94**, 83–123 (2014). [Remarks on the eastern regional groups of the Corded Ware Beaker cultures].

114. J. Machnik, J. Bagińska, W. Koman, "Nowa, synkretyczna grupa kultury ceramiki sznurowej w Polsce środkowo-wschodniej," in *Problemy Epoki Kamienia Na Obszarze Starego Świata. Księga Jubileuszowa Dedykowana Profesorowi Januszowi K. Kozłowskiemu*, B. Ginter, B. Drobniewicz, B. Kazior, M. Nowak, M. Poltowicz, Eds. (Uniwersytet Jagielloński, Instytut Archeologii, 2001), pp. 389–399 [A new, syncretic group of the Corded Ware culture in east-central Poland].

115. M. Moskal-Del Hoyo, M. Krąpiec, B. Niezabitowska-Wiśniewska, The chronology of Site 3 in Ulów (Tomaszów Lubelski District, East Poland): The relevance of anthracological analysis for radiocarbon dating at a multicultural site. *Radiocarbon* **59**, 1399–1413 (2017).

116. P. Jarosz, P. Włodarczak, "Chronometria cmentarzysk kultury ceramiki sznurowej w Małopolsce," in *Społeczności Schyłkowego Eneolitu w Południowo-Wschodniej Polsce w Świetle Badań Archeologicznych i Analiz Interdyscyplinarnych*, A. Szczepanek, P. Jarosz, J. Libera, P. Włodarczak, Eds. (Wydawnictwo i Pracownia Archeologiczna Profil-Archeo, 2022), pp. 9–43. [Chronometry of Corded Ware culture cemeteries in Lesser Poland].

117. P. Włodarczak, "Eastern impulses in cultural and demographic change during the end of the south-eastern Polish Eneolithic," in *Yamnaya Interactions: Proceedings of the International Workshop Held in Helsinki, 25–26 April 2019*, V. Heyd, G. Kulcsár, B. Preda-Bălănică, Eds. (Archaeolingua, 2021), pp. 435–461.

118. P. Jarosz, P. Włodarczak, Chronologia bezwzględna kultury ceramiki sznurowej w Polsce południowo-wschodniej oraz na Ukrainie. *Przegląd Archeologiczny* **55**, 71–108 (2007). [Absolute chronology of the Corded Ware culture in southeastern Poland and Ukraine].

119. J. Machnik, J. Bagińska, W. Koman, *Neolityczne Kurhany Na Grzędzie Sokalskiej w Świetle Badań Archeologicznych w Latach 1988–2006* (Polska Akademia Umiejętności, 2009). [Neolithic Barrows at Grzęda Sokalska in the Light of Archaeological Research in the Years 1988–2006].

120. P. Włodarczak, "Battle-axes and beakers. The Final Eneolithic societies," in *The Past Societies 2: 5500 - 2000 BC. Polish Lands from the First Evidence of Human Presence to the Early Middle Ages*, P. Urbańczyk, Ed. (Sowa Sp. z o.o., 2017), pp. 276–336.

121. A. Kośko, P. Włodarczak, A final eneolithic research inspirations: Subcarpathia borderlands between Eastern and Western Europe. *Balt. Pontic Stud.* **23**, 259–291 (2018).

122. J. Machnik, Radiocarbon chronology of the Corded Ware culture on Grzeda Sokalska. A Middle Dnieper traits perspective. *Balt. Pontic Stud.* **7**, 221–250 (1999).

123. P. Włodarczak, Chronologia absolutna grupy krakowsko-sandomierskiej kultury ceramiki sznurowej na podstawie danych z cmentarzyska w Żernikach Górnych. *Sprawozdania Archeologiczne* **50**, 31–54 (1998). [Absolute chronology of the Kraków-Sandomierz Corded Ware culture group based on data from the cemetery in Żerniki Górne].

124. A. Linderholm, G. M. Kılınç, A. Szczepanek, P. Włodarczak, P. Jarosz, Z. Belka, J. Dopieralska, K. Werens, J. Górski, M. Mazurek, M. Hozer, M. Rybicka, M. Ostrowski, J. Bagińska, W. Koman, R. Rodríguez-Varela, J. Storå, A. Götherström, M. Krzewińska, Corded Ware cultural complexity uncovered using genomic and isotopic analysis from south-eastern Poland. *Sci. Rep.* **10**, 6885 (2020).

125. P. Włodarczak, Chronometry of the final eneolithic Cemeteries at Święte, Jarosław district, from the perspective of cultural relations among lesser Poland, Podolia and the North-Western Black Sea region. *Balt. Pontic Stud.* **23**, 178–212 (2018).

126. P. Włodarczak, Radiocarbon and dendrochronological dates of the corded ware culture. *Radiocarbon* **51**, 737–749 (2009).

127. I. Lazaridis, N. Patterson, D. Anthony, L. Vyazov, R. Fournier, H. Ringbauer, I. Olalde, A. A. Khokhlov, E. P. Kitov, N. I. Shishlina, S. C. Ailincăi, D. S. Agapov, S. A. Agapov, E. Batieva, B. Bauyrzhan, Z. Bereczki, A. Buzhilova, P. Changmai, A. A. Chizhevsky, I. Ciobanu, M. Constantinescu, M. Csányi, J. Dani, P. K. Dashkovskiy, S. Évinger, A. Faifert, P. N. Flegontov, A. Frînculeasa, M. N. Frînculeasa, T. Hajdu, T. Higham, P. Jarosz, P. Jelínek, V. I. Khartanovich, E. N. Kirginekov, V. Kiss, A. Kitova, A. V Kiyashko, J. Koledin, A. Korolev, P. Kosintsev, G. Kulcsár, P. Kuznetsov, R. Magomedov, M. A. Malikovich, E. Melis, V. Moiseyev, E. Molnár, J. Monge, O. Negrea, N. A. Nikolaeva, M. Novak, M. Ochir-Goryaeva, G. Pálfi, S. Popovici, M. P. Rykun, T. M. Savenkova, V. P. Semibratov, N. N. Seregin, A. Šefčáková, M. R. Serikovna, I. Shingiray, V. N. Shirokov, A. Simalcsik, K. Sirak, K. N.

Solodovnikov, J. Tárnoki, A. A. Tishkin, V. Trifonov, S. Vasilyev, A. Akbari, E. S. Brielle, K. Callan, F. Candilio, O. Cheronet, E. Curtis, O. Flegontova, L. Iliev, A. Kearns, D. Keating, A. M. Lawson, M. Mah, A. Micco, M. Michel, J. Oppenheimer, L. Qiu, J. Noah Workman, F. Zalzala, A. Szécsényi-Nagy, P. F. Palamara, S. Mallick, N. Rohland, R. Pinhasi, D. Reich, The genetic origin of the Indo-Europeans. bioRxiv 589597 [Preprint] (2024). https://doi.org/10.1101/2024.04.17.589597.

128. J. Machnik, *Studia Nad Kulturą Ceramiki Sznurowej w Małopolsce* (Ossolineum, 1966). [Studies on the Culture of Corded Ware in Lesser Poland].

129. P. Włodarczak, *Kultura Ceramiki Sznurowej Na Wyżynie Małopolskiej* (Instytut Archeologii i Etnologii Polskiej Akademii Nauk, 2006). [Corded Ware Culture in the Lesser Poland Upland].

130. J. Kruk, *Studia Osadnicze Nad Neolitem Wyżyn Lessowych* (Wydawnictwo Polskiej Akademii Nauk, 1973). [Settlement Studies on the Neolithic of the Loess Uplands].

131. A. Kempisty, P. Włodarczak, *Cemetery of the Corded Ware Culture in Żerniki Górne* (Institute of Archaeology, Warsaw University, 2000).

132. J. Gancarski, A. Machnikowie, J. Machnik, Wyniki badań kurhanu A kultury ceramiki sznurowej we wsi Bierówka, gmina Jasło, w województwie krośnieńskim. *Acta Archaeologica Carpathica* **25**, 57–87 (1986). [Results of research on barrow A of the Corded Ware culture in the village of Bierówka, Jasło commune, Krosno voivodeship].

133. K. Tunia, P. Włodarczak, Radiocarbon results for the Corded Ware culture from Southern Poland. *Przeglad Archeologiczny* **50**, 45–55 (2002).

134. P. Włodarczak, Cemetery of the Corded Ware Culture in Zielona, Koniusza commune, Małopolska. *Sprawozdania Archeologiczne* **56**, 307–344 (2004).

135. P. Włodarczak, "Grób 15 z Wilczyc na tle środkowoeuropejskim: Odmienność i reguła w rytuale pogrzebowym małopolskiej kultury ceramiki sznurowej [Grave 15 from Wilczyce against the Central European background: Distinctness and rules in burial rites of the

Corded Ware Culture in Lesser Poland],” in *Wilczyce, Stanowisko 10. Norma i Precedens w Rytuale Pogrzebowym Małopolskiej Kultury Ceramiki Sznurowej*, P. Włodarczak, Ed. (Wydawnictwo i Pracownia Archeologiczna PROFIL-ARCHEO, Instytut Archeologii i Etnologii PAN, 2019), pp. 169–209.

136. J. Machnik, E. Sosnowska, Starożytna mogiła z początku III tysiąclecia przed Chrystusem, ludności kultury ceramiki sznurowej w Średniej, gm. Krzywcza. *Rocznik Przemyski* **32**, 3–28 (1996). [An ancient grave from the beginning of the 3rd millennium BC, belonging to the Corded Ware culture in Średnia, Krzywcza commune].

137. M. Hozer, J. Machnik, A. Bajda-Wesołowska, “Groby kultury ceramiki sznurowej i domniemane kultury mierzanowickiej w Szczytnej, pow. Jarosław – źródła, analizy, wnioski,” in *Nekropolie Ludności Kultury Ceramiki Sznurowej z III Tysiąclecia Przed Chr. w Szczytnej Na Wysoczyźnie Kańczuckiej*, P. Jarosz, J. Machnik, Eds. (Fundacja Rzeszowskiego Ośrodka Archeologicznego, 2017), pp. 7–130. [Graves of the Corded Ware culture and alleged Mierzanowice culture in Szczytna, Jarosław district – sources, analyses, conclusions].

138. J. Machnik, H. Mačalova, P. Jarosz, P. Włodarczak, “Kurhan nr 2 kultury ceramiki sznurowej w miejscowości Hankovce okr. Bardejov,” in *Archaeology and Natural Background of the Lower Beskid Mountains, Carpathians. Part 2.*, J. Machnik, Ed. (Polska Akademia Umiejętności, 2008), pp. 193–240. [Barrow No. 2 of the Corded Ware culture in Hankovce, Bardejov district].

139. J. Machnik, H. Mačalova, K. Tunia, P. Jarosz, “Kurhan nr 34 kultury ceramiki sznurowej w miejscowości Hankovce, okr. Bardejov, stanowisko 1,” in *Archaeology and Natural Background of the Lower Beskid Mountains, Carpathians. Part 2*. Kurimská Brázda, J. Machnik, Ed. (Polska Akademia Umiejętności, 2008), pp. 157–186. [Barrow No. 34 of the Corded Ware culture in Hankovce, Bardejov district, site 1].

140. J. Gancarski, A. Machnikowie, J. Machnik, Kurhan B kultury ceramiki sznurowej w Bierówce, gmina Jasło, w świetle badań wykopaliskowych. *Acta Archaeologica Carpathica* **29**, 99–124 (1990). [Barrow B of the Corded Ware culture in Bierówka, Jasło commune, in the light of excavations].

141. L. Šebela, *The Corded Ware Culture in Moravia and in the Adjacent Part of Silesia* (Archeologický ústav akademie věd České Republiky v Brně, 1999).

142. M. Buchvaldek, Zum gemeineuropäischen Horizont der Schnurkeramik. *Praehistorische Zeitschrift* **61**, 129–151 (1986). [On the common European horizon of the Corded Ware culture].

143. J. Peška, "Relative and absolute chronology of burial of Corded Ware culture in Moravia," in *Moving Northward: Professor Volker Heyd's Festschrift as He Turns 60*, A. Lahelma, M. Lavento, K. Mannermaa, M. Ahola, E. Holmqvist, K. Nordqvist, Eds. (The Archaeological Society of Finland, 2023), pp. 115–131.

144. J. Baron, M. Furmanek, A. Hałuszko, B. Kufel-Diakowska, Differentiation of burial practices in the Corded Ware Culture. The example of the Magnice site in SW Poland. *Praehistorische Zeitschrift* **93**, 169–184 (2019).

145. J. Peška, Nová pohřebiště kultury se šňůrovou keramikou na střední Moravě (Dosavadní výsledky horizontální stratigrafie) [Neue Gräberfelder der Schnurkeramikkultur in Mittelmähren (Bisherige Ergebnisse der Horizontalen Stratigrafie)]. *Prehled Vyzkumu* **58**, 19–47 (2017). [New burial sites of the Corded Ware culture in Central Moravia (So far results of horizontal stratigraphy)].

146. J. Peška, "Kultura se šňůrovou keramikou na Moravě v kontrapozici radiokarbonového datování," in *Mente et Retro. Studia Archaeologica Johanni Machnik Viro Doctissimo Octogesimo Vitae Ab Amicis, Collegis et Discipulis Oblata*, S. Csopek, S. Kadrow, Eds. (Institutum Archaeologicum Universitatis Ressoviensis, 2010), pp. 247–273. [Corded Ware Culture in Moravia in Contrast with Radiocarbon Dating].

147. I. Rakovský, L. Šebela, Hroby se šňůrovou keramikou v Pavlově. *Archeologické Rozhledy* **43**, 206–224 (1991). [Graves with corded pottery in Pavlov].

148. P. Fojtík, Držovice (k. ú. Držovice na Moravě, okr. Prostějov). „Pastviska". KŠK. Pohřebiště. Záchranný archeologický výzkum. *Prehled Vyzkumu* **57**, 186–187 (2015). [Rescue archaeological research].

149. H. Rathmann, J. Wahl, “Strikte Regel oder strittige Richtlinie? Eine kritisch Betrachtung der geschlechtsdifferentzierten Seitenlage der Schnurkeramiker,” in *Der Zahn Der Zeit. Mensch Und Kultur Im Spiegel Interdiziplinärer Forschung. Festschrift Für Kurt W. Alt*, C. Meyer, P. Held, C. Knipper, N. Nicklisch, Eds. (Landesamt für Denkmalpflege und Archäologie Sachsen-Anhalt, 2020), pp. 47–59. [Strict rule or controversial guideline? A critical examination of the gender-differentiated lateral position of the Corded Ware culture].

150. M. Dobeš, M. Pecinovská, M. Ernée, V. Heyd, “On the earliest Corded Ware in Bohemia,” in *Yamnaya Interactions: Proceedings of the International Workshop Held in Helsinki, 25-26 April 2019*, V. Heyd, G. Kulcsár, B. Preda-Bălănică, Eds. (Archaeolingua, 2021), vol. 2.

151. J. Kolář, “Idealized World or Real Society? Social Patterns of Corded Ware Culture in Moravia (Czech Republic),” in *Transitional Landscapes? The 3rd Millennium BC in Europe. Proceedings of the International Workshop “Socio-Environmental Dynamics over the Last 12,000 Years: The Creation of Landscapes III (15th - 18th April 2013)” in Kiel*, M. Furholt, R. Grossmann, M. Szmyt, Eds. (Dr. Rudolf Habelt GmbH, 2016), pp. 191–207.

152. B. Gworys, J. Rosińczuk-Tonderys, A. Chrószcz, M. Janeczek, A. Dwojak, J. Bazan, M. Furmanek, T. Dobosz, M. Bonar, A. Jonkisz, I. Całkosiński, Assessment of late Neolithic pastoralist’s life conditions from the Wroclaw–Jagodno site (SW Poland) on the basis of physiological stress markers. *J. Archaeol. Sci.* **40**, 2621–2630 (2013).

153. J. Peška, “Vybrané formy eneolitických pásových zápon v Evropě – Die ausgewählten Formen der äneolithischenGürtelhaken in Europa,” in *Otázky Neolitu a Eneolitu Našich Krajín –2001. Zborník Referátov z 20. Pracovného Stretnutia Bádateľov Pre Výskum Neolitu a Eneolitu Čiech, Moravy a Slovenska. Liptovská Sielnica 9.–12.10.2001*, I. Cheben, I. Kuzma, Eds. (Archeologický Ústav Slovenskej Akad. Vied, 2002), pp. 259–281. [Selected forms of Eneolithic belt buckles in Europe – Die ausgewählten Formen der äneolithischen Gürtelhaken in Europa].

154. M. Dobeš, P. Limburský, *Pohřebiště Staršího Eneolitu a Šňůrové Keramiky ve Vliněvsi – Gräberfeld Des Älteren Äneolithikums Und Der Schnurkeramik in Vliněves* (Vydal

Archeologický ústav (Akademie věd České republiky, 2013). [The burial ground of the Early Eneolithic and Corded Ware in Vliněves].

155. V. M. Narasimhan, N. Patterson, P. Moorjani, N. Rohland, R. Bernardos, S. Mallick, I. Lazaridis, N. Nakatsuka, I. Olalde, M. Lipson, A. M. Kim, L. M. Olivieri, A. Coppa, M. Vidale, J. Mallory, V. Moiseyev, E. Kitov, J. Monge, N. Adamski, N. Alex, N. Broomandkhoshbacht, F. Candilio, K. Callan, O. Cheronet, B. J. Culleton, M. Ferry, D. Fernandes, S. Freilich, B. Gamarra, D. Gaudio, M. Hajdinjak, E. Harney, T. K. Harper, D. Keating, A. M. Lawson, M. Mah, K. Mandl, M. Michel, M. Novak, J. Oppenheimer, N. Rai, K. Sirak, V. Slon, K. Stewardson, F. Zalzala, Z. Zhang, G. Akhatov, A. N. Bagashev, A. Bagnera, B. Baitanayev, J. Bendezu-Sarmiento, A. A. Bissembaev, G. L. Bonora, T. T. Chargynov, T. Chikisheva, P. K. Dashkovskiy, A. Derevianko, M. Dobeš, K. Douka, N. Dubova, M. N. Duisengali, D. Enshin, A. Epimakhov, A. V. Fribus, D. Fuller, A. Goryachev, A. Gromov, S. P. Grushin, B. Hanks, M. Judd, E. Kazizov, A. Khokhlov, A. P. Krygin, E. Kupriyanova, P. Kuznetsov, D. Luiselli, F. Maksudov, A. M. Mamedov, T. B. Mamirov, C. Meiklejohn, D. C. Merrett, R. Micheli, O. Mochalov, S. Mustafokulov, A. Nayak, D. Pettener, R. Potts, D. Razhev, M. Rykun, S. Sarno, T. M. Savenkova, K. Sikhymbaeva, S. M. Slepchenko, O. A. Soltobaev, N. Stepanova, S. Svyatko, K. Tabaldiev, M. Teschler-Nicola, A. A. Tishkin, V. V. Tkachev, S. Vasilyev, P. Veleminsky, D. Voyakin, A. Yermolayeva, M. Zahir, V. S. Zubkov, A. Zubova, V. S. Shinde, C. Lalueza-Fox, M. Meyer, D. Anthony, N. Boivin, K. Thangaraj, D. J. Kennett, M. Frachetti, R. Pinhasi, D. Reich, The formation of human populations in South and Central Asia. *Science* **365**, eaat7487 (2019).

156. J. Jacobs, *Die Einzelgrabkultur in Mecklenburg-Vorpommern* (Archäologisches Landesmuseum Mecklenburg-Vorpommern, 1991). [The single grave culture in Mecklenburg-Western Pomerania].

157. R. Schroeder, *Die Nordgruppe Der Oderschnurkeramik* (Walter de Gruyter & Co., 1951). [The Northern Group of the Oderschnurkeramik].

158. J. Czebreszuk, *Społeczności Kujaw w Początkach Epoki Brązu* (Uniwersytet im. Adama Mickiewicza w Poznaniu, 1996). [Communities of Kujawy in the Early Bronze Age].

159. J. Czebreszuk, M. Szmyt, Der Epochenumbruch vom Neolithikum zur Bronzezeit im Polnischen Tiefland am Beispiel Kujawiens. *Praehistorische Zeitschrift* **73**, 167–232 (1998). [The epochal change from the Neolithic to the Bronze Age in the Polish Lowland using the example of Kuyavia].

160. M. Furholt, "Materielle Kultur und räumliche Strukturen sozialer Identität im 4. und 3. Jt. v. Chr. in Mitteleuropa. Eine methodische Skizze," in *Sozialarchäologische Perspektiven: Gesellschaftlicher Wandel 5000-1500 v. Chr. Zwischen Atlantik Und Kaukasus*, S. Hansen, J. Müller, Eds. (Philipp von Zabern, 2011), pp. 243–26. [Material culture and spatial structures of social identity in the 4th and 3rd millennium BC in Central Europe. A methodological outline].

161. L. Pospieszny, *Zwyczaje Pogrzebowe Społeczności Kultury Ceramiki Sznurowej w Wielkopolsce i Na Kujawach [Mortuary Practices of the Corded Ware Culture Societies in Greater Poland and Cuiavia]* (Uniwersytet im. Adama Mickiewicza w PoznaniuInstytut Prahistorii, 2009).

162. J. Czebreszuk, M. Szmyt, "Identities, Differentiation and Interactions on the Central European Plain in the 3rd millennium BC," in *Sozialarchäologische Perspektive: Gesellschaftlicher Wandel 5000-1500 v. Chr. Zwichen Atlantik Und Kaukasus*, S. Hansen, J. Müller, Eds. (Philipp von Zabern, 2011), pp. 269–291.

163. E. Niesiołowska, Materiały neolityczne ze stanowiska 6 w Pikutkowie, pow. Włocławek. *Seria Archeologiczna* **14**, 79–144 (1967). [Neolithic materials from site 6 in Pikutkowo, Włocławek district].

164. D. Forler, J.-P. Schmidt, *Bericht Über Die Archäologischen Untersuchungen Im Bereich Des Kiessandtagebaus Zarrenthin, Lkr. Demmin* (Landesamt für Kultur und Denkmalpflege Mecklenburg-Vorpommern, 2007). [Report on the archaeological investigations in the area of the Zarrenthin gravel and sand opencast mine, Demmin district].

165. J. Czebreszuk, J. Łoś, Grób kultury ceramiki sznurowej w Żernikach, gm. Kruszwica, pow. Inowrocław, woj. Kujawsko-pomorskie, stanowisko 27. *Sprawozdania Archeologiczne* **51**,

97–113 (1999). [Corded Ware culture grave in Żerniki, Kruszwica commune, Inowrocław district, Kuyavian-Pomeranian Voivodeship, site 27].

166. V. Dresely, *Schnurkeramik Und Schnurkeramiker Im Taubertal* (Theiss, 2004). [Corded Ware and Corded Ware Pottery in the Tauber Valley].

167. S. E. Ortolf, Das schnurkeramische Gräberfeld von Lauda-Königshofen im Taubertal. *Fundberichte aus Baden-Württemberg* **34**, 409–528 (2014). [The Corded Ware burial ground of Lauda-Königshofen in the Tauber Valley].

168. D. M. Fernandes, D. Strapagiel, P. Borówka, B. Marciniak, E. Żądzińska, K. Sirak, V. Siska, R. Grygiel, J. Carlsson, A. Manica, W. Lorkiewicz, R. Pinhasi, A genomic Neolithic time transect of hunter-farmer admixture in central Poland. *Sci. Rep.* **8**, 14879 (2018).

169. J. Czebreszuk, M. Szmyt, Osadnictwo z III i II tys. Przed Chr. na stanowiskach 1 i 24 w Głuchowie, woj. Wielkopolskie. *Fontes Archaeologici Posnanienses* **55**, 101–145 (2019). [BC at sites 1 and 24 in Głuchów, Greater Poland Voivodeship].

170. A. Selent, Zwei Körpergräber der spätneolithischen Einzelgrabkultur und ein eisenzeitliches Grubenhaus bei Pasewalk, Lkr. Vorpommern-Greifswald. *Bodendenkmalpflege Mecklenburg Vorpommern* **60**, 17–49 (2013). [Two inhumation graves of the Late Neolithic single-grave culture and an Iron Age pit house near Pasewalk, Vorpommern-Greifswald district].

171. A. Kośko, Pontic traits in the materials of the Kujawy Funnel Beaker Culture and Early Corded Ware Culture - a radiocarbon perspective. *Balt. Pontic Stud.* **7**, 203–210 (1999).

172. T. Goslar, A. Kośko, "Z badań nad chronologią i topogenezą kujawskich kurhanów starosznurowych. Krusza Zamkowa, powiat Inowrocław, stanowisko 3," in *Kurhany i Obrządek Pogrzebowy* w *IV-II Tysiącleciu p.n.e.*, H. Kowaleska-Marszałek, P. Włodarczak, Eds. (Instytut Archeologii i Etnologii Polskiej Akademii Nauk, Instytut Archeologii Uniwersytetu Warszawskiego, 2011), pp. 407–415. [From the study of the chronology and topogenesis of the Kujawy Old Corded Barrows. Krusza Zamkowa, Inowrocław County, site 3].

173. D. Jankowska, A. Kośko, K. Siuchniński, H. Quitta, G. Kohl, Untersuchungen zur Chronologie der neolithischen Kulturen im Polnischen Tiefland. *Zeitschrift für Archäologie* **13**, 219–240 (1979). [Investigations into the chronology of Neolithic cultures in the Polish Lowland].

174. J. Czebreszuk, M. Szmyt, "The 3rd Millennium BC in Kujawy in the Light of 14-C Dates," in *Die Absolute Chronologie in Mitteleuropa 3000-2000 v. Chr. [The Absolute Chronology of Central Europe 3000-2000 v. Chr.]*, J. Czebreszuk, J. Müller, Eds. (Instytut Prahistorii Uniwersytetu im. Adama Mickiewicza, Verlag Marie Leidorf GmbH, 2001), pp. 177–208.

175. K. Rassmann, *Spätneolithikum Und Frühe Bronzezeit Im Flachland Zwischen Elbe Und Oder* (Archäologischen Landesmuseum für Mecklenburg-Vorpommern & Archäologischen Gesellschaft für Mecklenburg und Vorpommern, 1993). [Late Neolithic and Early Bronze Age in the lowlands between the Elbe and Oder].

176. T. Terberger, J. Pieck, Zur absoluten Chronologie der Steinzeit in Mecklenburg-Vorpommern. *Bodendenkmalpflege Mecklenburg-Vorpommern* **1997**, 7–39 (1998). [On the absolute chronology of the Stone Age in Mecklenburg-Western Pomerania].

177. C. V. Steinmann, Im Osten was Neues. Der Übergang vom Mittel- zum Spätneolithikum in Mecklenburg. *Varia Neolithica* **3**, 117–126 (2004). [The transition from the Middle to the Late Neolithic in Mecklenburg].

178. K. Rassmann, "Zur absoluten Chronologie des ausgehenden Neolithikums im nördlichen Mitteleuropa und Südskandinavien," in *Die Absolute Chronologie in Mitteleuropa 3000-2000 v. Chr. [The Absolute Chronology of Central Europe 3000-2000 v. Chr.]*, J. Czebreszuk, J. Müller, Eds. (Instytut Prahistorii Uniwersytetu im. Adama Mickiewicza, Verlag Marie Leidorf GmbH, 2001), pp. 271–286. [On the absolute chronology of the late Neolithic in northern Central Europe and southern Scandinavia].

179. I. Mathieson, I. Lazaridis, N. Rohland, S. Mallick, N. Patterson, S. A. Roodenberg, E. Harney, K. Stewardson, D. Fernandes, M. Novak, K. Sirak, C. Gamba, E. R. Jones, B. Llamas, S. Dryomov, J. Pickrell, J.-L. Arsuaga, J. M. B. de Castro, E. Carbonell, F. Gerritsen, A. Khokhlov, P. Kuznetsov, M. Lozano, H. Meller, O. Mochalov, V. Moiseyev,

M. A. R. Guerra, J. Roodenberg, J. M. Vergès, J. Krause, A. Cooper, K. W. Alt, D. Brown, D. Anthony, C. Lalueza-Fox, W. Haak, R. Pinhasi, D. Reich, Genome-wide patterns of selection in 230 ancient Eurasians. *Nature* **528**, 499–503 (2015).

180. I. Mathieson, S. Alpaslan-Roodenberg, C. Posth, A. Szécsényi-Nagy, N. Rohland, S. Mallick, I. Olalde, N. Broomandkhoshbacht, F. Candilio, O. Cheronet, D. Fernandes, M. Ferry, B. Gamarra, G. G. Fortes, W. Haak, E. Harney, E. Jones, D. Keating, B. Krause-Kyora, I. Kucukkalipci, M. Michel, A. Mittnik, K. Nägele, M. Novak, J. Oppenheimer, N. Patterson, S. Pfrengle, K. Sirak, K. Stewardson, S. Vai, S. Alexandrov, K. W. Alt, R. Andreescu, D. Antonović, A. Ash, N. Atanassova, K. Bacvarov, M. B. Gusztáv, H. Bocherens, M. Bolus, A. Boroneanţ, Y. Boyadzhiev, A. Budnik, J. Burmaz, S. Chohadzhiev, N. J. Conard, R. Cottiaux, M. Čuka, C. Cupillard, D. G. Drucker, N. Elenski, M. Francken, B. Galabova, G. Ganetsovski, B. Gély, T. Hajdu, V. Handzhyiska, K. Harvati, T. Higham, S. Iliev, I. Janković, I. Karavanić, D. J. Kennett, D. Komšo, A. Kozak, D. Labuda, M. Lari, C. Lazar, M. Leppek, K. Leshtakov, D. L. Vetro, D. Los, I. Lozanov, M. Malina, F. Martini, K. McSweeney, H. Meller, M. Menđušić, P. Mirea, V. Moiseyev, V. Petrova, T. D. Price, A. Simalcsik, L. Sineo, M. Šlaus, V. Slavchev, P. Stanev, A. Starović, T. Szeniczey, S. Talamo, M. Teschler-Nicola, C. Thevenet, I. Valchev, F. Valentin, S. Vasilyev, F. Veljanovska, S. Venelinova, E. Veselovskaya, B. Viola, C. Virag, J. Zaninović, S. Zäuner, P. W. Stockhammer, G. Catalano, R. Krauß, D. Caramelli, G. Zariņa, B. Gaydarska, M. Lillie, A. G. Nikitin, I. Potekhina, A. Papathanasiou, D. Borić, C. Bonsall, J. Krause, R. Pinhasi, D. Reich, The genomic history of southeastern Europe. *Nature* **555**, 197–203 (2018).

181. W. Haak, M. Furholt, M. Sikora, A. Ben Rohrlach, L. Papac, K.-G. Sjögren, V. Heyd, M. F. Mortensen, A. B. Nielsen, J. Müller, I. Feeser, G. Kroonen, K. Kristiansen, "The Corded Ware Complex in Europe in Light of Current Archaeogenetic and Environmental Evidence," in *The Indo-European Puzzle Revisited: Integrating Archaeology, Genetics, and Linguistics*, K. Kristiansen, G. Kroonen, E. Willerslev, Eds. (Cambridge Univ. Press, 2023), pp. 63–80; www.cambridge.org/core/product/465553463037195D74FA9148A733683D.

182..M Larsson, *Breaking and Making Bodies and Pots: Material and Ritual Practices in Sweden in the Third Millennium BC* (Department of Archaeology and Ancient History, 2009).

183. M. Malmer, *The Neolithic of South Sweden: TRB, GRK, and STR* (Akademibokhandelsgruppen AB, 2002).

184. E. Fornander, Dietary diversity and moderate mobility – isotope evidence from Scanian battle axe culture burials. *J. Nordic Archaeol. Sci.* **18**, 13–29 (2013).

185. D. Olausson, "Burial in the Swedish-Norwegian Battle Axe Culture: Questioning the myth of homogeneity," in *Neolithic Diversities: Perspectives from a Conference in Lund, Sweden*, K. Brink, S. Hydén, K. Jennbert, L. Larsson, D. Olausson, Eds. (Department of Archaeology and Ancient History, 2015), pp. 98–106.

186. M. Malmer, *Jungneolithische Studien* (CWK Gleerups Forlag, 1962). [Late Neolithic studies].

187. R. Edenmo, *Prestigeekonomi under Yngre Stenåldern: Gåvoutbyten Och Regionala Identiteten i Den Svenska Båtyxekulturen* (Department of Archaeology and Ancient History, 2008). [Prestige Economy during the Neolithic Age: Gift Exchanges and Regional Identity in the Swedish Boat Axe Culture].

188. A. Bach, H. Bach, Beiträge zur Kultur und Anthropologie der mitteldeutschen Schnurkeramik II. *Alt Thuring* **13**, 43–107 (1975). [Contributions to the culture and anthropology of the Central German Corded Ware II].

189. S. Bücke, H. J. Barthel, W. Gall, Beiträge zur Kultur der mitteldeutschen Schnurkeramik III. *Alt Thuring* **24**, 33–116 (1989). [Contributions to the culture of the Central German Corded Ware III].

190. R. Feustel, H. Bach, W. Gall, M. Teichert, Beiträge zur Kultur und Anthropologie der mitteldeutschen Schnurkeramiker. *Alt Thuring* **8**, 20–170 (1966). [Contributions to the culture and anthropology of the Central German Corded Ware people].

191. G. Loewe, *Kataloge Zur Mitteldeutschen Schnurkeramik Teil I: Thüringen* (VEB Niemeyer Verlag, 1959). [Catalogues of Central German Corded Ware Part I: Thuringia].

192. H. Lucas, *Kataloge Zur Mitteldeutschen Schnurkeramik Teil II: Saalemündungsgebiet* (VEB Deutscher Verlag der Wissenschaften, 1965). [Catalogues of Central German Corded Ware Part II: Saale estuary area].

193. W. Matthias, *Kataloge Zur Mitteldeutschen Schnurkeramik Teil III: Nordharzgebiet* (VEB Deutscher Verlag der Wissenschaften, 1968). [Catalogues of Central German Corded Ware Part III: Northern Harz Region].

194. W. Matthias, *Kataloge Zur Mitteldeutschen Schnurkeramik Teil IV: Südharz-Unstrut-Gebiet* (VEB Deutscher Verlag der Wissenschaften, 1974). [Catalogues of Central German Corded Ware Part IV: South Harz-Unstrut Region].

195. W. Matthias, *Kataloge Zur Mitteldeutschen Schnurkeramik Teil V: Mittleres Saalegebiet* (VEB Deutscher Verlag der Wissenschaften, 1982). [Catalogues of Central German Corded Ware Part V: Middle Saale Region].

196. W. Matthias, *Kataloge Zur Mitteldeutschen Schnurkeramik Teil VI: Restgebiete Und Nachträge* (VEB Deutscher Verlag der Wissenschaften, 1987). [Catalogues of Central German Corded Ware Part VI: Remaining Areas and Supplements].

197. M. Becker, M. Fröhlich, K. Balfanz, B. Kromer, R. Friederich, "Das 3. Jt. v. Chr. Zwischen Saale und Unstrut – Kulturelle Veränderungen im Spiegel der Radiokohlenstoffdatierung," in *2200 BC – Ein Klimasturz Als Ursache Für Den Zerfall Der Alten Welt? 7. Mitteldeutscher Archäologentag Vom 23. Bis 26. Oktober 2014 in Halle (Saale)*, H. Meller, H. W. Arz, R. Jung, R. Risch, Eds. (Landesamt für Denkmalpflege und Archäologie Sachsen-Anhalt, Landesmuseum für Vorgeschichte, 2015), pp. 715–745. [The 3rd millennium BC between the Saale and Unstrut rivers – Cultural changes reflected in radiocarbon dating].

198. J. Müller, C. Becker, H. Bruchhaus, E. Kaiser, A. Neubert, S. Pichler, M. Zabel, Radiokarbonchronologie - Keramiktechnologie - Osteologie - Anthropologie - Raumanalysen. Beiträge zum Neolithikum und zur Frühbronzezeit im Mittelelbe-Saale-Gebiet. *Bericht der Römisch Germanischen Kommission* **80**, 25–212 (2001).

[Radiocarbon chronology - Ceramic technology - Osteology - Anthropology - Spatial analysis].

199. H. Behrens, Ein neolithisches Bechergrab aus Mitteldeutschland mit beinerner Hammerkopfnadel und Kupfergerät. *Jahresschrift für Mitteldeutsche Vorgeschichte* **36**, 53–69 (1952). [A Neolithic beaker grave from Central Germany with a bone hammer-head needle and copper implement].

200. U. Fischer, *Die Gräber Der Steinzeit Im Saalegebiet. Studien Über Neolithische Und Frühbronzezeitliche Grab- Und Bestattungsformen in Sachsen-Thüringen* (Walter De Gruyter & Co., 1956). [Stone Age Graves in the Saale Region: Studies on Neolithic and Early Bronze Age Grave and Burial Forms in Saxony-Thuringia].

201. W. Haak, G. Brandt, H. N. de Jong, C. Meyer, R. Ganslmeier, V. Heyd, C. Hawkesworth, A. W. G. Pike, H. Meller, K. W. Alt, Ancient DNA, Strontium isotopes, and osteological analyses shed light on social and kinship organization of the Later Stone Age. *Proc. Natl. Acad. Sci. U.S.A.* **105**, 18226–18231 (2008).

202. M. Fröhlich, P. Becker, M. Becker, "Die endneolithische Mehrfachbestattung von Oechlitz, Saalekreis - Eine gemeinsame Grablege der Schnurkeramik - und Glockenbecherkultur," in *Neue Gleise Auf Alten Wegen II - Jüdendorf Bis Gröbers*, H. Meller, Ed. (Landesamt für Denkmalpflege und Archäologie Sachsen-Anhalt - Landesmuseum für Vorgeschichte, 2017), pp. 308–314. [The late Neolithic multiple burial of Oechlitz, Saalekreis - a common burial site of the Corded Ware and Bell Beaker cultures].

203. D. Menke, A. Moser, C. Schwerdtfeger, K. Schwerdtfeger, "Schmuckausstattung in schnurkeramischen Gräbern - einfacher Zierrat oder sinnstiftende Objekte? Versuch einer Interpration," in *Neue Gleise Auf Alten Wegen II - Jüdendorf Bis Gröbers*, H. Meller, Ed. (Landesamt für Denkmalpflege und Archäologie Sachsen-Anhalt, Landesmuseum für Vorgeschichte, 2017), pp. 274–291. [Ornaments in Corded Ware graves – simple ornaments or meaningful objects? An attempt at an interpretation].

204. H. Behrens, *Die Jungsteinzeit Im Mittelelbe-Saale-Gebiet* (VEB Deutscher Verlag der Wissenschaften, 1973). [The Neolithic Period in the Middle Elbe-Saale Region].

205. M. Szmyt, "In the far reaches of two worlds. On the study of contacts between the societies of the Globular Amphora and Yamnaya cultures," in *A Turning of Ages/ Im Wandel Der Zeiten. Jubilee Book Dedicated to Professor Jan Machnik on His 70th Anniversary*, S. Kadrow, Ed. (Institute of Archaeology and Ethnology Polish Academy of Sciences, 2000), pp. 443–466.

206. M. Szmyt, Between the seas: Baltic-Pontic contact space in the 3rd millennium BC. *Vita Antiqua* **10**, 155–164 (2018).

207. J. Müller, *Separation, Hybridisation, and Networks. Globular Amphora Sedentary Pastoralists ca. 3200–2700 BCE* (Sidestone Press, 2023).

208. M. Woidich, *Die Westliche Kugelamphorenkultur. Untersuchungen Zu Ihrer Raum-Zeitlichen Differenzierung, Kulturellen Und Anthropologischen Identität* (De Gruyter, 2014). [The Western Globular Amphora Culture: Investigations into its Spatio-Temporal Differentiation, Cultural and Anthropological Identity].

209. A. Mittnik, K. Massy, C. Knipper, F. Wittenborn, R. Friedrich, S. Pfrengle, M. Burri, N. Carlichi-Witjes, H. Deeg, A. Furtwängler, M. Harbeck, K. von Heyking, C. Kociumaka, I. Kucukkalipci, S. Lindauer, S. Metz, A. Staskiewicz, A. Thiel, J. Wahl, W. Haak, E. Pernicka, S. Schiffels, P. W. Stockhammer, J. Krause, Kinship-based social inequality in Bronze Age Europe. *Science* **366**, 731–734 (2019).

210. V. Heyd, *Die Spätkupferzeit in Süddeutschland* (Dr. Rudolf Habelt GmbH, 2000). [The Late Copper Age in Southern Germany].

211. L. Olerud, Reassessing the gender ideology of the supra-regional Corded Ware culture. *Kleos - Amsterdam Bull. Ancient Stud. Archaeol.* **4**, 10–42 (2021).

212. K. Massy, C. Knipper, A. Mittnik, S. Kraus, E. Pernicka, F. Wittenborn, J. Krause, P. W. Stockhammer, "Patterns of Transformation from the Final Neolithic to the Early Bronze Age: A Case Study from the Lech Valley South of Augsburg," in *Appropriating Innovations: Entangled Knowledge in Eurasia, 5000-1500 BCE*, P. W. Stockhammer, J. Maran, Eds. (Oxbow Books, 2017), pp. 241–261.

213. P. W. Stockhammer, K. Massy, C. Knipper, R. Friedrich, B. Kromer, S. Lindauer, J. Radosavljeví, F. Wittenborn, J. Krause, Rewriting the central european early bronze age chronology: Evidence from large-Scale radiocarbon dating. *PLOS ONE* **10**, e0139705 (2015).

214. K. G. Sjögren, I. Olalde, S. Carver, M. E. Allentoft, T. Knowles, G. Kroonen, A. W. G. Pike, P. Schroter, K. A. Brown, K. R. Brown, R. J. Harrison, F. Bertemes, D. Reich, K. Kristiansen, V. Heyd, Kinship and social organization in Copper Age Europe. A cross-disciplinary analysis of archaeology, DNA, isotopes, and anthropology from two Bell Beaker cemeteries. *PLOS ONE* **15**, e0241278 (2020).

215. C. Knipper, A. Mittnik, K. Massy, C. Kociumaka, I. Kucukkalipci, M. Maus, Female exogamy and gene pool diversification at the transition from the Final Neolithic to the Early Bronze Age in central Europe. *Proc. Natl. Acad. Sci. U.S.A.* **114**, 10083–10088 (2017).

216. A. Andrades Valtueña, A. Mittnik, F. M. Key, W. Haak, R. Allmäe, A. Belinskij, M. Daubaras, M. Feldman, R. Jankauskas, I. Janković, K. Massy, M. Novak, S. Pfrengle, S. Reinhold, M. Šlaus, M. A. Spyrou, A. Szécsényi-Nagy, M. Tõrv, S. Hansen, K. I. Bos, P. W. Stockhammer, A. Herbig, J. Krause, The stone age plague and its persistence in Eurasia. *Curr. Biol.* **27**, 3683–3691.e8 (2017).

217. E. Hübner, *Jungneolithische Gräber Auf Der Jütischen Halbinsel: Typologische Und Chronologische Studien Zur Einzelgrabkultur* (Nordiske Fortidsminder, Ser. B, 2005). [Late Neolithic graves on the Jutlandic Peninsula: Typological and chronological studies on the single-grave culture].

218. S. Schultrich, *Das Jungneolithikum in Schleswig-Holstein* (Sidestone Press, 2018). [The Late Neolithic in Schleswig-Holstein].

219. S. Müller, De jydske Enkeltgrave fra Stenalderen. *Aarboger for Nordisk Oldkyndighed og Historie* **2**, 157–282 (1898). [The Jutlandic single graves from the Stone Age].

220. P. V. Glob, Studier over den jyske Enkeltgravkultur. *Aarboger for Nordisk Oldkyndighed og Historie* **1944**, 1–283 (1945). [Studies on the Jutland Single Grave Culture].

221. K. W. Struve, *Die Einzelgrabkultur in Schleswig-Holstein Und Ihre Kontinentalen Beziehungen* (K. Wachholtz, 1955). [The Single Grave Culture in Schleswig-Holstein and its Continental Relations].

222. J. D. Van der Waals, W. Glasbergen, Beaker types and their distribution in the Netherlands. Intrusive types, mutual influences and local evolutions. *Palaeohistoria* **4**, 5–46 (1955).

223. J. N. Lanting, J. van der Plicht, De C14-chronologie van de Nederlandse pre- en protohistorie III: Neolithicum. *Palaeohistoria* **41**, 1–110 (2002).

224. H. Vandkilde, *From Stone to Bronze: The Metalwork of the Late Neolithic and Earliest Bronze Age in Denmark* (Jutland Archaeological Society, Moesgaard, 1996).

225. K. Ebbesen, *The Battle Axe Period / Stridsøksetid* (Forfatterforlaget ATTIKA, 2006).

226. E. Drenth, A. E. Lanting, De chronologie van de Enkelgrafcultuur in Nederland: Enkele voorlopige opmerkingen. *Paleo Aktueel* **2**, 42–46 (1991). [The chronology of the Single Grave Culture in the Netherlands: Some preliminary remarks].

227. A. F.-H. Egfjord, A. Margaryan, A. Fischer, K.-G. Sjögren, T. D. Price, N. N. Johannsen, P. O. Nielsen, L. Sørensen, E. Willerslev, R. Iversen, M. Sikora, K. Kristiansen, M. E. Allentoft, Genomic Steppe ancestry in skeletons from the Neolithic Single Grave Culture in Denmark. *PLOS ONE* **16**, e0244872 (2021).

228. R. Iversen, "Was there ever a single grave culture in Eastern Denmark? Traditions and transformations in the 3rd millennium BC," in *Transitional Landscapes? The 3rd Millennium BC in Europe*, M. Furholt, R. Grossmann, M. Szmyt, Eds. (Dr. Rudolf Habelt GmbH, 2016), pp. 159–170.

229. J. L. Cardoso, The Bell-beaker complex in Portugal: An overview. *O Arqueólogo Portugués Série V* , 275–308 (2016).

230. A. M. S. Bettencourt, La Edad del Bronce en el Noroeste de la Península Ibérica: Un análisis a partir de las prácticas funerarias. *Trabajos de Prehistoria* **67**, 139–173 (2010). [The Bronze Age in the Northwest of the Iberian Peninsula: An analysis based on funerary practices].

231. G. A. Jiménez, M. S. Romero, The radiocarbon chronology of tholos-type megalithic tombs in iberia: Exploring diverse social trajectories. *Trabajos de Prehistoria* **78**, 277–291 (2021).

232. V. S. Gonçalves, *Cascais Há 5000 Anos* (Câmara Municipal, Cascais, 2005). [Cascais 5000 Years Ago].

233. A. C. Valera, *Bela Vista 5: Um Recinto Do Fimnal Do 3o Milénio a.n.e. (Mombeja, Beja)* (Núcleo de Investigaçao Arqueológica - NIA, 2014). [Bela Vista 5: A Precinct From the End of the 3rd Millennium B.C.E.].

234. I. Soriano, A. M. Herrero-Corral, R. Garrido-Pena, T. Majó, Sex/gender system and social hierarchization in Bell Beaker burials from Iberia. *J. Anthropol. Archaeol.* **64**, 101335 (2021).

235. T. X. Schuhmacher, "Ivory Exchange Networks in the Chalcolithic of the Western Mediterranean," in *Key Resources and Sociocultural Developments in the Iberian Chalcolithic*, M. Bartelheim, P. Bueno Ramírez, M. M. Kunst, Eds. (Tübingen Library Publishing, 2017), pp. 291–312.

236. J. Zilhão, A. M. M. Soares, A. P. Gonçalves, Sperm-whale V-perforated buttons from Galeria da Cisterna (Almonda Karst System, Torres Novas, Portugal). *Trabajos de Prehistoria* **79**, 131–140 (2022).

237. J. L. Cardoso, Absolute chronology of the Beaker phenomenon North of the Tagus estuary: Demographic and social implications. *Trabajos de Prehistoria* **71**, 56–75 (2014).

238. J. M. Vergès, E. Allué, M. Fontanals, J. I. Morales, P. Martín, Á. Carrancho, I. Expósito, M. Guardiola, M. Lozano, R. Marsal, X. Oms, I. Euba, A. Rodríguez, El Mirador cave (Sierra de Atapuerca, Burgos, Spain): A whole perspective. *Quat. Intern.* **414**, 236–243 (2016).

239. A. Szécsényi-Nagy, C. Roth, G. Brandt, C. Rihuete-Herrada, C. Tejedor-Rodríguez, P. Held, Í. García-Martínez-De-Lagrán, H. Arcusa Magallón, S. Zesch, C. Knipper, E. Bánffy, S. Friederich, H. Meller, P. Bueno Ramírez, R. Barroso Bermejo, R. De Balbín Behrmann, A. M. Herrero-Corral, R. Flores Fernández, C. Alonso Fernández, J. Jiménez Echevarria, L. Rindlisbacher, C. Oliart, M. I. Fregeiro, I. Soriano, O. Vicente, R. Micó, V. Lull, J. Soler Díaz, J. A. López Padilla, C. Roca De Togores Muñoz, M. S. Hernández Pérez, F. J. Jover Maestre, J. Lomba Maurandi, A. Avilés Fernández, K. T. Lillios, A. M. Silva, M. Magalhães Ramalho, L. M. Oosterbeek, C. Cunha, A. J. Waterman, J. Roig Buxó, A. Martínez, J. Ponce Martínez, M. Hunt Ortiz, J. C. Mejías-García, J. C. Pecero Espín, R. Cruz-Auñón Briones, T. Tomé, E. Carmona Ballestero, J. L. Cardoso, A. C. Araújo, C. Liesau, Von Lettow-Vorbeck, C. Blasco Bosqued, P. Ríos Mendoza, A. Pujante, J. I. Royo-Guillén, M. A. Esquembre Beviá, V. M. Dos Santos Goncalves, R. Parreira, E. Morán Hernández, E. Méndez Izquierdo, J. Vega Y Miguel, R. Menduiña García, V. Martínez Calvo, O. López Jiménez, J. Krause, S. L. Pichler, R. Garrido-Pena, M. Kunst, R. Risch, M. A. Rojo-Guerra, W. Haak, K. W. Alt, The maternal genetic make-up of the Iberian Peninsula between the Neolithic and the Early Bronze Age. *Sci. Rep.* **7**, 15644 (2017).

240. R. Garrido-Pena, R. Flores Fernández, A. M. Herrero-Corral, *Las Sepulturas Campaniformes de Humanejos (Parla, Madrid)* (Comunidad de Madrid, 2019). [The Bell-Shaped Tombs of Humanejos].

241. R. J. Harrison, A. Mederos Martín, "Bell Beakers and Social Complexity in Central Spain," in *Bell Beakers Today: Pottery, People, Culture, Symbols in Prehistoric Europe (Riva Del Garda, 1998)*, F. Nicolis, Ed. (Provincia Autonoma di Trento, 2001), pp. 111–124.

242. P. Bueno Ramírez, R. Barroso Bermejo, R. De Balbín Behrmann, Ritual campaniforme, ritual colectivo: La necrópolis de cuevas artificiales del Valle de las Higueras, Huecas, Toledo. *Trabajos de Prehistoria* **62**, 67–90 (2005). [Bell Beaker Ritual, Collective Ritual: The Necropolis of Artificial Caves in the Valley of the Fig Trees, Huecas, Toledo].

243. J. Lomba Maurandi, M. López Martínez, F. Ramos Martínez, A. Avilés Fernández, El enterramiento múltiple, calcolítico, de Camino del Molino (Caravaca, Murcia). Metodología y primeros resultados de un yacimiento excepcional. *Trabajos de Prehistoria* **66**, 143–159

(2009). [The Chalcolithic multiple burial site of Camino del Molino (Caravaca, Murcia). Methodology and initial results from an exceptional site].

244. O. García Puchol, J. Bernabeu Aubán, Y. Carrión Marco, L. Molina Balaguer, G. Pérez Jordà, M. Gómez Puche, A funerary perspective on Bell Beaker period in the Western Mediterranean. Regarding the social context of individual burials at La Vita (Gandía, Valencia). *Trabajos de Prehistoria* **70**, 332–345 (2013).

245. J. Lomba Maurandi, M. Haber Uriarte, "El registro funerario calcolítico en el extremo suroriental de la Península Ibérica: Los valles del Guadalentín y Segura (Murcia)," in *Del Neolític a l'edat de Bronze En El Meditarrani Occidental. Estudis En Homenatge a Bernat Martí Oliver*, M. de P. de V.-D. de València, Ed. (Diputación de Valencia, 2016), pp. 349–364. [The Chalcolithic funerary record in the southeastern corner of the Iberian Peninsula: The Guadalentín and Segura valleys (Murcia)].

246. R. Garrido-Pena, *El Campaniforme En La Meseta Central de La Peninsula Ibérica (c. 2500-2000 AC)* (BAR Publishing, 2000). [The Bell Tower in the Central Plateau of the Iberian Peninsula (c. 2500-2000 BC)].

247. P. Ríos, C. Blasco, R. Aliaga, Entre el Calcolítico Y la Edad del Bronce. Algunas consideraciones sobre la cronología campaniforme. *Cuadernos de Prehistoria y. Arqueología* **38**, 195–208 (2012). [Between the Chalcolithic and the Bronze Age. Some considerations on the Bell Beaker chronology].

248. O. Lemercier, Y. Tchérémissinoff, "Du Néolithique final au Bronze ancien: Les sépultures individuelles campaniformes dans le sud de la France," in *Les Sépultures Individuelles Campaniformes En France*, L. Salanova, Y. Tchérémissinoff, Eds. (CNRS Éditions, 2011), pp. 177–194. [From the Late Neolithic to the Early Bronze Age: Individual Bell Beaker Burials in Southern France].

249. O. Lemercier, "The Beaker transition in Mediterranean France," in *Backgroud to Beakers. Inquiries into Regional Cultural Backgrounds of the Bell Beaker Complex*, H. Fokkens, F. Nicolis, Eds. (Sidestone Press, 2012), pp. 117–156.

250. M. Vander Linden, For Whom the Bell Tolls: Social Hierarchy vs Social Integration in the Bell Beaker Culture of Southern France (Third Millennium BC). *Camb. Archaeol. J.* **16**, 317 (2006).

251. J. Cauliez, "The Bell Beaker complex: A vector of transformations? Stabilities and changes of the indigenous cultures in South-East France at the end of the Neolithic period," in *The Bell Beaker Transition in Europe. Mobility and Local Evolution during the 3rd Millnnium BC*, M. P. P. Martínez, L. Salanova, Eds. (Oxbow Books, 2015), pp. 88–112.

252. J. Fernández-Eraso, E. Arévalo Muñoz, C. Camarero Arribas, J. A. Mujika Alustiza, "El campaniforme en la Rioja Alavesa," in *¡Un Brindis Por El Príncipe! El Vaso Campaniforme En El Interior de La Península Ibérica (2500-2000 A.C.)*, G. Delibes de Castro, Ed. (Museo Arqueológico Regional, 2019), pp. 279–296. [The Bell Beaker in Rioja Alavesa].

253. J. Fernández-Eraso, E. Arevalo-Muñoz, C. Camarero Arribas, M. García-Diez, B. Ochoa Fraile, J. A. Mujika-Alustiza, Estela decorada en el dolmen del Alto de la Huesera (Laguardia, Álava). *Zephyrus* **78**, 19–33 (2016). [Decorated stele in the Alto de la Huesera dolmen (Laguardia, Álava)].

254. M. Lipson, A. Szécsényi-Nagy, S. Mallick, A. Pósa, B. Stégmár, V. Keerl, N. Rohland, K. Stewardson, M. Ferry, M. Michel, J. Oppenheimer, N. Broomandkhoshbacht, E. Harney, S. Nordenfelt, B. Llamas, B. G. Mende, K. Köhler, K. Oross, M. Bondár, T. Marton, A. Osztás, J. Jakucs, T. Paluch, F. Horváth, P. Csengeri, J. Koós, K. Sebok, A. Anders, P. Raczky, J. Regenye, J. P. Barna, S. Fábián, G. Serlegi, Z. Toldi, E. G. Nagy, J. Dani, E. Molnár, G. Pálfi, L. Márk, B. Melegh, Z. Bánfai, L. Domboróczki, J. Fernández-Eraso, J. A. Mujika-Alustiza, C. A. Fernández, J. J. Echevarría, R. Bollongino, J. Orschiedt, K. Schierhold, H. Meller, A. Cooper, J. Burger, E. Bánffy, K. W. Alt, C. Lalueza-Fox, W. Haak, D. Reich, Parallel palaeogenomic transects reveal complex genetic history of early European farmers. *Nature* **551**, 368–372 (2017).

255. J. Guilaine, F. Claustre, O. Lemercier, P. Sabatier, "Campaniformes et environnement culturel en France méditerranéenne," in *Bell Beakers Today: Pottery, People, Culture, Symbols in Prehistoric Europe (Riva Del Garda, 1998)*, F. Nicolis, Ed. (Provincia

Autonoma di Trento, 2001), pp. 229–275. [Bell Beakers and Cultural Environment in Mediterranean France].

256. A. M. Colligá, “État de la question du campaniforme dans le contexte culturel chalcolithique du Nord-Est de la péninsule Ibérique,” in *Bell Beakers Today: Pottery, People, Culture, Symbols in Prehistoric Europe (Riva Del Garda, 1998)*, F. Nicolis, Ed. (Provincia Autonoma di Trento, 2001), pp. 155–171. [State of the Bell Beaker Question in the Chalcolithic Cultural Context of the Northeast of the Iberian Peninsula].

257. Y. Tchérémissinoff, G. Escalon, R. Donat, “Le coffre lithique campaniforme ou épicampaniforme du site Georges Besse II-5, Nîmes (Gard),” in *Les Sépultures Individuelles Campaniformes En France*, L. Salanova, Y. Tchérémissinoff, Eds. (CNRS Éditions), pp. 167–176. [The Bell-shaped or Epicampaniform lithic chest from the Georges Besse II-5 site, Nîmes (Gard)].

258. J. Guilaine, *La Civilisation Du Vase Campaniforme Dans Les Pyrénées Françaises* (Gabelle, 1967). [The Bell Beaker Vase Civilization in the French Pyrenees].

259. J. Guilaine, “La civilisation des vases campaniformes dans le Midi de la France,” in *Glockenbecher Symposion. Oberried 1974*, J. Lanting, J. D. Van der Waals, Eds. (Fibula-Van Dishoeck, 1976), pp. 351–370. [La civilisation des vases campaniformes dans le Midi de la France].

260. O. Lemercier, “The Bell Beaker question: From historical-cultural approaches to aDNA analyses,” in *Demography and Migration. Population Trajectories from the Neolithic to the Iron Age. Proceedings of the XVII UISPP World Congress (4-9 June 2018, Paris, France). Volume 5. Sessions XXXII-2 and XXXIV-8*, T. Lachenal, R. Roure, O. Lemercier, Eds. (Archaeopress, 2020), pp. 116–140.

261. L. Salanova, “Chronologie et facteurs d’évolution des sépultures individuelles campaniformes dans le Nord de la France,” in *Les Sépultures Individuelles Campaniformes En France*, L. Salanova, Y. Tchérémissinoff, Eds. (CNRS Éditions, 2011), pp. 125–142. [Chronology and factors of evolution of individual Bell Beaker burials in the North of France].

262. Q. Favrel, C. Nicolas, Bell Beaker burial customs in North-western France. *Proc. Prehist. Soc.* **88**, 285–320 (2022).

263. L. Salanova, "Behind the warriors: Bell Beakers and identities in Atlantic Europe (Third millennium B. C.)," in *Celtic from the West 3. Atlantic Europe in the Metal Ages - Questions of Shared Language*, J. I. Koch, B. Cunliffe, Eds. (Oxbow Books, 2016), pp. 13–34.

264. S. Needham, Transforming Beaker Culture in North-West Europe; Processes of Fusion and Fission. *Proc. Prehist. Soc.* **71**, 171–217 (2005).

265. M. Vander Linden, "Bell Beaker pottery and society," in *The Oxford Handbook of Neolithic Europe*, C. Fowler, D. Hofmann, Eds. (Oxford Univ. Press, 2015), pp. 605–620.

266. N. Carlin, *The Beaker Phenomenon. Understanding the Character and Context of Social Practices in Ireland* (Sidestone Press, 2018).

267. N. Patterson, M. Isakov, T. Booth, L. Büster, C. E. Fischer, I. Olalde, H. Ringbauer, A. Akbari, O. Cheronet, M. Bleasdale, N. Adamski, E. Altena, R. Bernardos, S. Brace, N. Broomandkhoshbacht, K. Callan, F. Candilio, B. Culleton, E. Curtis, L. Demetz, K. S. D. Carlson, C. J. Edwards, D. M. Fernandes, M. G. B. Foody, S. Freilich, H. Goodchild, A. Kearns, A. M. Lawson, I. Lazaridis, M. Mah, S. Mallick, K. Mandl, A. Micco, M. Michel, G. B. Morante, J. Oppenheimer, K. T. Özdoğan, L. Qiu, C. Schattke, K. Stewardson, J. N. Workman, F. Zalzala, Z. Zhang, B. Agustí, T. Allen, K. Almássy, L. Amkreutz, A. Ash, C. Baillif-Ducros, A. Barclay, L. Bartosiewicz, K. Baxter, Z. Bernert, J. Blažek, M. Bodružić, P. Boissinot, C. Bonsall, P. Bradley, M. Brittain, A. Brookes, F. Brown, L. Brown, R. Brunning, C. Budd, J. Burmaz, S. Canet, S. Carnicero-Cáceres, M. Čaušević-Bully, A. Chamberlain, S. Chauvin, S. Clough, N. Čondić, A. Coppa, O. Craig, M. Črešnar, V. Cummings, S. Czifra, A. Danielisová, R. Daniels, A. Davies, P. de Jersey, J. Deacon, C. Deminger, P. W. Ditchfield, M. Dizdar, M. Dobeš, M. Dobisíková, L. Domboróczki, G. Drinkall, A. Đukić, M. Ernée, C. Evans, J. Evans, M. Fernández-Götz, S. Filipović, A. Fitzpatrick, H. Fokkens, C. Fowler, A. Fox, Z. Gallina, M. Gamble, M. R. González Morales, B. González-Rabanal, A. Green, K. Gyenesei, D. Habermehl, T. Hajdu, D. Hamilton, J. Harris, C. Hayden, J. Hendriks, B. Hernu, G. Hey, M. Horňák, G. Ilon, E. Istvánovits, A. M. Jones, M. B. Kavur, K. Kazek, R. A. Kenyon, A. Khreisheh, V. Kiss, J.

Kleijne, M. Knight, L. M. Kootker, P. F. Kovács, A. Kozubová, G. Kulcsár, V. Kulcsár, C. Le Pennec, M. Legge, M. Leivers, L. Loe, O. López-Costas, T. Lord, D. Los, J. Lyall, A. B. Marín-Arroyo, P. Mason, D. Matošević, A. Maxted, L. McIntyre, J. McKinley, K. McSweeney, B. Meijlink, B. G. Mende, M. Menđušić, M. Metlička, S. Meyer, K. Mihovilić, L. Milasinovic, S. Minnitt, J. Moore, G. Morley, G. Mullan, M. Musilová, B. Neil, R. Nicholls, M. Novak, M. Pala, M. Papworth, C. Paresys, R. Patten, D. Perkić, K. Pesti, A. Petit, K. Petriščáková, C. Pichon, C. Pickard, Z. Pilling, T. D. Price, S. Radović, R. Redfern, B. Resutík, D. T. Rhodes, M. B. Richards, A. Roberts, J. Roefstra, P. Sankot, A. Šefčáková, A. Sheridan, S. Skae, M. Šmolíková, K. Somogyi, Á. Somogyvári, M. Stephens, G. Szabó, A. Szécsényi-Nagy, T. Szeniczey, J. Tabor, K. Tankó, C. T. Maria, R. Terry, B. Teržan, M. Teschler-Nicola, J. F. Torres-Martínez, J. Trapp, R. Turle, F. Ujvári, M. van der Heiden, P. Veleminsky, B. Veselka, Z. Vytlačil, C. Waddington, P. Ware, P. Wilkinson, L. Wilson, R. Wiseman, E. Young, J. Zaninović, A. Žitňan, C. Lalueza-Fox, P. de Knijff, I. Barnes, P. Halkon, M. G. Thomas, D. J. Kennett, B. Cunliffe, M. Lillie, N. Rohland, R. Pinhasi, I. Armit, D. Reich, Large-scale migration into Britain during the Middle to Late Bronze Age. *Nature* **601**, 588–594 (2022).

268. M. Parker Pearson, A. Sheridan, M. Jay, A. Chamberlain, M. P. Richards, J. Evans, *The Beaker People: Isotopes, Mobility and Diet in Prehistoric Britain* (Oxbow Books, 2019).

269. D. L. Clarke, *Beaker Pottery of Great Britain and Ireland* (Cambridge Univ. Press, 1970).

270. M. Parker Pearson, “Economy and society in Beaker-period Britain,” in *The Beaker People: Isotopes, Mobility and Diet in Prehistoric Britain, M. Parker Pearson*, A. Sheridan, M. Jay, A. Chamberlain, M. P. Richards, J. Evans, Eds. (Oxbow Books, 2019), pp. 81–114.

271. A. Barclay, C. Halpin, *Excavations at Barrow Hills, Radley, Oxfordshire. Volume 1 The Neolithic and Bronze Age Monument Complex* (Oxford Archaeological Unit, 1999).

272. M. Jay, M. P. Richards, P. Marshall, “Radiocarbon dates and their Bayesian modelling,” in *The Beaker People: Isotopes, Mobility and Diet in Prehistoric Britain*, M. Parker Pearson, A. Sheridan, M. Jay, A. Chamberlain, M. P. Richards, J. Evans, Eds. (Oxbow Books, Oxford, 2019), pp. 43–80.

273. N. Curtis, N. Wilkin, M. Hutchison, "Beakers and bodies in north-east Scotland: A regional and contextual study," in *The Beaker People: Isotopes, Mobility and Diet in Prehistoric Britain*, M. Parker Pearson, A. Sheridan, M. Jay, A. Chamberlain, M. P. Richards, J. Evans, Eds. (Oxbow Books, 2019), pp. 211–252.

274. H. Vandkilde, A review of the early late neolithic period in Denmark: Practice, identity and connectivity. *J. Neolithic Archaeol.* **7**, 10.12766/jna.2005.13 (2005).

275. T. Sarauw, Danish Bell Beaker pottery and flint daggers – The display of social identities? *Eur. J. Archaeol.* **11**, 23–47 (2009).

276. H. Vandkilde, "Beaker representation in the Danish Late Neolithic," in *Bell Beakers Today: Pottery, People, Culture, Symbols in Prehistoric Europe (Riva Del Garda, 1998)*, F. Nicolis, Ed. (Provincia Autonoma di Trento, 2001), pp. 333–360.

277. L. Salanova, Y. Tchérémissinoff, "Conclusion générale. Impact des pratiques funéraires campaniformes en France," in *Les Sépultures Individuelles Campaniformes En France*, L. Salanova, Y. Tchérémissinoff, Eds. (CNRS Éditions, 2011), pp. 195–199. [General conclusion. Impact of Bell Beaker funeral practices in France].

278. R. Schwarz, "Kultureller Bruch oder Kontinuität? - Mitteldeutschland im 23. Jh. v. Chr.," in *2200 BC – Ein Klimasturz Als Ursache Für Den Zerfall Der Alten Welt? 7. Mitteldeutscher Archäologentag Vom 23. Bis 26. Oktober 2014 in Halle (Saale)*, H. Meller, H. W. Arz, R. Jung, R. Risch, Eds. (Landesamt für Denkmalpflege und Archäologie Sachsen-Anhalt - Landesmuseum für Vorgeschichte, 2015), pp. 671–713. [Cultural break or continuity? - Central Germany in the 23rd century BC].

279. M. Fröhlich, M. Becker, "Typochronologische Überlegungen zu den Kulturen des Endneolithikums und der frühen Bronzezeit zwischen Saale und Unstrut im 3. Jt. v. Chr.," in *2200 BC – Ein Klimasturz Als Ursache Für Den Zerfall Der Alten Welt? 7. Mitteldeutscher Archäologentag Vom 23. Bis 26. Oktober 2014 in Halle (Saale)*, H. Meller, H. W. Arz, R. Jung, R. Risch, Eds. (Landesamt für Denkmalpflege und Archäologie Sachsen-Anhalt, Landesmuseum für Vorgeschichte, 2015), pp. 765–782.

[Typochronological considerations on the cultures of the late Neolithic and the early Bronze Age between the Saale and Unstrut rivers in the 3rd millennium BC].

280. A. Selent, F. Koch, Archäologie an der Ortsumfahrung Hettstedt im Mansfelder Land. *Archäologie Sachsen Anhalt* , 98–133 (2012). [Archaeology at the Hettstedt bypass in the Mansfelder Land].

281. K. Schwerdtfeger, "Der Großgrabhügel - multikulturelle Ruhestätte über Jahrhunderte hinweg," in *Archäologie XXL. Archäologie an Der B 6n Im Landkreis Quedlingburg*, H. Meller, Ed. (Landesamt für Denkmalpflege und Archäologie Sachsen-Anhalt - Landesmuseum für Vorgeschichte, 2006), pp. 106–111. [The large burial mound - a multicultural resting place for centuries].

282. A. Hille, *Die Glockenbecherkultur in Mitteldeutschland* (Landesamt für Denkmalpflege und Archäologie Sachsen-Anhalt - Landesmuseum für Vorgeschichte, 2012). [The Bell Beaker culture in Central Germany].

283. U. Müller, "Die Kinder von Rothenschirmbach," in *Archäologie Auf Der Überholspur. Ausgrabungen an Der A38*, H. Meller, Ed. (Landesamt für Denkmalpflege und Archäologie Sachsen-Anhalt, Landesmuseum für Vorgeschichte, 2006), pp. 98–107. [The children of Rothenschirmbach].

284. A. Moser, M. Fröhlich, "Wie Perlen an einer Schnur - Gräber der Glockenbecherkultur in Oechlitz, Saalekreis," in *Neue Gleise Auf Alten Wegen II. Jüdendorf Bis Gröbers*, H. Meller, Ed. (Landesamt für Denkmalpflege und Archäologie Sachsen-Anhalt, Landesmuseum für Vorgeschichte, 2017), pp. 315–330. [Like pearls on a string - graves of the Bell Beaker culture in Oechlitz, Saalekreis].

285. J. N. Lanting, De NO-Nederlandse/NW-Duitse klokbekergroep: Culturele achtergrond, typologie van het aardewerk, datering, verspreiding en grafritueel. *Palaeohistoria* , 11–326 (2008). [The NE Netherlands/NW Germany Bell Beaker Group: Cultural background, typology of the pottery, dating, distribution and burial ritual].

286. M. Conrad, Die naturwissenschaftliche Datierung des Glockenbechergrabes von Eythra/ Zwenkau (ZW-01), Lkr. Leipzig. *Arbeits- und Forschungsberichte zur Sächsischen Bodendenkmalpflege* **57/58**, 47–52 (2019). [The scientific dating of the Bell Beaker grave of Eythra/Zwenkau (ZW-01), Leipzig district].

287. J. Turek, "The Bell Beaker culture," in *The Prehistory of Bohemia 3. The Eneolithic*, E. Neustupný, M. Dobeš, J. Turek, M. Zápotocký, Eds. (Archeologický Ústav AV ČR, 2013), pp. 154–178.

288. P. Limburský, *Pohřebiště Kultury Se Zvoncovitými Poháry ve Vlíněvsi. K Problematice a Chronologii Konce Eneolitu a Počátku Doby Bronzové* (Univerzita Karlova v Praze, Filozofická Fakulta, 2012). [The Bell-shaped Beaker Culture Burial Site in Vlíněves. On the Issues and Chronology of the Late Eneolithic and Early Bronze Age].

289. J. Havel, Pohřební ritus kultury zvoncovitých pohárů v Čechách a na Moravě. *Praehistorica* **7**, 91–117 (1978). [Funerary rite of the Bell-shaped Beaker culture in Bohemia and Moravi].

290. L. Kreiner, Neue Gräber der Glockenbecherkultur aus Niederbayern. *Bayerische Vorgeschichtsblätter* **56**, 151–161 (1991). [New graves of the Bell Beaker culture from Lower Bavaria].

291. R. Tichý, J. Turek, I. Dohnálková, H. Dohnálkova, R. Thér, Birituální pohřebiště kultury zvoncovitých pohárů v cihelně Tuněchody u Chrudimi. Předběžná zpráva o výzkumu pohřebiště. *Acta archaeologica Opaviensia* **3**, 45–60 (2008). [A biritual burial site of the Bell-shaped Beaker culture in the Tuněchody brickyard near Chrudim. Preliminary report on the research of the burial site].

292. J. Turek, "Significance of cremation in the funerary practices of the Bell Beaker Eastern Province," in *Bell Beaker in Everyday Life. Proceedings of the 10th Meeting "Archéologie et Gobelets" (Florence – Siena – Villanuova Sul Clisi, May 12-15, 2006)*, M. Baioni, V. Leonini, D. Lo Vetro, F. Martini, R. Poggiani Keller, L. Sarti, Eds. (Museo Fiorentino di Preistoria Paolo Graziosi, 2008), pp. 271–280.

293. J. Turek, Období zvoncovitých pohárů v Evropě. *Archeologie ve Stredních Cechách* **10**, 275–368 (2006). [The Bell-shaped Cup Period in Europe. Archaeology in Central Bohemia 10].

294. M. Kruťová, "Bell Beaker and Únětice Burial Rites. Continuity and Change in Funerary Practices at the Beginning of Bronze Age," in *The Northeast Frontier of Bell Beakers: Proceedings of the Symposium Held at the Adam Mickiewicz University, Poznań (Poland), May 26-29 2002*, J. Czebreszuk, M. Szmyt, Eds. (British Archaeological Report International Series 1155, Archaeopress, 2003), pp. 209–214.

295. A. Czene, "The Position of the Bell Beaker-Csepel Group at Budakala'sz," in *State of the Hungarian Bronze Age Research. Proceedings of the Conference Held between 17th and 18th of December 2014*, G. Kulcsár, G. V Szabo, Eds. (Institute of Archaeology, Research Centre for the Humanities, Hungarian Academy of Sciences, 2017), pp. 179–199.

296. M. Furmanek, A. Haługzko, M. Mackiewicz, B. Myślecki, "New data for research on the Bell Beaker Culture in Upper Silesia, Poland," in *2200 BC – Ein Klimasturz Als Ursache Für Den Zerfall Der Alten Welt? 7. Mitteldeutscher Archäologentag Vom 23. Bis 26. Oktober 2014 in Halle (Saale)*, H. Meller, H. W. Arz, R. Jung, R. Risch, Eds. (Landesamt für Denkmalpflege und Archäologie Sachsen-Anhalt - Landesmuseum für Vorgeschichte, 2015), pp. 525–538.

297. J. Budziszewski, P. Włodarczak, *Kultura Pucharów Dzwonowatych Na Wyżynie Małopolskiej* (Instytut Archeologii i Etnologii PAN, Oddzial w Krakowie, 2010). [Bell Beaker Culture in the Lesser Poland Upland].

298. P. Makarowicz, "Northern and Southern Bell Beakers in Poland," in *The Northeast Frontier of Bell Beakers. Proceedings of the Symposium Held at the Adam Mickiewicz University, Poznań (Poland), May 26-29 2002*, J. Czebreszuk, M. Szmyt, Eds. (British Archaeological Report International Series 1155, Archaeopress, 2003), pp. 137–154.

299. E. Kazdová, M. Kuča, K. Šabatová, F. Trampota, L. Prokeš, M. Hložek, J. Kolář, J. Petřík, Kyjovice (okr. Znojmo). Sutny II. KZP. Hrob. Systematický výzkum. *Prehled Vyzkumu* **52**, 180–181 (2011). [Sutny II. KZP. Grave. Systematic research].

300. A. Czene, "“Henge-artiges”-Denkmal und Bestattungsplatz der Glockenbecherzeit in Poysbrunn, Niederösterreich," in *Trassenarchäologie 3, Sonderheft 1*, K. Fiebig, A. Csaplaros, Eds. (ArchaeoProtect GmbH, 2019). ["Henge-like" monument and burial site of the Bell Beaker period in Poysbrunn, Lower Austria].

301. J. Peška, Neue absolute Datierungen aus dem späten Äneolithikum Mährens. *Offa* **63**, 7–44 (2011). [New absolute dating from the late Eneolithic of Moravia].

302. J. Peška, "Absolutní datování hrobů z období KZP z Hoštic I a ze Záhlinic I," in *Pohřebiště z Období Zvoncovitých Pohárů Na Trase Dálnice D1 Vyškov - Mořice [Funerary Areas of the Bell Beaker Period on the D1 Vyškov - Mořice Motorway]. Pravěk NŘ, Supplementum 24*, P. Dvořák, A. Matějíčková, Eds. (Ústav Archeologické Památkové Péče, 2012), pp. 153–166. [Absolute dating of graves from the KZP period from Hoštice I and Záhlinice I].

303. B. Metzinger-Schmitz, *Die Glockenbecherkultur in Mähren Und Niederösterreich. Typologische Und Chronologische Studien Auf Dem Hintergrund Der Kulturhistorischen Abläufe Während Der Späten Kupferzeit Im Untersuchungsgebiet. Mit Einem Paläometallurgischen Exkurs* (Universität des Saarlandes, 2004). [The Bell Beaker Culture in Moravia and Lower Austria: Typological and Chronological Studies Against the Background of Cultural and Historical Developments During the Late Copper Age in the Study Area].

304. R. Patay, "Bell Beaker cemetery and settlement at Szigetszentmiklós: First results," in *Transitions to the Bronze Age: Interregional Interaction and Socio-Cultural Change in the Third Millennium BC Carpathian Basin and Neighbouring Regions*, V. Heyd, G. Kulcsár, V. Szeverényi, Eds. (Archaeolingua, 2013), pp. 287–317.

305. A. Matějíčková, P. Dvořák, Pohřebiště z období zvoncovitých pohárů na trase dálnice D1 Vyškov - Mořice [Funerary areas of the Bell Beaker period on the D1 Vyškov - Mořice motorway]. Pravěk NŘ, Supplementum 24. Pravěk NŘ, Supplementum 24 [Preprint] (2012).

306. K. Ottományi, A. Czene, "Az M0-s autópálya északi szakaszának feltárása, Budakalász. Budakalász-Csajerszke (Mrt 7, 3/12. lh.)," in *Régészeti Kutatások Másfél Millió*

Négyzetméteren. Autópálya És Gyorsforgalmi Építését Megelőző Régészeti Feltárások Pest Megyében 2001–2006, E. Tari, Ed. (Pest Megyei Múzeumi Füzetek Új Sorozat, 2006), vol. 7, pp. 69–73. [Exploration of the northern section of the M0 motorway, Budakalász. Budakalász-Csajerszke (Mrt 7, 3/12. lh.)].

307. J. Peška, "Two new burial sites of Bell Beaker Culture with an exceptional finds from Eastern Moravia/Czech Republic," in *Current Researches on Bell Beakers. Proceedings of the 15th International Bell Beaker Conference: From Atlantic to Ural, 5th-9th May 2011, Poio (Pontevedra, Galicia, Spain)*, M. Pilar Prieto Martínez, L. Salanova, Eds. (Copynino-Centro de Impresión Digital, 2013), pp. 61–72.

308. A. Kośko, "Z badań nad udziałem tradycji kultury amfor kulistych w sferze światopoglądowo-obrzędowej społeczeństw kultury iwieńskiej," in *Miscellanea Archaeologica Thaddaeo Malinowski Dedicata*, F. Różnowski, Ed. (Sorus, 1993), pp. 215–223. [From the research on the participation of the tradition of the globular amphora culture in the worldview and ritual sphere of the Ivano-Frankivsk culture societies].

309. P. Dvořák, I. Rakovský, J. Stuchlíková, Pohřebiště lidu s kulturou se zvoncovitými poháry u Záhlinic, okr. Kroměříž. *Pravěk NŘ* **2**, 215–232 (1992). [Burial site of people with bell-shaped cup culture near Záhlinice, Kroměříž district].

310. P. Dvořák, A. Matějíčková, J. Peška, I. Rakovský, *Gräberfelder Der Glockenbecherkultur in Mähren II. Katalog Der Funde* (Dvořák Verlag Brno, 1996). [Burial Fields of the Bell Beaker Culture in Moravia II].

311. A. Endrődi, "Funerary Rituals, Social Relations and Diffusion of Bell Beaker Csepel-Group," in *Current Researches on Bell Beakers. Proceedings of the 15th International Bell Beaker Conference: From Atlantic to Ural. 5th - 9th May 2011 Poio (Pontevedra, Galicia, Spain)*, P. Prieto Martínez, L. Salanova, Eds. (Galician ArchaeoPots, 2013), pp. 73–88.

312. J. van der Plicht, C. Bronk Ramsey, T. J. Heaton, E. M. Scott, S. Talamo, Recent developments in calibration for archaeological and environmental samples. *Radiocarbon* **62**, 1095–1117 (2020).

313. M. Furholt, Re-integrating Archaeology: A Contribution to aDNA studies and the migration discourse on the 3rd millennium BC in Europe. *Proc. Prehist. Soc.* **85**, 115–129 (2019).

314. M. Besse, C. Strahm, "The components of the Bell Beaker Complex," in *Bell Beakers Today: Pottery, People, Culture, Symbols in Prehistoric Europe: Proceedings of the International Colloquium Riva Del Garda (Trento, Italy) 11-16 May 1998*, F. Nicolis, Ed. (Provincia Autonoma di Trento, Servizio Beni Culturali, Ufficio Beni Archeologic, 2001), vol. 1, pp. 103–110.

315. Q. Bourgeois, *Monuments on the Horizon. The Formation of the Barrow Landscape throughout the 3rd and 2nd Millennium BC* (Sidestone Press, 2013).

316. J. Zilhão, Radiocarbon evidence for maritime pioneer colonization at the origins of farming in west Mediterranean Europe. *Proc. Natl. Acad. Sci. U.S.A.* **98**, 14180–14185 (2001).

317. C. S. Larsen, *Bioarchaeology: Interpreting Behavior from the Skeleton* (Cambridge Univ. Press, ed. 2, 2015).

318. A. Szczepanek, Z. Belka, P. Jarosz, Ł. Pospieszny, J. Dopieralska, K. M. Frei, A. Rauba-Bukowska, K. Werens, J. Górski, M. Hozer, M. Mazurek, P. Włodarczak, Understanding FINAL NEOLITHIC COMmunities in south-eastern Poland: New insights on diet and mobility from isotopic data. *PLOS ONE* **13**, e0207748 (2018).
